# Human versus Robots in the Discovery and Crystallization of Gigantic Polyoxometalates

**DOI:** 10.1002/anie.201705721

**Published:** 2017-08-03

**Authors:** Vasilios Duros, Jonathan Grizou, Weimin Xuan, Zied Hosni, De‐Liang Long, Haralampos N. Miras, Leroy Cronin

**Affiliations:** ^1^ WEST Chem School of Chemistry University of Glasgow University Avenue Glasgow G12 8QQ UK

**Keywords:** cluster compounds, crystallization, human strategies, machine-learning, polyoxometalates

## Abstract

The discovery of new gigantic molecules formed by self‐assembly and crystal growth is challenging as it combines two contingent events; first is the formation of a new molecule, and second its crystallization. Herein, we construct a workflow that can be followed manually or by a robot to probe the envelope of both events and employ it for a new polyoxometalate cluster, Na_6_[Mo_120_Ce_6_O_366_H_12_(H_2_O)_78_]⋅200 H_2_O (**1**) which has a trigonal‐ring type architecture (yield 4.3 % based on Mo). Its synthesis and crystallization was probed using an active machine‐learning algorithm developed by us to explore the crystallization space, the algorithm results were compared with those obtained by human experimenters. The algorithm‐based search is able to cover ca. 9 times more crystallization space than a random search and ca. 6 times more than humans and increases the crystallization prediction accuracy to 82.4±0.7 % over 77.1±0.9 % from human experimenters.

Understanding the supramolecular self‐assembly of complex inorganic molecules poses a difficult problem since it relies on two contingent events.^1^ To make a discovery the conditions under which the building blocks assemble have to be found and then the conditions under which the product aggregates into crystals to be isolated and characterized need to be identified. The vast number of combinations of the experimental conditions and the coordination modes of the transition metals taking part in the building blocks means that a full exploration of the chemical space of any given compound would be impossible.^2^ For these reasons, the intuition of experienced chemists is required to design the appropriate experiments to determine the right conditions for the isolation of any new products.^3^ But intuitions can be biased by both the current knowledge of the field and the frame of mind of the experimenter—making important discoveries difficult to achieve.

Herein, we design and investigate a new approach for probing the envelope of both the synthesis and the crystallization process of a new polyoxometalate compound with the formula Na_6_[Mo_120_Ce_6_O_366_H_12_(H_2_O)_78_]⋅200 H_2_O (**1**) {Mo_120_Ce_6_} (Figure 1). Our method is drawn from recent advances for active data acquisition in the field of machine learning, known as active learning.^4^ Active learning consists of methodologies able to decide what experiments to perform next in order to optimally improve the understanding of the system at hand. We compare our algorithmic method with a random screening process in the exploration and modelling of the crystallization conditions of compound (**1**). Importantly, we study how human experimenters approached this specific problem and compare their strategies and performance to our machine‐learning approach.

**Figure 1:**
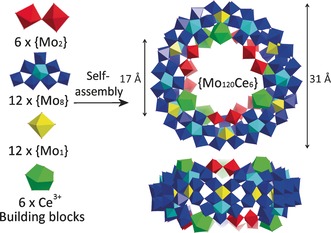
Schematic representation of the self‐assembly of the {Mo_120_Ce_6_} wheel from basic building blocks in polyhedron mode. Coloring code: {Mo_2_} red; {Mo_8_} blue with central atom in cyan; {Mo_1_} yellow; Ce green.

So far, work in this area has been mainly focused on simulations and only a few studies have involved real experiments.^5^ For example, recently, Wicker and Cooper^6^ applied machine learning methods to draw a map of crystallinity according to the size of a molecule and its number of rotatable bonds. Similarly, Oliynyk et al.^7^ used machine learning to predict structures of inorganic binary compounds of the general formula AB by considering various atomic and physical properties in their calculations. Of particular interest, Norquist et al.^8^ made use of data from unsuccessful syntheses to predict reaction outcomes of vanadium compounds and compared the efficiency of their algorithms with the typical strategies that human chemists apply.

Our machine learning approach actively defines new experiments to perform with an aim to improve its model of the system. Such targeted data acquisition strategy allows a reduction in the number of experiments needed to attain the same model quality, thus saving time and financial resources. To our knowledge, it is the first time that such an active data acquisition strategy is applied in this context and compared with human experimenters. Machine learning methods have previously been used as a tool of optimization^9^ and a faster data mining technique for extensive databases.^10^, ^11^, ^12^, ^13^, ^14^ It is important to note that our approach should not be mistaken for high‐throughput screening as it uses machine learning techniques capable of abstracting problems rather than a brute force increase of processing speed. We instead suggest this approach should be viewed as “intelligent throughput” since not all the possible experiments are done, and only those chosen by the algorithm are explored and the system effectively learns as the experiment continues similar to how an expert chemist would work.

We first introduce the compound that was discovered, the reaction conditions from which it can be isolated and characterized. We then compare our machine‐learning approach against random screening and human experimenters in terms of performance and methodologies for the exploration of the crystallization boundaries (see Figure 2).

**Figure 2:**
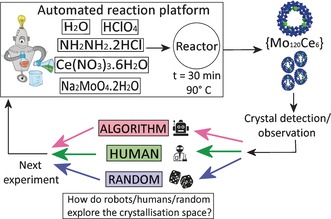
Representation of the experimental method showing how the automated and bench work was done. Structure: Mo blue; Ce green.

The new polyoxometalate cluster belongs to the family of lanthanide‐doped molybdenum blues.^15^, ^16^, ^17^, ^18^, ^19^, ^20^, ^21^ Compound **1** is isostructural to the reported^22^ Na_6_[Mo_120_Pr_6_O_366_(H_2_O)_78_H_12_]⋅ca. 200 H_2_O, but notably was first discovered automatically by our automated chemical robot, see Supporting Information: Experimental Section, Method A. In later experiments we also reproduced the synthesis and crystallization of the compound on the bench, see Supporting Information: Experimental Section, Method B. Compound **1** was characterized by elemental analysis, single‐crystal X‐ray structure analysis, bond valence sum (BVS) calculations, IR and visible‐NIR spectroscopy, redox titrations and thermogravimetry.

The single‐crystal X‐ray structure analysis reveals four of the dodecameric ring‐shaped clusters **1** in the unit cell, packed parallel to the crystallographic *bc* plane giving rise to 1D channels occupied by guest water molecules (Figure S3 in the Supporting Information). The framework of **1** consists of 12 sets of basic building blocks {Mo_8_}, {Mo_2_} and {Mo_1_} units, which are well‐defined in Mo Blue clusters such as the archetypal {Mo_154_},^22^ {Mo_176_},^23^ and {Mo_368_},^24^ with 6 {Mo_2_} units substituted by 6 Ce^III^ ions. On the whole, the architecture of **1** is constructed from 12 {Mo_8_} units, 6 {Mo_2_} units, 12 {Mo_1_} units and 6 {Ce(H_2_O)_5_} units (Figure 1). The coordination configuration of the two distinct types of Ce^III^ can be described as a distorted monocapped square antiprism, built from four μ_2_‐O atoms and five H_2_O molecules that is, {Ce(H_2_O)_5_}. Bond lengths of molybdenum atoms coordinated to terminal oxo groups have a Mo=O bond length in the range of 1.554(12)–1.702(9) Å. The symmetric arrangement of 3 Ce^III^ ions on both the upper and lower surfaces of {Mo_120_Ce_6_} greatly reduces the symmetry of **1** to *D*_3_ as compared with the parent {Mo_154_} (*D*_7*d*_ point group). As a result, the wheel displays an irregular ring‐shaped structure with an outer ring diameter of about 31 Å and an inner ring diameter of about 17 Å. A further characteristic of the structure of **1** is the large number of protons resulting from the 24 e^−^ reduction. The overall reduction state of **1** was confirmed using three independent techniques: UV/Vis spectroscopy, redox titration and bond valence sum calculations (BVS), [see Supporting Information for details]. BVS calculations^25^ are carried out on all the Mo and O centers (Table S2). A careful analysis of the BVS result reveals 12 singly and 78 doubly protonated oxygen atoms. Taking into consideration the obtained information from the above calculations along with elemental analysis and redox titrations, it is possible to determine the overall building‐block scheme and overall charge for compound **1**: [{Mo_2_}_6_{Mo_1_}_12_{Mo_8_}_12_{Ce_6_}]≡[{Mo^VI^_2_O_5_(H_2_O)_2_}_6_{Mo^VI/V^_8_O_26_(μ_3_‐O)_2_H(H_2_O)_3_Mo^VI/V^}_12_ {Ce^III^(H_2_O)_5_}_6_]^6−^.

To explore the synthetic and crystallization process it is important to define the process of the reaction accurately as shown in Figure 2. By describing an abstract method we could then turn this into a concrete procedure and then output the precise set of experiments to perform, determined by either a human or the algorithm‐driven robot using three methods; robot‐algorithm; human; and robot‐random as the control method. For the experimental conditions to be defined and explored three distinct pieces of information must be provided: 1) the chemicals involved in the synthesis, 2) an experimental method for the synthesis and crystallization process, and 3) an initial set of data consisting of successful and failed crystallization experiments, that is, the starting information used to decide what experiments to perform next, see Figure 3. Next, to compare the methods using a commonly calibrated and therefore robust experimental test, we developed an automated platform (Figure S8) able to consistently perform the crystallization experiments given a list of parameters such as the number of reagents and their corresponding volumes (see Supporting Information, part 7).

**Figure 3:**
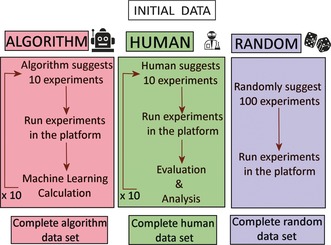
Schematic diagram of the exploration methods used in our studies comparing the algorithmic approach with that of the human experimenter and a random approach. Both the random and algorithmic approaches used a purpose‐built liquid handling and crystallization robotic platform.

For the reactions, aqueous stock solutions of Na_2_MoO_4_⋅2 H_2_O 1 m, Ce(NO_3_)_3_⋅6 H_2_O 0.1 m, NH_2_NH_2_⋅2 HCl 0.25 m and HClO_4_ 1 m were prepared and used as described in the Supporting Information: Experimental Section, Method A. During the experiment both Na_2_MoO_4_⋅2 H_2_O and Ce(NO_3_)_3_⋅6 H_2_O were always added in a 1:1 volume ratio (molar ratio 10:1 respectively). The automated platform mixes, in a reactor, the stock solutions in configurable ratios to a total volume of 15 mL, and allows them to react at 90 °C for 30 min. We then collect a 9 mL sample for crystallization and then use a cleaning protocol to reset the system before the next experiment begins. The crystallization method consists of waiting overnight to allow for crystals to form. Finally, the presence or absence of crystals is checked under illumination with a white light emitting diode (3300–3500 lux at a distance of 5 cm) and the information is added in a database of experiments. It is important to note that under these particular conditions, no other product crystallizes in a time frame longer than one month, see Supporting Information, Table S12. Following this process the initial data set was obtained from previous experiments performed in the platform and is shown in Figure 4, also see Supporting Information, section 7, Table S3. Single‐crystal X‐ray diffraction analysis confirmed that the main product is cluster (**1**). The resulting data set, consisting of 89 points, was provided to each method and served as the initial database and training ground for their subsequent exploration.

**Figure 4:**
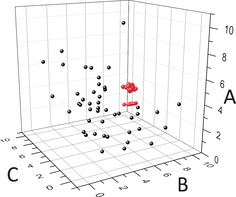
3D graph of the initial set of data. A) Na_2_MoO_4_⋅2 H_2_O 1 m and Ce(NO_3_)_3_⋅6 H_2_O 0.1 m (mL); B) HClO_4_ 1 m (mL); C) NH_2_NH_2_⋅2 HCl 0.25 m (mL). Crystals red; non‐crystals black.

As described above, we studied three different methods: a machine‐learning algorithm approach, human experimenters and random experiments as a baseline (Figure 3). Each method followed the same protocol: 1) analyze the dataset of the previous experiments, 2) specify 10 new experiments to execute, 3) receive a crystal/no‐crystal information for each of the requested experiments. The process is repeated 10 times for a total of 100 experiments. At each iteration, all data collected previously are integrated in the decision process for generating the next set of 10 experiments. All experiments for all methods were executed on the platform under similar conditions. Each method was then evaluated in terms of strategies and overall exploration of the experimental space. The change in the ratio of the chemicals not only provides us with information of the experimental conditions that a given compound crystallizes in, which is the thermodynamic outcome of the most favorable conformation, but it also provides information on which combinations of reagents are not successful in the formation of compound (**1**). The latter can lead to the observation of other known compounds [see Supporting Information, section 8] or it can lead to the discovery of new chemical species. Figure 5 shows the difference in the crystal quality as we move from the central cluster of the initial data shown in Figure 4 to the outer regions of the crystallization boundaries.

**Figure 5:**
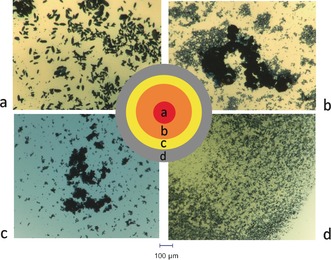
Change in the crystal quality of the crystallization sphere as we move from the initial data set (a), to the middle of the boundaries (b), and the outer edges of the boundaries (c). d) shows the precipitate which is observed when moving further away from the initial data set.

The machine‐learning approach is based on established active learning algorithm in classification problems^26^ whereby the classification is the process of assigning labels (e.g. crystal/no‐crystal) to regions of a parameters space (e.g. a range of experimental parameters) given only a few examples of known label‐parameters instances. The quality of such model depends on the quality of the training data, the complexity and non‐linearity of the process studied as well as its stochasticity. Methodologies have been developed to decide which experiments to perform in order to improve the model faster, called active learning procedures.^4^ Our algorithm is strongly inspired by such methods but adapted to the particular problem under investigation, see Supporting Information, part 6. Human experimenters were volunteers among PhD students in our group, all familiar with inorganic chemistry synthesis, and hence could be considered already to be “experts”. For the needs of this study, they were aware of the chemical formula of compound (**1**), the reagents, the reaction conditions, the platform and the initial set of data. They were not aware of the overall aim of comparing strategies among methods. Each human experimenter was instructed to develop their own strategy given the objective to identify the range of experimental conditions where compound (**1**) can be isolated. A baseline method is used as control, it consists of selecting experiments at random in the chemical space. This method is thus blind to both the initial and the subsequently collected crystallization information. To determine the differences between methods and their respective overall data acquisition performance we qualitatively analyzed trends in exploration strategies between each method. Then we quantified the number of experiments leading to crystals, as well as the extent of chemical space that was explored by each method. Using this data, it is then possible to compute the effectiveness of respective crystallization models and their predictive power.

The difference between human experimenters and the algorithm is illustrated in Figure S28. We can observe the stepwise exploration of human experimenters, starting from the known core of initial data provided and expanding outwards (S28, c). The limiting factor in their exploration is that when they perform an experiment that yields no‐crystals, they stop after trying only a handful of experiments further than this point. Additionally, human experimenters tend to construct investigations that are more “Cartesian” shown in Figure S28,c, which reflects practical constrains. On the other hand, the algorithm follows a more “polar” approach around the initial set of data (Figure S28,b) and is not “disturbed” when crystals are not formed in the experiments, allowing a wider area to be covered.

Following the results from our experiments, in Figure 6 we plot the average explored volume of the experimental space as a function of the number of experiments performed. For the volume calculation, we compute the volume of the convex envelope of the experiments leading to crystals, see Supporting Information, part 10.2.2. We observe a large difference between algorithm and human experimenters. This can be explained because the algorithm is agnostic to the chemical environment and untied with prior chemical knowledge. Additionally, the algorithm is more “adventurous”, performing “jumps” in the chemical space straight into the believed boundaries between crystal and no‐crystal. On the contrary, human experimenters have drastically varied strategies depending on personal perceptions and biases of the particular chemistry involved. Figures S35–S38 provide a visual representation of the experiments selected by the two human experimenters. Other exploration metrics confirm the increased exploration of our active learning approach as well as the high variability between human experimenters, see Supporting Information, part 11.2.3.

**Figure 6:**
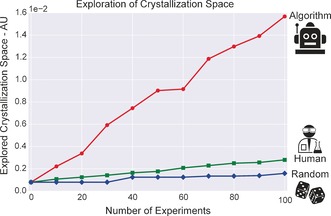
Explored crystallization space by the three methods. The exploration is computed as the volume of the convex envelop of the experiments leading to crystals [see Supporting Information, part 10.2.2].

Additionally, human experimenters can be baited by the absolute number of crystallization points they discover, disregarding how conservative or not their strategy can be. This point is important because a conservative strategy with many small “steps” of exploration can lead to many crystallization points but limits a wider exploration of the chemical space. For example, the second human experimenter performed an impressive 47 experiments leading to crystallization, out of 100 (Table 1, run 2). But to characterize our system the breadth of exploration is more important that the absolute numbers of crystal formulation found. On the other hand, the algorithm, despite finding only 32 crystal experiments, revealed more about the chemical landscape. As shown in Figures S43 and S45, it was able to discover and explore a third of the crystallization region and spend a significant number of experiments exploring non‐intuitive formulations.

**Table 1:** Total number of crystal points found for all runs of the three methods applied.

Method	Run 1	Run 2
Algorithm	27	32
Human experimenter	26	47
Random	4	2

Finally, the main interest of using a machine learning approach is that the data acquisition process can be informed and coupled with the objective—in this case, building an accurate crystallization map. We tested that hypothesis by computing and testing a model of our crystallization system at each iteration and for each method (average of both runs) [see Supporting Information, section 10]. The quality of the prediction, that is, the percentage of time a crystal prediction is accurate, is expected to increase as more data are collected. Figure 7 shows that the machine‐learning algorithm was able to collect much better quality data and improved its classification accuracy from 68.1 % (i.e. the initial prediction quality based on the initial database provided to all methods) to 82.4±0.7 %. Whereas the humans showed a less significant improvement (from 68.1 % to 77.1±0.9 %) and the random method did not improve in accuracy (from 68.1 % to 68.7±1.4 %). The fact that the model computed using the human method improves less should be considered in light of the new data acquired, human experimenters simply did not collect as useful information as the algorithm method. This is even more striking with the random method that provided no additional information. These results were computed using a different classifier than the one used within the algorithm method, in order to verify that the data collected were not tied to the underlying assumptions of the algorithm. Results with other classifiers are presented in Supporting Information, section 10.3 and confirm the trends from Figure 7.

**Figure 7:**
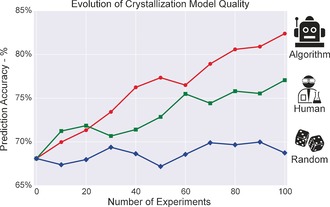
Average for the prediction accuracies between the classes of crystals and non‐crystals for the three methods, using a RandomForest classifier [see Supporting Information, part 11.3].

In previous studies the data used to characterize and model crystallization processes were extracted from databases of experiments intended for other research purposes. Here, we coupled the data acquisition process with the modeling of our system, with the aim of characterizing the crystallization boundaries of a new polyoxometalate cluster (compound **1**) in real time. Our “intelligent‐throughput” is powerful since it combines both intuition from machine learning and reliable liquid handling, allowing the system to develop “chemical‐intuition”; we hypothesize this could be a first step to developing a new approach we term chemical intelligence which uses machine learning to explore complex chemical systems. Using this approach we could observe significant differences in the strategies not only between the algorithm and the human experimenters but also between the two human experimenters. These differences can have a significant impact in the ability of exploration and are heavily dependent on the personal and chemical biases of the individual. In the future, we aim to explore how to combine the intuition of the chemists with chemical intelligence to use human–machine teams to identify new phenomena and characterize new chemical systems. We have placed the code online^27^ in the hope that this will help others who wish to use machine learning in crystal chemistry.

## Conflict of interest

The authors declare no conflict of interest.

## Supporting information

As a service to our authors and readers, this journal provides supporting information supplied by the authors. Such materials are peer reviewed and may be re‐organized for online delivery, but are not copy‐edited or typeset. Technical support issues arising from supporting information (other than missing files) should be addressed to the authors.

Supplementary
