# Supplementary material for: Human versus Robots in the Discovery and Crystallization of Gigantic Polyoxometalates
Source: Angew Chem Int Ed Engl. 2017 Aug 3;56(36):10815–20. doi: 10.1002/anie.201705721 (PMC5577512; doi:10.1002/anie.201705721)
Supplement: Supplementary file 1 — Supplementary [file ANIE-56-10815-s001.pdf]

Angewandte Chemie
Eine Zeitschrift der Gesellschaft Deutscher Chemiker

Supporting Information

Human versus Robots in the Discovery and Crystallization of Gigantic Polyoxometalates

*Vasilios Duros[+], Jonathan Grizou[+], Weimin Xuan, Zied Hosni, De-Liang Long, Haralampos N. Miras, and Leroy Cronin**

anie_201705721_sm_miscellaneous_information.pdf

Table of contents

1.	Single crystal X-ray diffraction characterization	S3
2.	Vis – NIR spectroscopy	S6
3.	Thermogravimetric analysis	S7
4.	Redox titrations	S7
5.	Synthetic procedures	S8
6.	Algorithm principles, implementation, and simulations	S12
6.1.	Principles	S12
6.2.	Uncertainty sampling: Implementation details and tailoring to our problem	S15
6.2.1.	Uncertainty sampling	S18
6.2.2.	Modification for batch sampling	S22
6.2.3.	Performances	S26
6.3.	A second example	S27
6.4.	Limitations and Discussions	S29
7.	Initial set of data	S30
8.	Analysis of experiments performed between methods	S33
8.1	Visualization of crystallization methods	S33
8.2	Experimental protocol as developed by the human experimenters	S35
8.3	Results	S37
9.	Single-crystal X-ray diffraction validation of the products observed in the crystallization boundaries	S43
10.	ICP validation of the products observed in the crystallization boundaries	S50
11.	Quantitative analysis of the strategies	S52
11.1	Principles	S52
11.2	Explored space	S52
11.2.1.	Number of crystals found	S52
11.2.2.	Volume exploration: Convex hull method	S53
11.2.3	Similarity between experiments	S55
11.3.	Data and modelling quality	S59
11.3.1.	Principles and biases	S59
11.3.2.	Comparing methods	S60
13.	References for the Supporting Information	S63

Resources

Code: https://github.com/croningp/crystal_active_learning

1. Single crystal X-ray diffraction characterization

Suitable single crystal was selected and mounted onto a rubber loop using Fomblin oil. Single crystal X-ray diffraction data were recorded on a Bruker Apex CCD diffractometer (λ (MoK_{α}) = 0.71073 Å) at 150 K equipped with a graphite monochromator. Data collection and reduction were performed using the Apex2 software package and structure solution, and refinement was carried out by SHELXS-97[1] and SHELXL-2014[2] using WinGX[3]. Corrections for incident and diffracted beam absorption effects were applied using empirical absorption correction. All the Mo atoms (including those disordered) and most of the O atoms were refined anisotropically. Cerium ions (inside the cavity of the ring) were identified and refined anisotropically. Solvent water molecule sites with partial occupancy were found and included in the structure refinement. Crystallographic formulas typically contain much more water molecules in the crystal lattice than the formulas used for chemical analyses as the sample was dried up. The final refinement statistics are relatively good, and in all cases the structural analysis allows us to unambiguously fully determine the structure of the compound. Further details of the crystal structure investigations may be obtained from FIZ Karlsruhe, 76344 Eggenstein-Leopoldshafen, Germany (fax: (+49)7247-808-666; e-mail: crysdata@fiz-karlsruhe.de, on quoting the deposition number CSD-432715.

According to the single-crystal X-ray diffraction characterization the full formula corresponds to: $Na_6[Mo_{120}Ce_6O_{366}H_{12}(H_2O)_{78}]\cdot200H_2O$ (**1**)

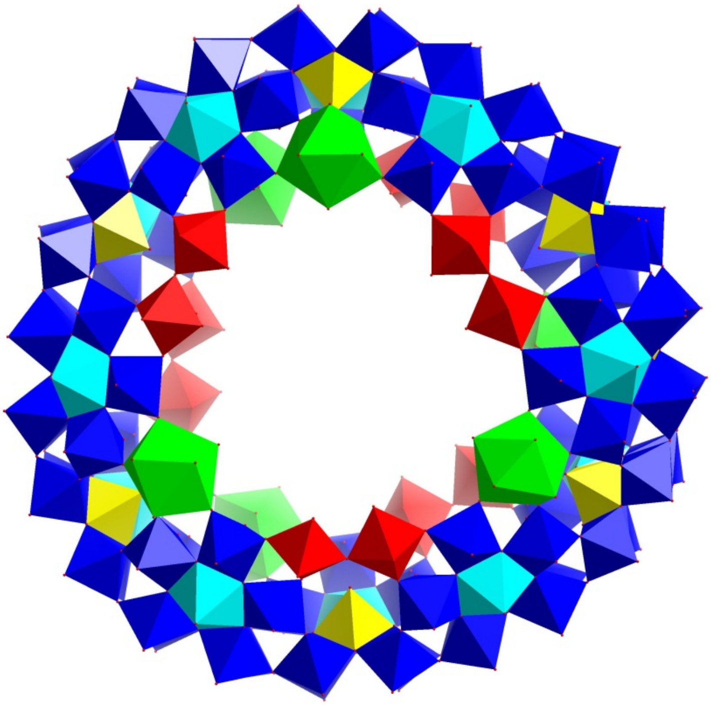

Figure S1: Representation of the {$Mo_{120}Ce_6$} wheel. Coloring code: {Mo_2}, red; {Mo_8}, blue with central atom in cyan; {Mo_1}, yellow; Ce, green.

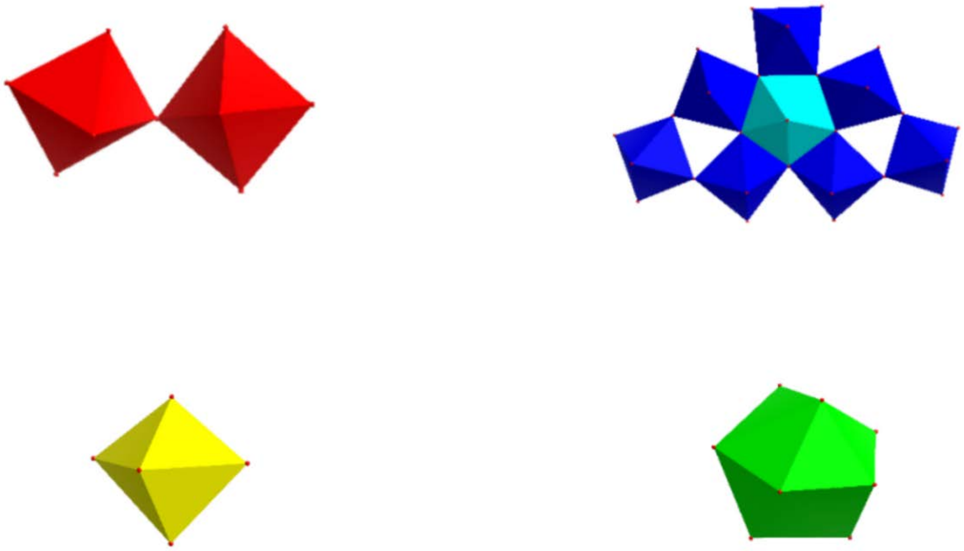

Figure S2: Representation of the building units of the $\{Mo_{120}Ce_6\}$ wheel. Top left, $\{Mo_2\}$, red; top right, $\{Mo_8\}$, blue with central atom in cyan; bottom left, $\{Mo_1\}$, yellow; bottom right, Ce, green.

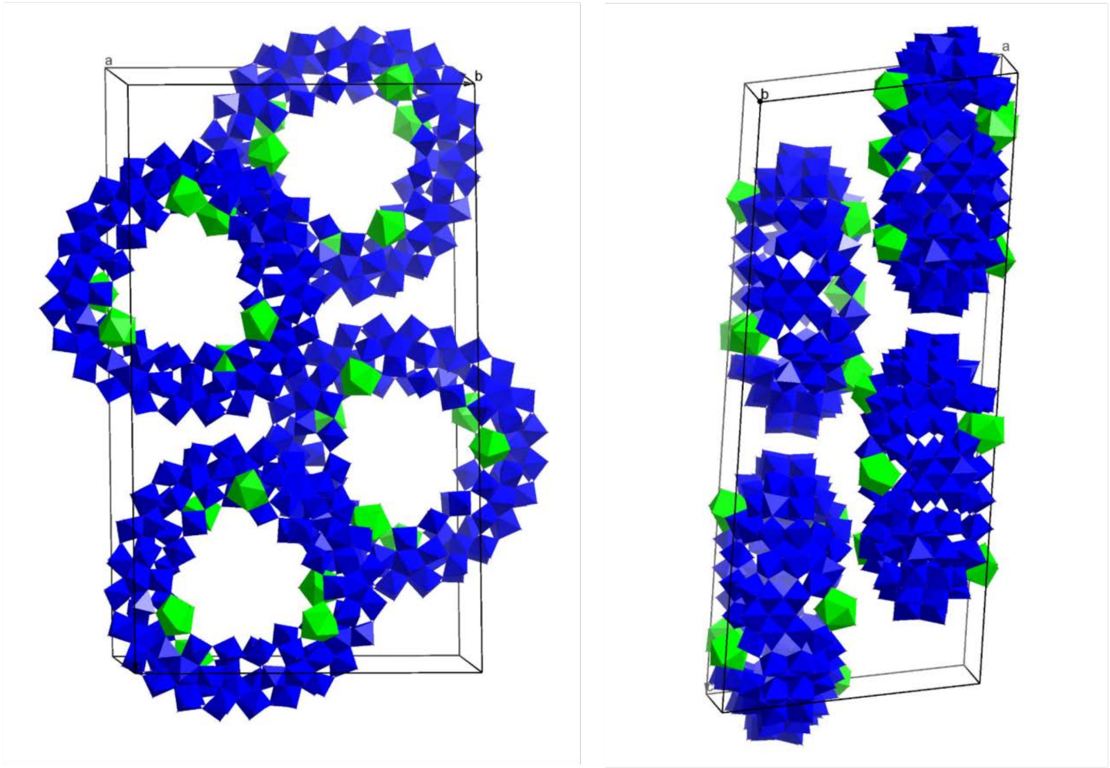

Figure S3: The dodecameric ring-shaped clusters **1** in the unit cell packed parallel to the crystallographic *bc* plane. The different building units of $\{Mo_{120}Ce_6\}$ are represented with the same color for clarity.

Table S1: Crystallographic data and structure refinement of $\{Mo_{120}Ce_6\}$ wheel.

formula	$Ce_6H_{568}Mo_{120}Na_6O_{644}$
M_r [g mol^{-1}]	23367.97
Crystal system	Monoclinic
space group	$P2_1/c$
Crystal size [mm]	0.100 x 0.070 x 0.050
a [Å]	27.6641(12)
b [Å]	38.4811(17)
c [Å]	65.261(3)
α [°]	90
β [°]	99.935(2)
γ [°]	90
h	-34≤h≤32
k	-47≤k≤43
l	-80≤l≤66
ρ [μg m^{-3}]	2.268
V [Å^3]	68431(5)
Z	4
Wavelength, λ [Å]	0.71073
μ [mm^{-1}]	2.622
T [K]	150(2)
F(000)	44696
rflns (collected)	497446
rflns (unique)	132515
Absorption correction	empirical
data/ restraints/ parameters	132515 / 2 / 6052
Refinement method	Full-matrix least-squares on F^2
R_1 (all data)	0.1180
wR_2 (all data)	0.2157
R_1 [I>2sigma(I)]	0.0734
wR_2 [I>2sigma(I)]	0.1764
R_{int}	0.0628
GooF on F^2	1.113
Largest diff. peak and hole [e.A^{-3}]	2.80 and -2.92

Table S2: Average bond valence sum values (BVS) for the Mo centres which span the incomplete $\{Mo_5O_6\}$-type double cubanes and the μ_3-O atoms of the $\{(\mu_3\text{-O})_2O_2\}$-type compartments in $\{Mo_{120}Ce_6\}$.

Compounds	BVS (Mo)	BVS (μ_3-O)
$\{Mo_{120}Ce_6\}$	5.63	1.26

2. Vis – NIR spectroscopy

Analysis of the extinction coefficient (ε) for the ligand-to-metal charge-transfer associated with the reduced Mo^{V} centres. Each centre should contribute ca. 5 -6 x 10^3 L $mol^{-1}\cdot cm^{-1}$ to ε.

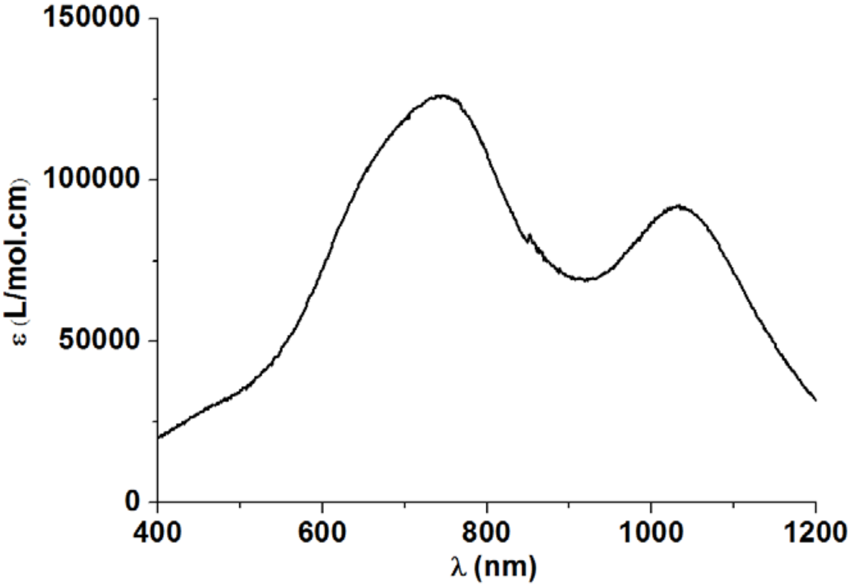

Figure S4: Vis-NIR spectrum for $\{Mo_{120}Ce_6\}$ in 0.5 M H_2SO_4 (2 × 10^{-6} mol L^{-1}). The average ε of each Mo^{V} centre is about 5.23 x 10^3 L $mol^{-1}\cdot cm^{-1}$ at 736 nm corresponding to the ligand-to-metal charge-transfer (LMCT).

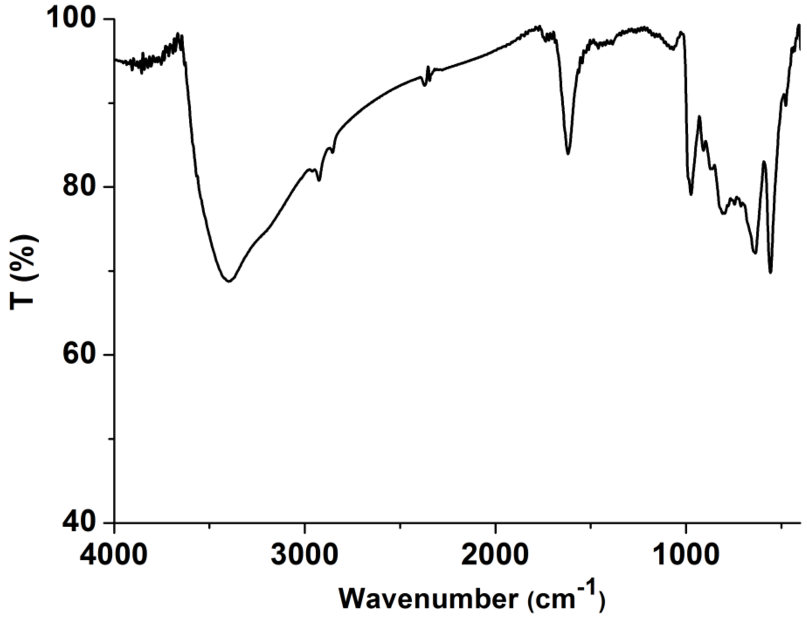

Figure S5: IR spectrum for $\{Mo_{120}Ce_6\}$. Characteristic IR bands: (KBr; 1700-500 cm^{-1}): 1618 (m), 971 (m; *v* (Mo=O)), 803 (s), 635 (s), 558 (s) cm^{-1}.

3. Thermogravimetric analysis

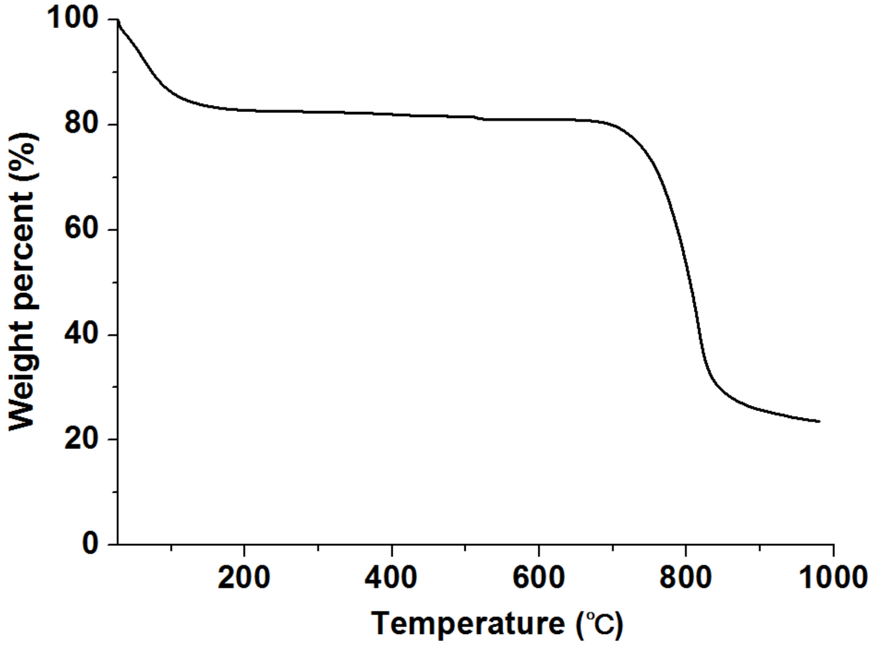

Figure S6: TGA curve for {$Mo_{120}Ce_6$}. The 16.5 % weight loss from room temperature to 200°C corresponds to approximately 210 H_2O.

4. Redox titrations

Redox titrations help to determine the number of reduced Mo^V centres. The cerimetric titration was carried out using a 0.005 M solution of Ce^{IV} in 0.5 M of sulphuric acid as oxidant which was added dropwise to a solution of compound {$Mo_{120}Ce_6$} (20 mg in 50 mL of H_2O). After addition of 4.30 mL of the oxidant the colour of the solution turned from deep blue to colourless along with characteristic potential jump showed the presence of 24 ± 1 4d electrons which (formally) corresponds to 24 Mo^V centres (theoretical value for 24 e-reduced species : 4.10 mL).

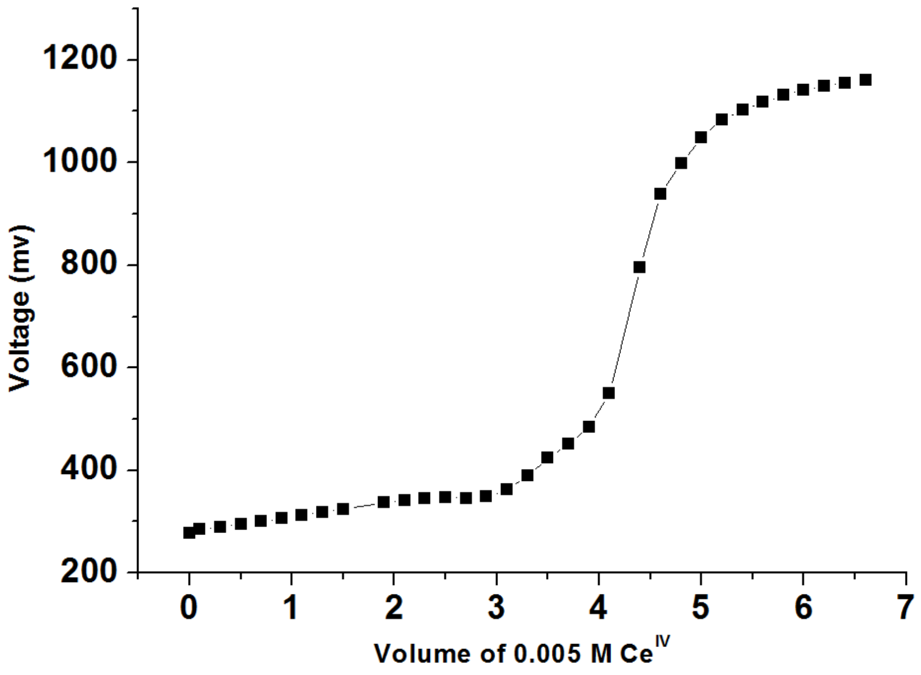

Figure S7: Redox titration of {$Mo_{120}Ce_6$} with 0.005 M Ce^{IV}.

5. Synthetic procedures

All chemicals were supplied by Sigma Aldrich and they were used without further purification.

$Na_2MoO_4.2H_2O$ [CAS: 10102-40-6]: sodium molybdate dihydrate (99%), M=241.95 g/mol; $Ce(NO_3)_3.6H_2O$ [CAS: 10294-41-4]: cerium nitrate(III) hexahydrate (99%), M=434.22 g/mol; $HClO_4$ [CAS: 7601-90-3]: perchloric acid 70%, d=1.66 g/mL; $NH_2NH_2.2HCl$ [CAS: 5341-61-7]: hydrazine dihydrochloride (98%), M=104.97 g/mol

The pump system set-up utilized 10 programmable syringe pumps (C3000 model, Tricontinent Ltd, CA, USA) fitted with a 5 mL syringe and a 3-way solenoid valve (Figure S8). Four pumps (i.e. pumps 1, 2, 3 and 4) have been designated for functions such as the washing protocol and sampling.

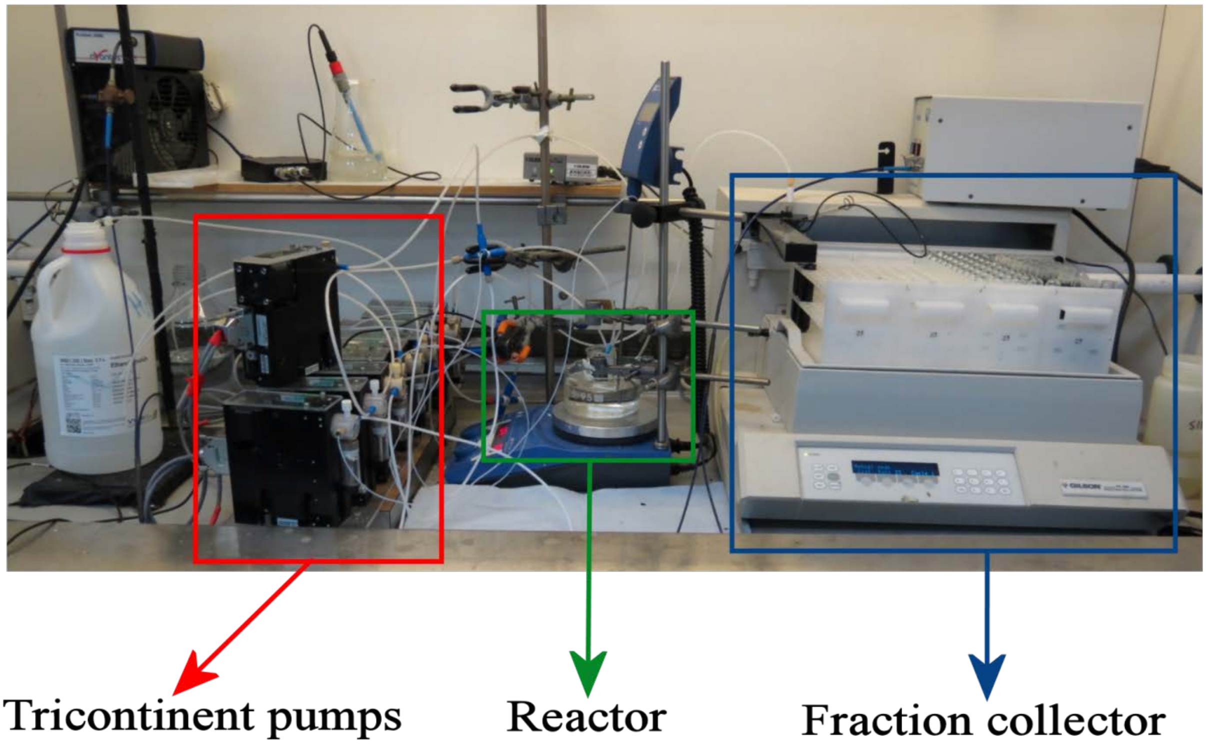

Figure S8: Image of the platform used for our experiments.

More specifically, pump 1 is connected to a deionized water tank at all times and is used for washing the plastic tubing after the sampling has been completed; pump 2 is used for the sampling from the reactor (Figure S9a) to the Gilson FC204 fraction collector; pump 3 is used for emptying the reactor before the washing and pump 4 is connected to a 4-way connector (Figure S9b) and used for switching the flow in the tubing between sampling (pump 2) and washing (pump 1). From the remaining pumps, the stock reagent solutions are assigned to pumps 5 to 9 as described in the Experimental, Method A. Pumps 7-8 are mixing the stock solutions in a 6-way connector (Figure S9c) while pump 9 is directly connected to the reactor. This was arranged in order to avoid blockage issues because of the reaction of $Na_2MoO_4.2H_2O$ and $Ce(NO_3)_3.6H_2O$ to readily form $MoO_3{\cdot}Ce_2O_3$. Finally, pump 10 (spacer) is used to pump air into the connector in order to make sure that there are no reagents left in the tubing of the 6-way connector. FEP plastic tubing 1/8" OD was cut to connect the stock solutions of reagents to the inlets of the assigned pumps by using standard HPLC low pressure PTFE connectors.

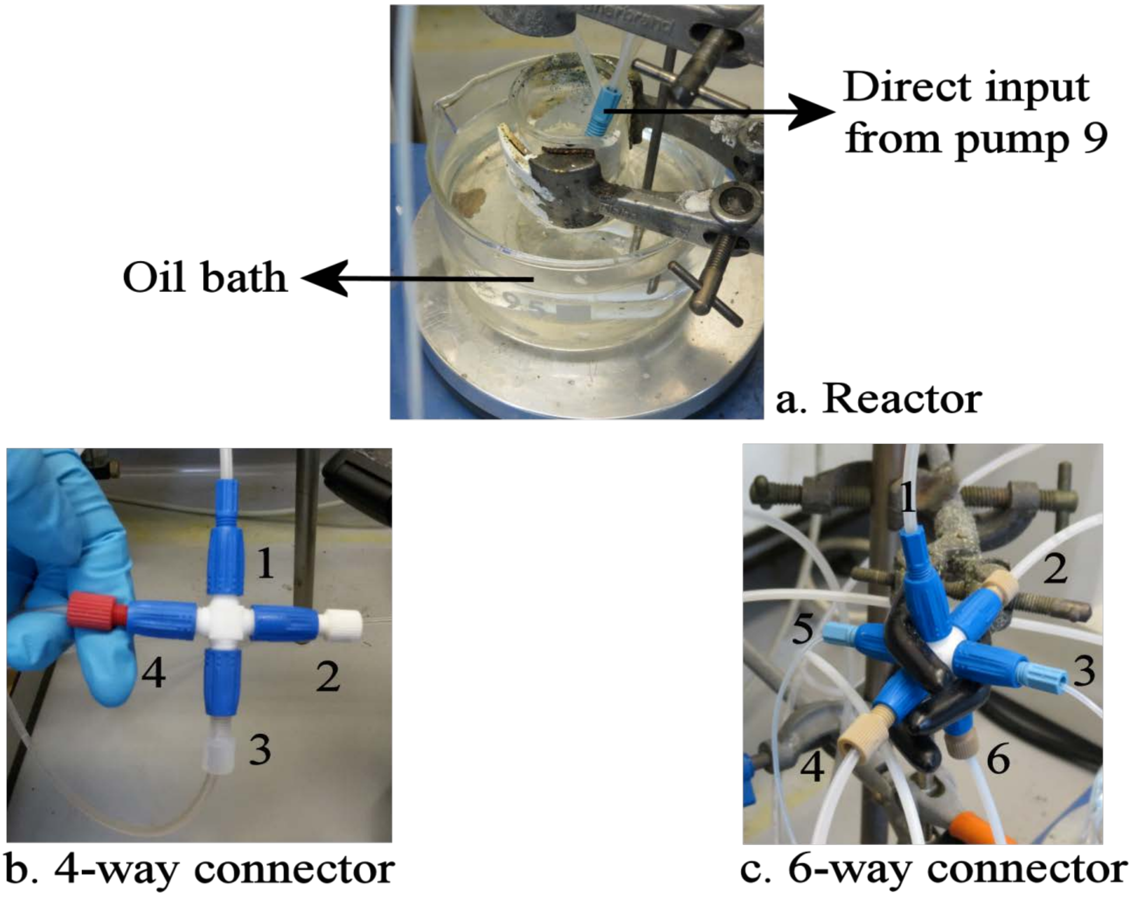

Figure S9: a) The oil bath heats the reactor to 90°C during the experiments. Pump 9 is directly connected to the reactor to avoid blockage issues. b) Position 1 is the input from pump 1 for the cleaning; position 3 is the output to pump 4 switching between sampling and washing; position 4 is the input from pump 2 performing the sampling. c) positions 3-6 are the inputs of the stock solutions described in Experimental, Method A (3 for A, 4 for C, 5 for H_2O, 6 for D); position 1 is the input of air(spacer, pump 10); position 2 is the output to the reactor

The platform is controlled by a computer using LabVIEW^{TM} based interface (Figure S10) capable to control hardware. The design of experiments is previously planned and prepared in TXT files containing the volumes of the reagents in four columns (1st for water; 2nd for reagent A; 3rd for reagent D; 4th for B and C) and, then, are introduced in the LabVIEW-based PC interface. The LabVIEW^{TM} based interface recognizes the matrix TXT files, converts the volume entries to proper command scripts and, finally, executes them to run the pumps.

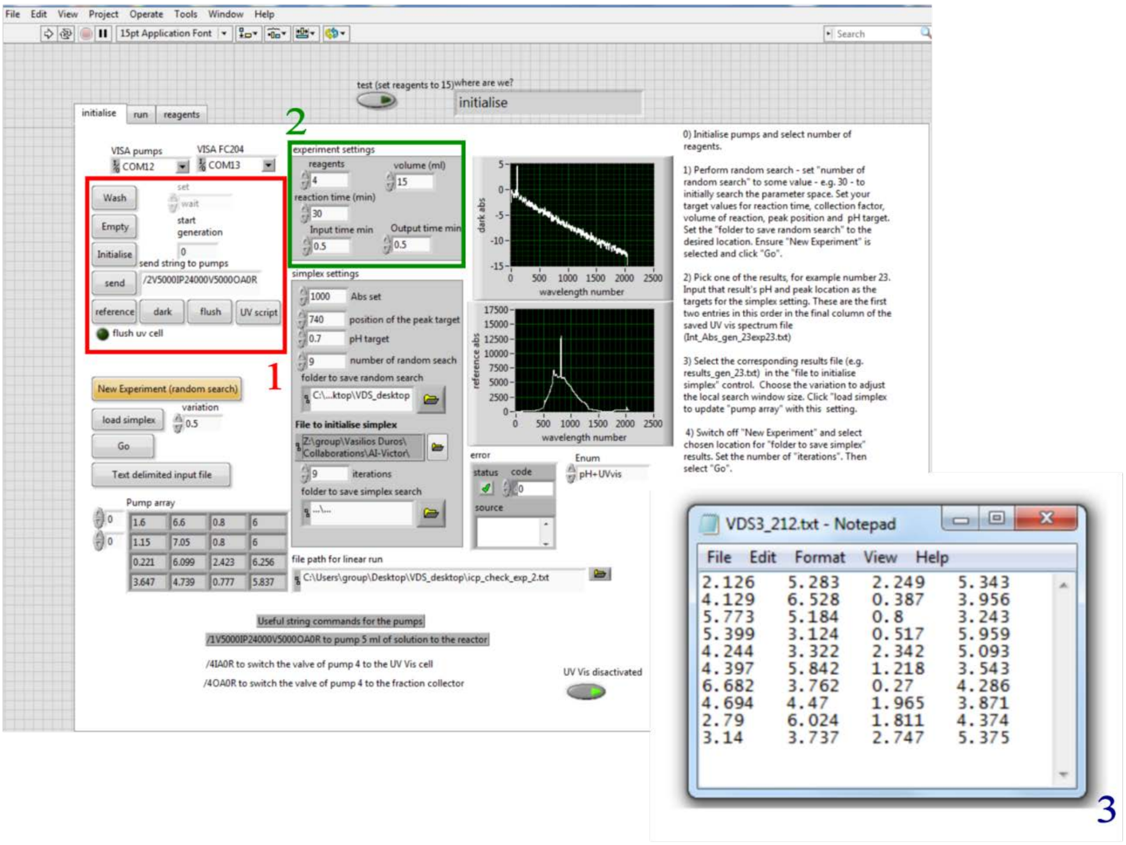

Figure S10: LabVIEW[TM] control PC interface. Functions like initializing the pumps and washing are located on rectangle (1). The experimental settings (number of reagents, total reaction volume, reaction time and pump rate) are located on rectangle (2). On (3) we can see a representative sample of a TXT file prepared for the experiments.

Synthesis details of $Na_6[Mo_{120}Ce_6O_{366}H_{12}(H_2O)_{78}]\cdot 200H_2O$ (1):

<u>Method A (synthesis in the platform):</u>

4 aqueous stock solutions were prepared as follows: 250 ml $HClO_4$ 1M (A), 500 ml $Na_2MoO_4.2H_2O$ 1M (B), 500 ml $Ce(NO_3)_3.6H_2O$ 0.1M (C) and 200 ml $NH_2NH_2.2HCl$ 0.25M (D)

Stock solutions B and C are always added in a volume ratio of 1:1. Maximum reaction volume is 15 ml.

The stock solutions along with H_2O were connected to the inlets of the assigned pumps; namely pump number 5 for H_2O, pump number 6 for A, pump number 7 for D, pump number 8 for C and pump number 9 for solution B. For this experiment, all pumps (10 in total) were active. Five pumps (no. 5-9) for the solutions of the reagents, four (no. 1-4) for functions like washing (using deionized water as solvent) and sampling and one pump (no. 10) for the space required between each reaction

(spacer). The volumetric fraction of each reagent can either be decided by an algorithm and transformed into a set of orders recognizable by the pumps or it can be provided as a list ready to be input in the software from the human experimenters. The total reaction volume (15 ml), the temperature (90°C), the number of iterations (10) and the reaction time (30 min) have been defined beforehand and loaded from the software used to control the experiment. Dark blue samples are collected automatically at the end of each iteration using a Gilson FC204 fraction collector. After 1 day we obtain dark blue, prismatic crystals of $\{Mo_{120}Ce_6\}$ (unit cell match). Yield: 0.025 g (4.31 % based on Mo). MW: 23649.94 g·mol^{-1}. IR (cm^{-1}): 1618 (m), 971 (m; ν (Mo=O)), 803 (s), 635 (s), 558 (s). Elemental analysis calcd for: Na, 0.59; Ce, 3.59; Mo, 49.3 %. Found: Na, 0.76; Ce, 3.81; Mo, 50.2 %.

Method B (in bench, adjusted from the ratios of Method A):

Solutions of $HClO_4$ 1 M (9.48 mL), $NH_2NH_2·2HCl$ 0.25 M (1.55 mL), $Na_2MoO_4·2H_2O$ 1 M (5.84 mL) and $Ce(NO_3)_3·6H_2O$ 0.1 M (5.84 mL) were added in deionized water (7.29 ml) giving a cloudy yellow solution. The reaction mixture was heated at 90 °C for 30 min, during which time the cloudy yellow solution changed to dark blue. While the solution is still hot, 18 mL are removed from the bulk solution and subsequently 6 mL of deionized water are added. The resulting solution was allowed to cool to room temperature and left undisturbed to crystallize for 1 week, after which time blue prismatic crystals suitable for X-ray diffraction analysis were obtained corresponding to $\{Mo_{120}Ce_6\}$ (unit cell match). Yield: 0.046 g (4.02% based on Mo). The spectroscopic and crystallographic data of the isolated compound are identical to Method A. Elemental analysis calcd for: Na, 0.59; Ce, 3.59; Mo, 49.3 %. Found: Na, 0.54; Ce, 3.96; Mo, 47.8 %.

6. Algorithm principles, implementation, and simulations

6.1 Principles

Our key idea is to frame a problem of exploration of a crystallization zone as an active learning problem within a classification scenario. In machine learning, classification is the process of learning the mapping between observations and categories based on previously collected examples[4]. In our case, we want to learn to predict if crystal/no crystal will occur based on experimental parameters, here ratios of reagents. A classifier is a machine learning method able to learn such a mapping from a database of already performed experiments, called the training set, which is a list of experimental parameters associated with a label crystal/no crystal depending on the outcome of the experiment.

We are interested in the acquisition of this training set. Can we acquire data in such a way that fewer experiments are needed to reach a good quality model? In other words, can we learn to classify between crystal and no crystal 'zones' faster

than by randomly accumulating experimental evidences? This concept has been framed in the 90's and can be summarized as: "a machine can achieve greater accuracy with fewer training labels if it is allowed to choose the data from which it learns" [5]. Because the machine learning algorithm ultimately builds the model, allowing it to query what information to collect next should improve the quality of the learning process. It also limits the amount of time spent performing costly experiments (in terms of human time and reagents) that were not always relevant for the derived model. This is particularly important in chemistry where each experiment can take hours to perform, wait for completion and analyse, as is the case for crystallization processes.

In this work, we used the uncertainty sampling query strategy framework for active learning in classification scenarios[6]. The key idea is that the algorithm has access to a big list of potential experiments that could be performed on the system. It then uses its current knowledge to predict the outcome of such experiments (crystal/no crystal) and evaluate its certainty/confidence about those predictions. The algorithm then selects the experiment it is less confident about, which somehow lies at the believed boundary between crystal and no crystal zones. This experiment is then performed on the real system and the outcome (crystal/no crystal) is added to the training set, thus relieving the uncertainty about this particular experiment. The process is then repeated until a final criterion is reached (time or financial budget, model performance) and is illustrated in Figure S11.

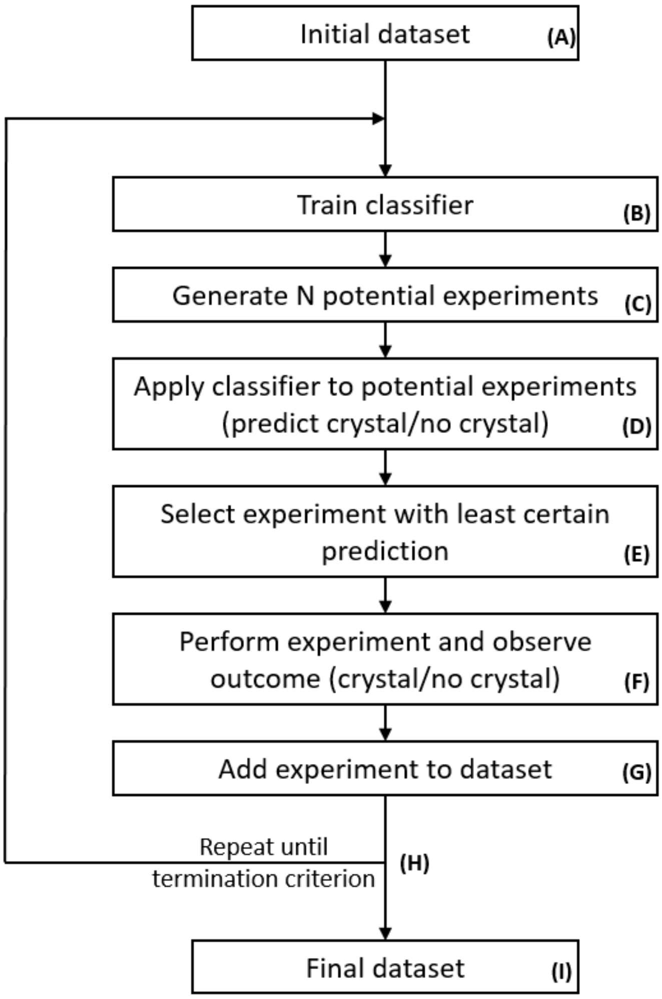

Figure S11: Algorithm steps for active learning in a classification scenario. The algorithm needs a small set of initial data (A) to train a first model (B) and generates many possible experiments to do next (C). Given the learned classifier it predicts the outcome of those experiments (D), and selects the most uncertain experiment (E), i.e. the one for which it is the least confident about its own prediction. The selected experiment is performed on the real system (F), the result is added to the dataset (G), used to train a new classifier and the process is repeated again up to a given termination criterion (H). The final dataset should be of higher quality than if collected using a non-active acquisition method (I).

In the following section, we will describe how we implemented each step of this algorithmic process in light with previous work and with respect to our particular setup and constraints inherent to chemical systems. The most important constrain being that performing and reading the outcome of an experiment takes one day, so we need to wait for crystallization to happen. Such extreme case is unusual and rarely considered in the machine learning community. As a result, we had to adapt the basic principle of uncertainty sampling to allow for sampling of 10 new experiments at each iteration, so we could perform more in one day. But a naive implementation would produce 10 very similar new experiments to perform, because

there is usually one dominant zone of high uncertainty for a given classifier. To avoid this, each experiment requested by the algorithm is reused and creates a repulsion area for the sampling of the next experiments. This is explained in more detail next, along with simulation results showing that our modified method (10 by 10 sampling) maintains comparable performances compared with the original (1 by 1 sampling) method.

6.2 Uncertainty sampling: Implementation details and tailoring to our problem

Link to implementation code: https://github.com/croningp/crystal_active_learning

In this section, we explain how each step of the algorithm is implemented. We use both the mathematical description and some intuitive visualization. All our explanation will be done using a simple 2-dimensional problem, representative of our chemical problem (Figure S12). All the practical implementation details can be looked up on the GitHub repository whose link is provided above.

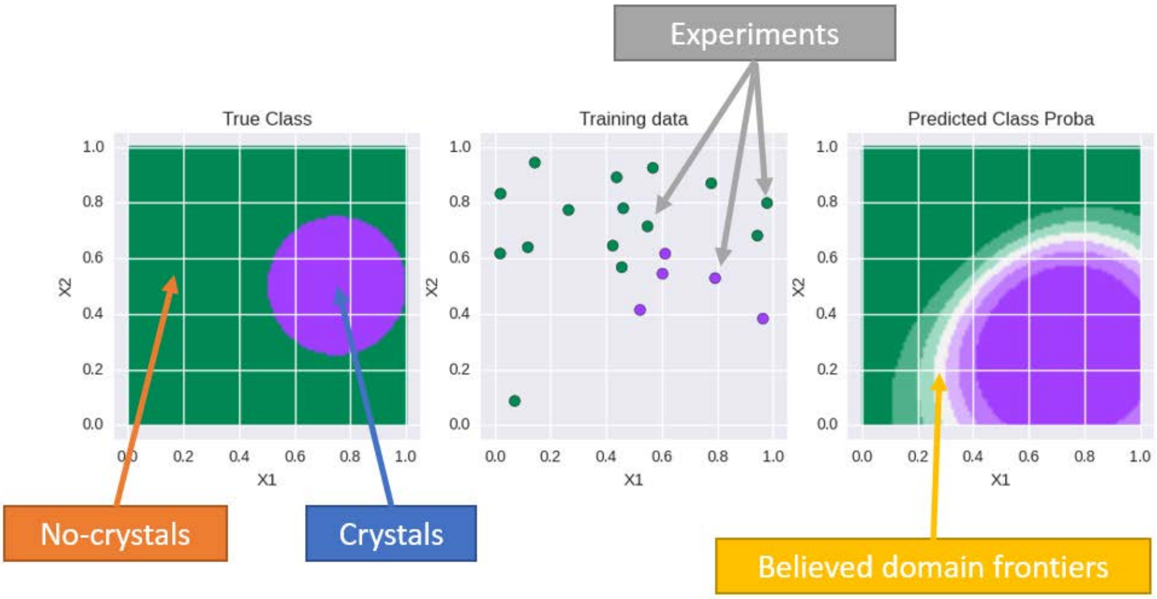

Figure S12: Simple 2D problem representative of our chemical problem, see explanation in the text.

Each of the three squares represents our experimental space; the x-axis (X1) represents for example the quantity of one of our reagents. Respectively, the y-axis represents the quantity of reagent X2. The colors green and purple represent the output of an experiment, in this example they can be of one two classes, green for no-crystal, and purple for crystal. [X1, X2] and green/purple are respectively parameters (input) and observations (output) of our system, for this example we simply created a simulated chemical problem, a thought experiment to guide our

reader during the description of our algorithm. The plot on the left of Figure S12 represents the true mapping between inputs (X1,X2) and outputs (green/purple), that is for example that an experiment with X1=0.2 and X2=0.2 (let us write this as [0.2,0.2]) produces no crystal (i.e. is in the green area). Similarly an experiment of [0.8, 0.5] is in the crystallization zone (i.e. in the purple circle).

But that very nice map on the left is unknown to us as experimenters, it is how the world works, how our system behaves. Our only source of information comes from individual experiments we perform. Those experiments are our only window in to the real mapping between inputs (X1, X2) and outputs (crystal/no crystal). The middle graph shows a few experiments performed at random, the position of each point in space represents the experimental parameters, i.e. X1 and X2, while their colors represent the output of the experiment, i.e. crystal/no crystal.

Those points are experiments that represent the initial data we start from, that is step (A) on Figure S11. Our goal now is to decide on which experiment to do next in order to improve our understanding of the world. Here, we want to select new points, new [X1, X2] pairs, and query their colours/properties. That is, we want to select new experimental parameters and observe if crystallization has happened. But we want to this with one aim in mind: to identify the boundary between the green and the purple area, that is to build a model of under which conditions our system forms crystal or not.

The first step is to model that boundary, to try to infer from the data we have what the crystallization area looks like, i.e. what is its shape in the parameter space. This is visualized in the right graph of Figure S12 and is the step B of our algorithm protocol in Figure S11. This weird looking shape is what is learned by a classification algorithm from the data collected and shown in the middle graph of Figure S12. This approximate model has been learned using a Support Vector Machine (SVM) classifier[7].

Given this model, we need to select new experiments to perform to improve its quality. As explained before, we will use an active learning strategy called uncertainty sampling. The principle is very simple, visually we want to query the point at the boundary between what we believe gives crystals and what we believe gives no crystal. Such point lie in a zone where our model does not really know what to expect, hence by performing the experiment on the real system we alleviate this uncertainty, which in turns improves our model of the system. Those steps are really simple conceptually and represent steps C to G of our algorithm. In practice, implementing them on the computer requires some tricks and tips that we will explain in detail next, but first let us look at what happens if we query 10 more experiments using our uncertainty sampling method (Figure S13).

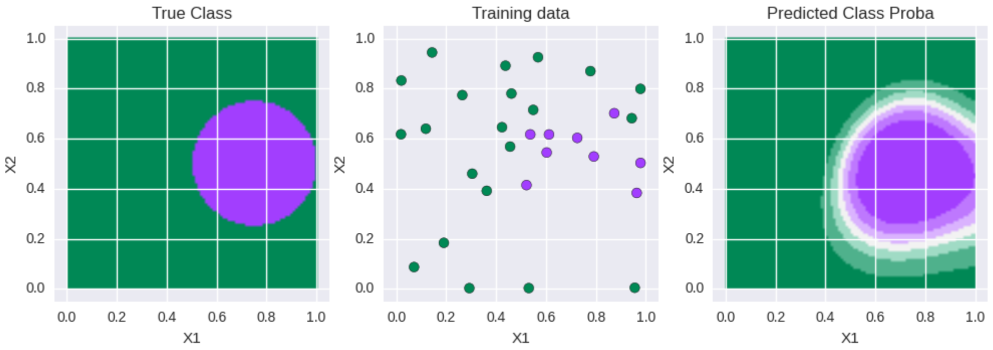

Figure S13: New model after sampling 10 new experiments selected by the uncertainty sampling algorithm were performed.

On Figure S13, and compared with Figure S12, we can see how new experiments on the middle plot enhanced our current understanding of the crystallization area (right plot). What happens now if we follow this process until we have queried 100 new experiments ?

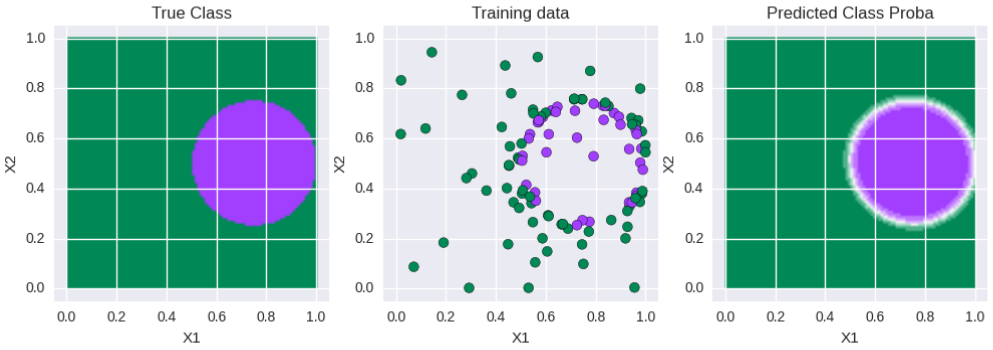

Figure S14: Model after 100 experiments selected by the uncertainty sampling algorithm were performed.

Figure S14 shows just that. It is really interesting to see how most of the experiments are performed at the boundary between the green and purple area, the algorithm somehow understood that this area is of particular interest to understand and better model the boundary between crystal and no crystal. As a result, our model (right) is now an extremely good approximation of our real system (left), and this despite most of the experiment performed being located in a small region of the chemical space.

Finally, an important question will be to compare the performance of such active learning strategy with a control strategy, which for example could be selecting random experiments to cover the chemical space uniformly. On Figure S15 we can see how the distribution of experiments performed (middle) is a lot less structured and targeted than when using the uncertainty based algorithm. As a result, the model (right) is much less accurate. Random is the baseline we used in this work to

compare our algorithm, and we also interestingly compared with how human experimenters would select experiments.

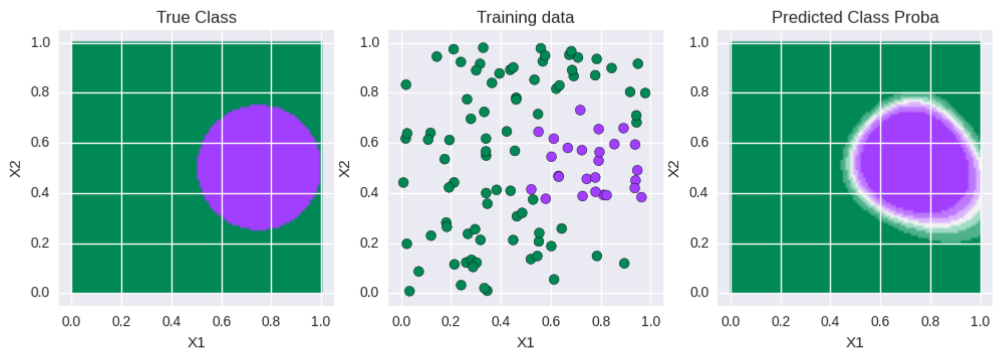

Figure S15: Results after 100 randomly sampled experiments.

Let's now explain in more depth the technicalities of implementing the uncertainty sampling.

6.2.1 Uncertainty sampling

In this subsection, we explain steps B to H of the algorithm protocol of Figure S11. As above, we will rely on simple visuals of our 2D simulated examples, along with some mathematical notation. Details of the practical implementation are available at:

https://github.com/croningp/crystal_active_learning

Each data point in our dataset can be represented as two entities, one is the experimental parameters, and the other is the label/class associated with the output of the experiment (e.g. crystal/no crystal). We note as x a vector representing the experimental parameters, e.g. x = [X1, X2] = [0.2, 0.2]. We note as y an integer representing the label/class/output of the experiments, e.g. $y = 1$ for crystal or $y = 0$ for no crystals.

The aim of step B is to train a classifier based on a database of x and y. In essence, we try to learn a function f that maps x into y with the best possible accuracy, we try to learn f in $y = f(x)$ based on examples. When y is a discrete variable, i.e. label/class, this process is called classification. There are many classification algorithms available. We decided to use the Support Vector Machine (SVM) classifier with a Radial Basis function kernel[7]. We chose it for its ability to capture non-linearity and because it has been shown to perform well in a variety of tasks[7]. It is important to understand that other classification algorithms exist and could replace the SVM classifier we use here depending on the properties of the targeted problem. Given a dataset of (x, y) examples, and each time we recomputed a new classifier, we used 10-fold cross-validation to search the best C and γ

parameters. C is the regularization parameter and γ is the kernel coefficient of the radial basis function. We ran a cross-validation with all possible combination of C and γ within np.logspace(-5, 5, 21), that is [10^{-5}, $10^{-4.5}$, 10^{-4}, …, $10^{4.5}$, 10^{5}] and selected the C and γ values producing the smallest classification error, that is the most accurate model. We used the implementation provided in scikit-learn[9], a machine learning library in Python. In the repository, the corresponding function is train_classifier in utils/classifier.py.

At this stage, we have trained a generalized model of our system based on our dataset. We now need to estimate the uncertainty of this model, this implies to have access to a probabilistic prediction from our classifier. Probabilistic prediction for SVM has been developed by J. Platt[8]. A classifier that is uncertain of an experiment x would predict the probability of each label as equal, i.e. $p(y = 0|x) = p(y = 1|x) = 0.5$. The output of such a probabilistic classifier is a vector $p_y = [0.5, 0.5]$, where the first element is $p(y = 0|x)$ and the second is $p(y = 1|x)$. Reversely, an experiment whose result is predicted with extreme confidence would lead to a prediction of $p_y = [1.0, 0.0]$. A sensible way to measure the uncertainty of a particular experiment is to compute the Shannon entropy[10] of the classifier prediction. That is $H = -\sum_i p_i \log_b p_i$ with $i \in [crystal, no\ crystal]$ and p_i the probability of x to be of class i, that is $p(y = i\,|x)$. This is implemented by the compute_normalized_entropy function in utils/tools.py.

Equipped with a method to measure the entropy of the prediction, that is the uncertainty of our model, we can now generate a lot of potential experiments and measure their uncertainty. Step C of Figure S11 consists of sampling randomly and in a uniform way N potential experiments, i.e. N vectors x. We then use the learned classifier from step B, produce probabilistic class prediction (step D) and transform such prediction into an uncertainty measure using Shannon entropy. We end up with a list of experiments associated with their uncertainty given our current model of the system (our classifier). Those steps are illustrated in Figure S16, where subplot A is the dataset of the experiments whose outcome is known, and subplot B are all the generated experiments colored by uncertainty value. Interestingly, the uncertainty area, i.e. the reddest points, lies at the boundary between the green and the purple experiments.

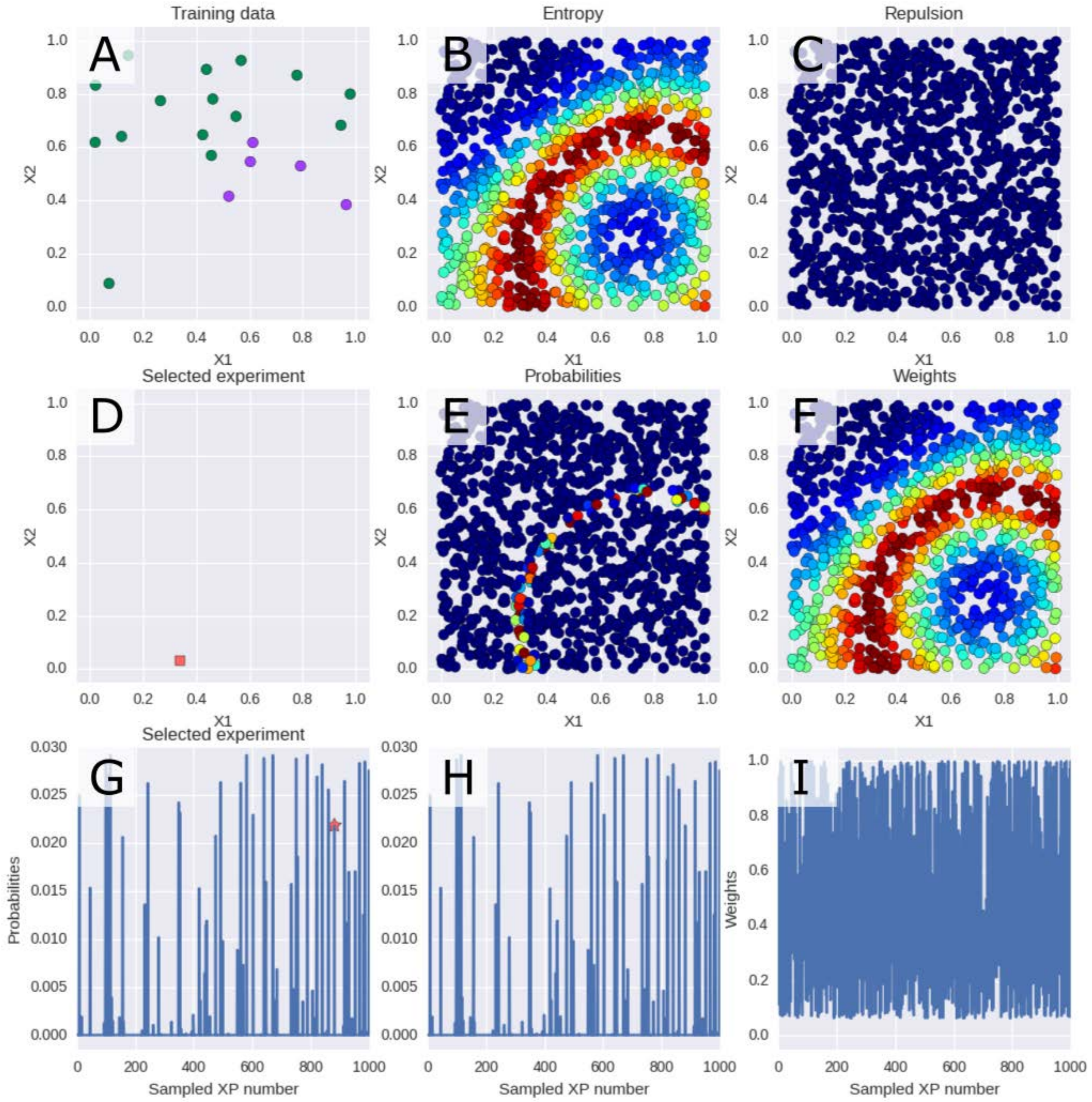

Figure S16: Visualization of the algorithmic steps for uncertainty sampling. See detailed explanation in the text.

Given this map of possible experiments associated to their uncertainty (subplot B), we have to select the next experiment to be performed on the system, which is step E on Figure S11. While the most intuitive method would be to select the experiment with the highest uncertainty, a more flexible and robust method is to probabilistically sample the next experiment with a probability proportional to its associated uncertainty. The use of probabilistic techniques has become the dominant paradigm for algorithm design in many real word applications, were noise and uncertainty are paramount[11].

Thus, an important step is to define the probability distribution over the possible experimental set based on their respective uncertainty values. A simple

association where each experiment is given as weight its uncertainty value would not be efficient, the distribution would be too flat/uniform. Instead, we use the soft-max function to convert our uncertainty values into sampling probabilities, which is a well-established method in machine learning[11]. This function is $P(w_i) = \frac{e^{w_i/\tau}}{\sum_{j=0}^{M} e^{w_j/\tau}}$ where w is the vector of all uncertainty values, i represents the ith element in that vector, $P(w_i)$ is the resulting probability associated to that element, and τ is a temperature parameter. We note that this temperature is an internal parameter of our algorithm and it is by no means linked to the chemical experiment performed. Intuitively, the soft-max function will simply normalize any vector to sum to 1 with an exponential weighting on each element. The temperature parameter (τ) is used to bias the weighting towards a more uniform (for high value of τ) or a more skewed distribution (for low value of τ).

Therefore, an important step is to select a good value for τ, too high and the distribution will be flat, meaning we will not explore the boundary; too low and the distribution will be too 'spiky' and we lose the advantage of probabilistic sampling. And often a unique τ might not be optimal all along an experiment because the distribution of uncertainty will evolve as we know more about our system. Hence, we used a self-tuning τ mechanism that consists of defining an aim on some properties of the probabilistic distribution and find among many possible τ the one matching best our objective.

We defined our objective in such a way that the top 5% of the experiments should be assigned 95% of the probability. In other words, that we have 95 percent of chance to sample a point within the 5 percent best points ranked by uncertainty value. We then scan for the best τ within a large list of 100 different τ values, which in code was defined as np.logspace(-5, 1, 100). This process is implemented as the compute_best_temperature function in utils/uncertainty.py.

This step of moving from uncertainty to sampling probability is illustrated on Figure S16 by the transition between subplot F and E as a map representation (colors represent the weights/probabilities). The raw weight to probability transition is also illustrated by the transition between subplot I and H.

To select the next experiment we then sample a new experiment based on this probability distribution. This is illustrated by the transition from subplot E to D and from subplot H to G on Figure S16 and implemented in the probabilistic_choice function in utils/tools.py.

The selected experiment is then executed on the system, for this SI this is our simple 2D circle world but in the work presented in this paper this is by running a crystallization experiment on the robotic platform. This is step F and G of Figure S11. And the process is repeated all over again. The entire procedure is implemented in the generate_next_samples function in utils/uncertainty.py. Figure S17 shows the

samples collected (A) and the uncertainty maps (B) after 50 iterations is our 2D circle problem. We note that the points and the uncertainty are grouped at the boundary between the two classes.

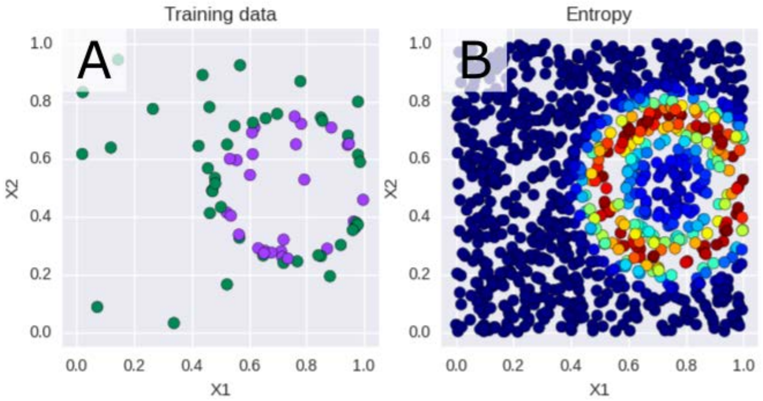

Figure S17: Visualization for the algorithmic steps after 50 iterations of uncertainty sampling. See detailed explanation in the text.

At this point, we understand how uncertainty sampling works and is implemented in practice. An important aspect is that new experiments are selected one by one, and should be tested on the system before being able to select a new experiment. This is not a problem for computer based experiments, e.g. simulated systems, but might become quickly problematic for real world experiments. Indeed, in our case, a crystallization experiment needs hours to crystallize and as a result if we were to use the algorithm as described, it would take us 100 days to run 100 experiments using this method, something that, needless to say, is not acceptable. In the next subsection, we present how the algorithm can be modified to select next experiments in batch while ensuring each batch is not composed of extremely similar experiments.

6.2.2 Modification for batch sampling

Our platform allows to run about 10 crystallization experiments per day, allowing for the night to crystallize our product. As such, we wanted to sample experiments in a batch of 10 from the algorithm. The problem is that the distribution from which we sample would not be updated in the meantime, meaning that all 10 experiments (or 20, 30 depending on the application) would be sampled from the same distribution. Hence, given the nature of the classification algorithm, the samples in the batch would likely be made of similar experiments.

To avoid this, we implemented a ‘rejection field’ around points already sampled in the current batch. Conceptually, this rejection field simply updates the sampling distribution of new experiments based on the experiments sampled before, so that

sampling a new point around already sampled points becomes less probable. We provide technical and practical details next.

In a batch sampling, the first experiment sampled uses the exact protocol and implementation as described in the previous section. The subsequent experiments will be sampled by reusing the same entropy/uncertainty map as derived from the classifier trained on the available data, but superimposed with a repulsion map dependent on the previous experiments sampled in the current batch.

To build the repulsion map we reuse the principles of the SVM algorithm and use the RBF kernel to define a repulsion function dependent on the position of each previously sampled point. The RBF kernel is described as $K(x, x') = \exp(-\gamma\, ||x - x'||^2)$ where $||x - x'||^2$ is the squared Euclidean distance and γ the kernel parameter. γ is chosen to be 10 times larger than the γ value selected during the cross validation procedure of the SVM classifier. Intuitively, it means the repulsion `radius` is smaller than the characteristic `radius` of the classifier. The logic behind this is to build a finer/local repulsion zone than the entropy map, which depends on the SVM classifier, allows. This is implemented in the compute_normalized_repulsion function in utils/uncertainty.py.

In practise, to combine the uncertainty and the repulsion map on equal grounds and in a scalable manner, we ensure that both are bounded between 0 and 1. To do so, we simply normalize the entropy by the maximum of the entropy observed, and the repulsion map by its maximum. The final weights for the sampling process are computed as: $weights = entropy * (1 - repulsion)$, so that the entropy is conserved when far away from repulsion area, and reduced in proximity of a repulsion area. This is implemented in the compute_weights function in utils/uncertainty.py.

We illustrate this process with in Figure S18 for our 2D circle world simulation, which represents the sampling process for the first experiment of a batch sampling process. As this is the first experiment in the batch, the repulsion map (subplot C) is uniform/ not in use and the sampling of the new point (subplot D) depends only on the entropy maps (subplot B).

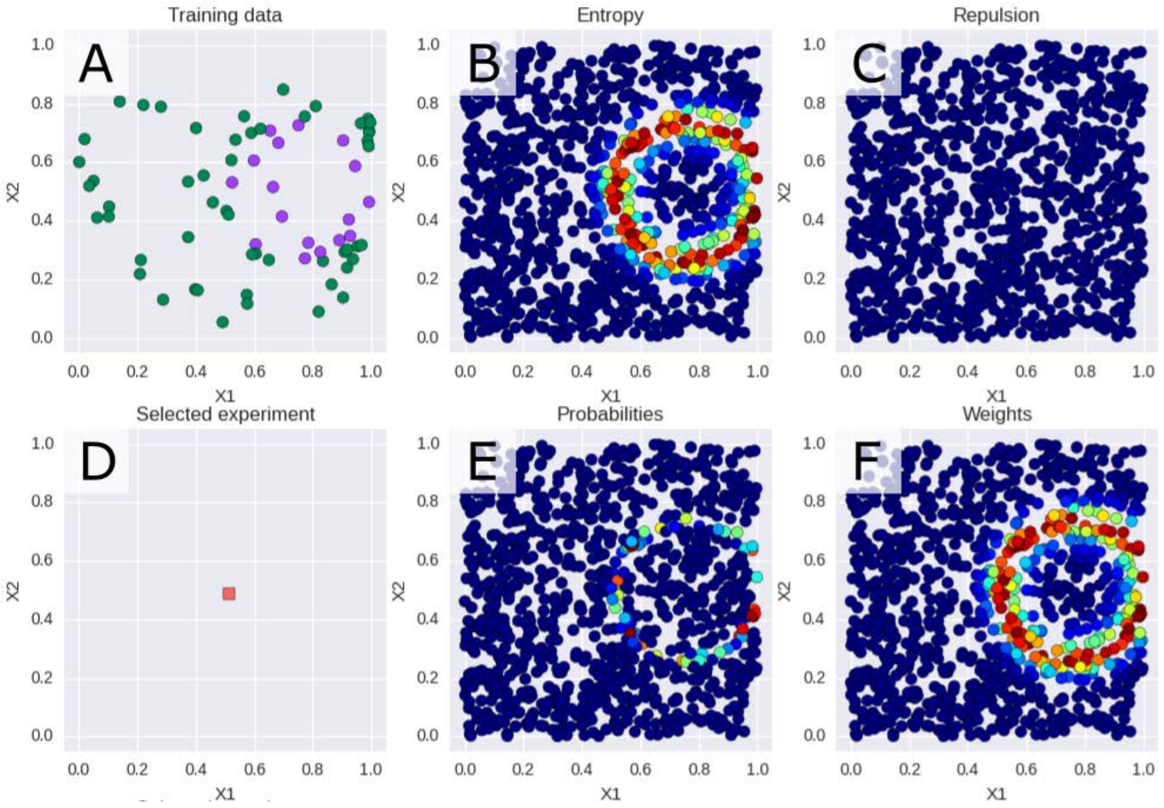

Figure S18: First point sampled in the batch version of our algorithm. The repulsion map (C) is not used and the sampling of the new point (D) depends only on the entropy maps (B).

For sampling the second experiment, and as illustrated in the box (C) of Figure S19, the first experiment now creates a repulsion zone around itself. The entropy map (subplot B) is unchanged (although the position of the hypothetical experiments sampled/displayed is different). The repulsion map (subplot C) is now active with a repulsion area around the previously sampled experiment/point. The sampling distribution (subplot F) is thus biased away from the previously sampled point, clearly marked by the 'hole' in the 'uncertainty ring'. The new sampled experiment is shown is (subplot D, red square).

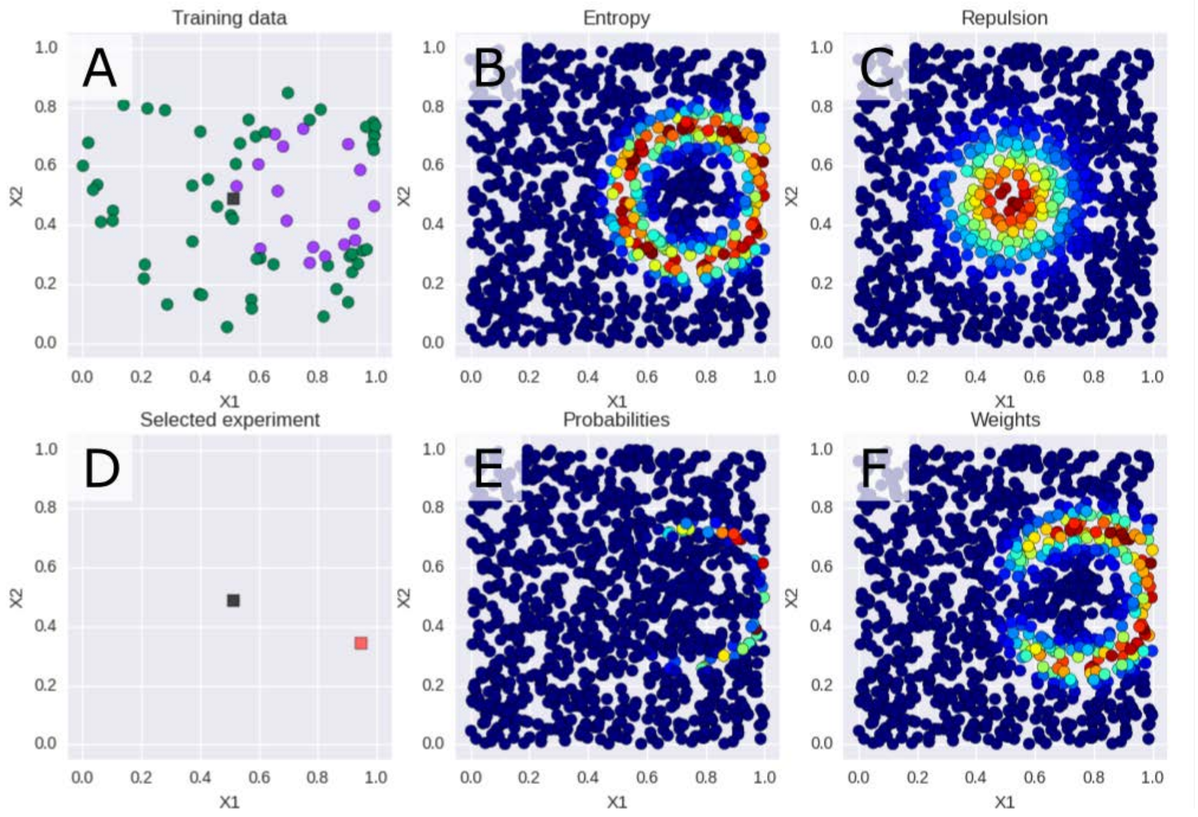

Figure S19: Second point sampled in the batch version of our algorithm. The entropy map (B) is unchanged. The repulsion map (C) is now active with a repulsion area around the previously sampled point. The sampling distribution (F) is thus biased away from the previously sampled point, clearly marked by the 'hole' in the 'uncertainty ring'. The new sampled point is shown is (D).

The process is repeated again with each new experiment sampled adding a repulsion zone. Figure S20 shows the sampling patterns for the 10th sample in the batch. The box (subplot D) shows how the batch points (all the squares red and grey) are sampled all around the uncertainty zone (subplot B), allowing to cover more uniformly the uncertainty zone despite the batch information requirements.

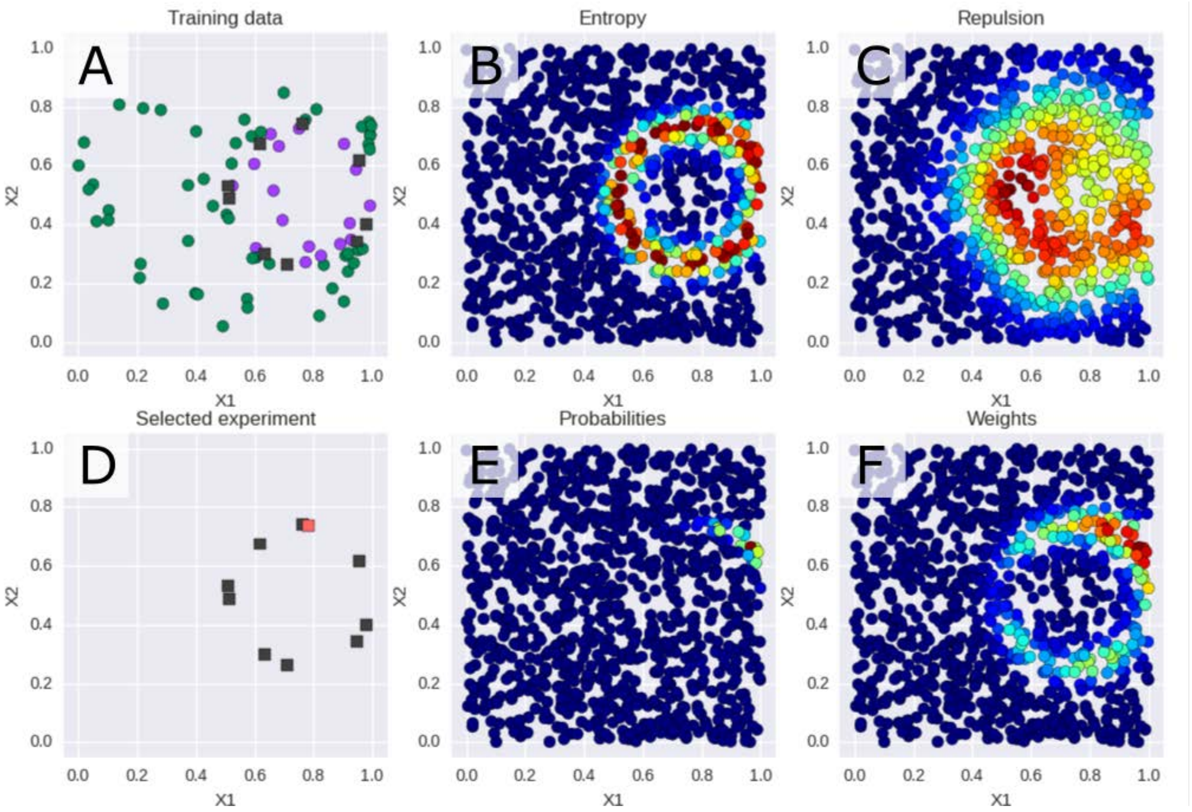

Figure S20: Sampling patterns for the 10th sample in the batch.

6.2.3 Performances

We now compare in simulation the performances of each version of the algorithm, namely:

- Uncertainty single: is the original version where each experiment queried is tested immediately and added to the dataset.
- Uncertainty batch: is the modified algorithm, where experiments are requested in batch of 10, then executed and added to the dataset.
- Uncertainty batch – no repulsion: is the straightforward version of the batch algorithm, where experiments are requested in batch of 10 without any repulsion field.

We also compare these versions to a random sampling strategy, which simply queries random experimental parameters, without any feedback from the data collected.

All results are averaged over 100 simulations of our algorithm with each time a different starting dataset. The code to reproduce our results is available under the simulation folder of the associated repository.

Figure S21 shows that our batch version of the uncertainty sampling performs similarly to the original one by one uncertainty sampling. All uncertainty sampling methods outperform largely a random strategy. Our repulsion field based batch sampling slightly improves over a simple batch version.

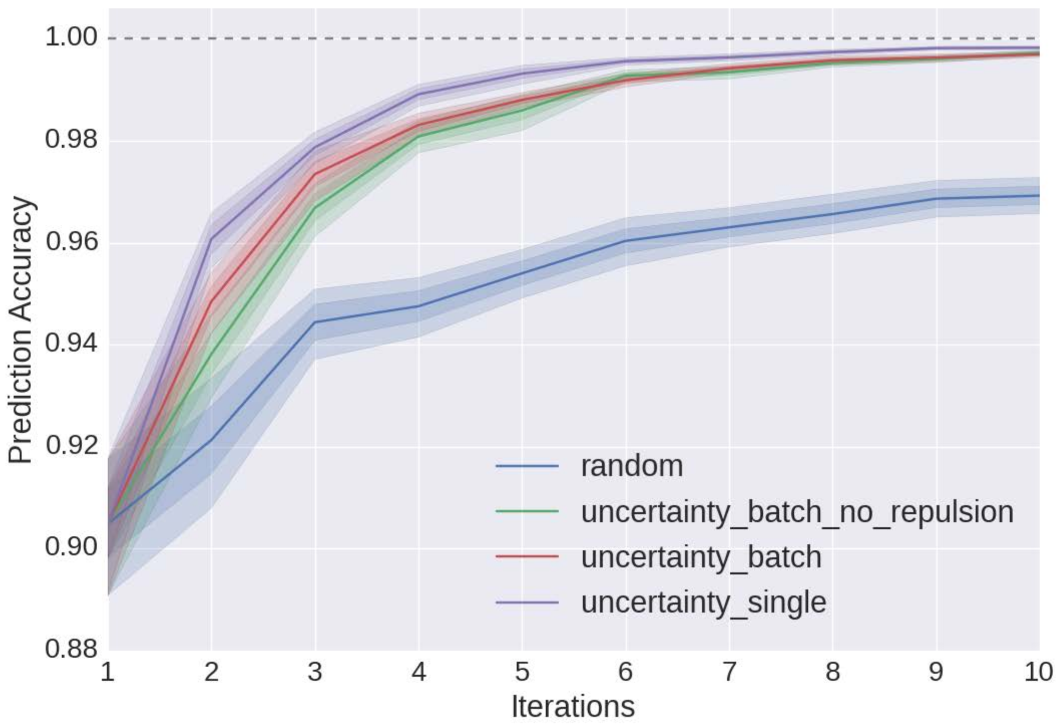

Figure S21: Evolution of the model quality through iterations for each algorithmic method (mean, 63% and 95 % confidence intervals out of 100 simulated experiments in the circle 2D world). An iteration represents 10 experiments. All uncertainty methods outperform random sampling. Our modified uncertainty sampling in batch improves slightly over the no_repulsion variant and reaches asymptotically the same performance of the one by one uncertainty sampling baseline method.

6.3 A second example

We now illustrate the same algorithmic principle to a different simple 2D problem to illustrate the flexibility of the method to different domains. This new domain considers two classes separated by a sinusoidal frontier. Figure S22 shows this domain and a typical starting dataset to explore and identify it.

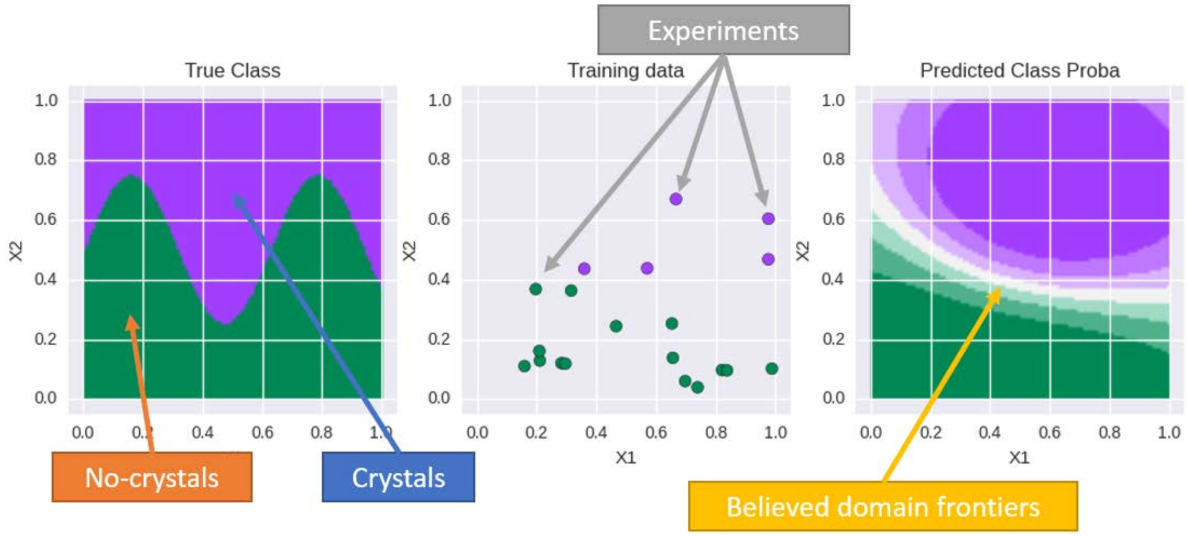

Figure S22: The sinusoidal domain

Figure S23 compares the performances of the sampling methods presented previously on this new domain and confirms the advantage of active learning methods over simpler random exploration methods.

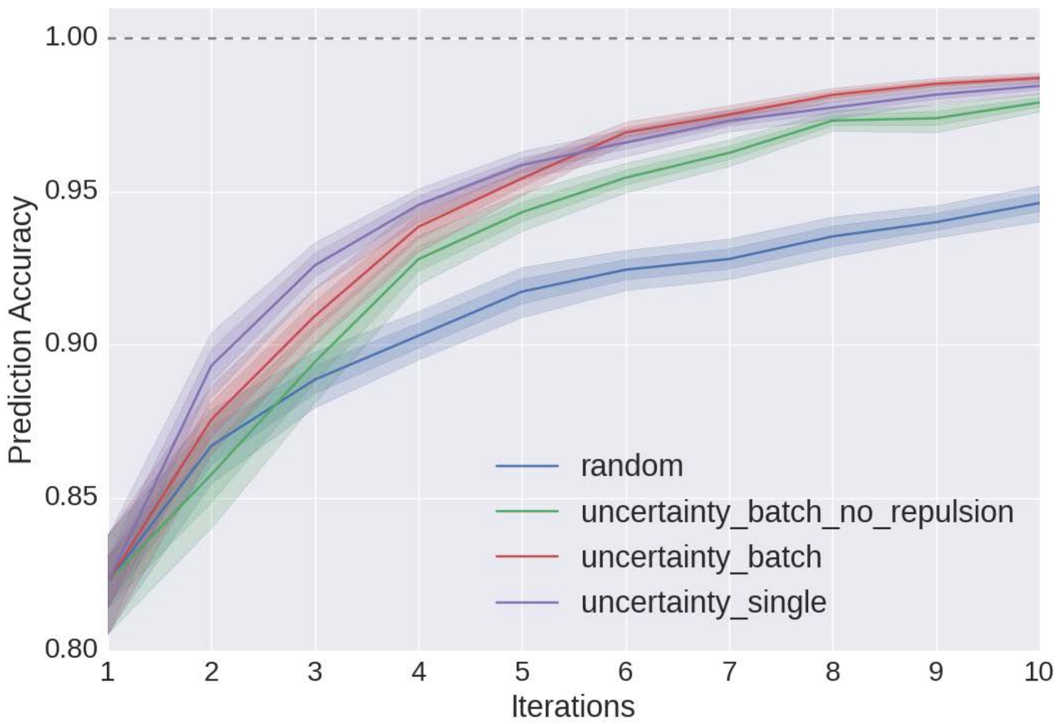

Figure S23: Evolution of the model quality through iteration for each algorithmic method (mean, 63% and 95 % confidence intervals out of 100 simulated experiment in the sinus 2D world). One iteration represents 10 experiments. All uncertainty methods outperform random sampling. Our modified uncertainty sampling in batch

improves slightly over the no_repulsion variant and reaches asymptotically the same performance of the one by one uncertainty sampling baseline method.

Finally, Figure S24 shows a typical set of requested experiments and the result from the uncertainty sampling method after 10 iterations (or 100 experiments requested), and Figure S25 shows the same for the random sampling method. It is clear how the uncertainty sampling is more directed and targeted towards identifying the boundary between the two domains.

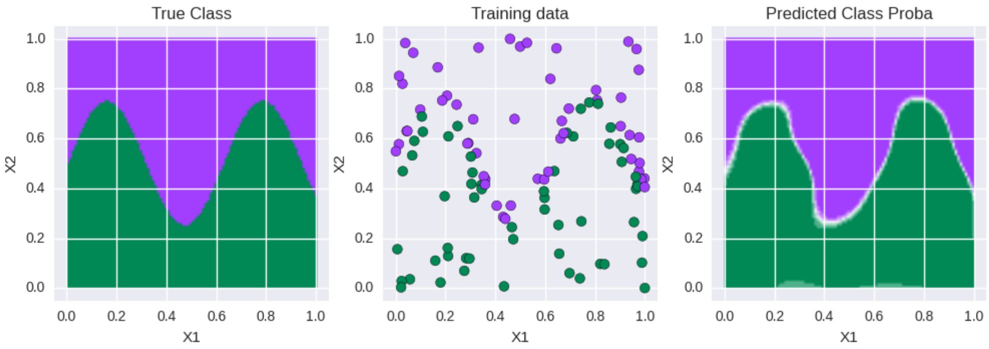

Figure S24: Sampling and model after 100 points using the uncertainty sampling algorithm

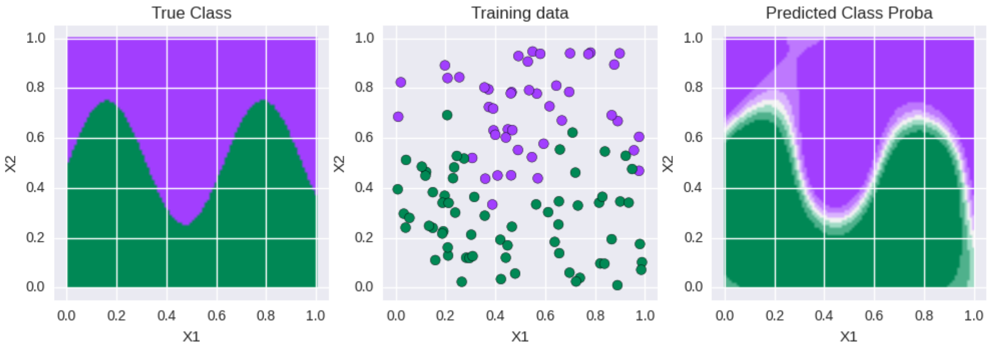

Figure S25: Results after 100 randomly sampled experiments.

6.4 Limitations and Discussions

The active learning algorithm and the specific uncertainty sampling implementation presented here are powerful tools for reducing the number of experiments needed to identify and characterize the boundary of various systems. A few limitations need to be considered.

First, the algorithm will not make a difference between stochastic areas and uncertain areas, that is if the explored system is intrinsically stochastic, leading 50% of the time to crystal, and 50% to no crystal, and if this is modelled properly by the

underlying model, then the uncertainty sampling will consider this area of high interest because the entropy will be maximal. This can be annoying as the focus of the algorithm will be directed towards an area that is known to be stochastic, thus not providing more information to our model.

Another limitation is that the algorithm will not look away from its currently known domain cluster. For example, if there are two different and non-connected clusters in the chemical space leading to crystals, the algorithm will not actively seek and discover them. Uncertainty sampling actually reduces the chances of doing so because it will focus its search on the boundaries of the already known cluster.

7. Initial set of data

As explained above an initial set of data need to be provided to bootstrap the search. The initial set of data (Figure S26) was obtained in two stages: a random search and a local search. The random search was used to gather data covering a wide range of experimental conditions (Table S3). Two experiments out of the 46 performed lead to crystallization. Single crystal X-ray diffraction analysis confirmed that the main product is cluster (**1**). We then conducted 43 more experiments in the vicinity of the 2 successful experiments, all of which crystallized. As a result, the initial set of data consists of 89 points which was provided to each method as the starting information for their exploration.

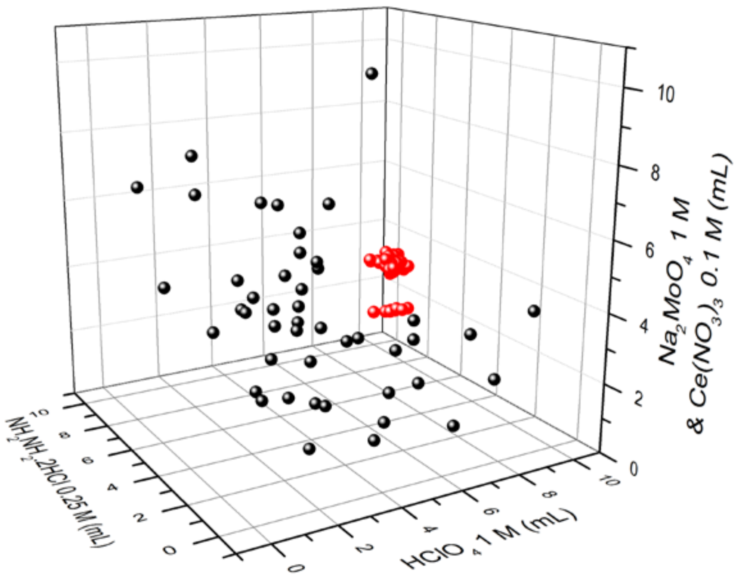

Figure S26: 3D graph of the initial set of data. Coloring code: crystals, red; non-crystals, black

Table S3: Initial set of data.

H_2O [ml]	$HClO_4$ [ml]	$NH_2NH_2.2HCl$ [ml]	$Ce(NO_3)_3.6H_2O/Na_2MoO_4.2H_2O$[a] [ml]	Crystals[b]
3.583	3.658	5.913	1.846	0
0.176	4.382	3.653	6.789	0
4.65	2.382	2.621	5.347	0
2.349	3.924	4.441	4.286	0
2.244	9.303	1.983	1.47	0
4.231	3.209	3.359	4.201	0
8.177	3.487	3.214	0.122	0
3.647	4.739	0.777	5.837	1
5.329	5.701	2.344	1.626	0
0.759	2.737	4.715	6.789	0
0.719	3.515	0.055	10.711	0
1.31	9.385	0.374	3.931	0
3.185	4.714	5.327	1.775	0
2.835	5.308	1.447	5.41	1
1.339	5.337	5.759	2.565	0
5.356	6.081	3.173	0.39	0
4.898	3.161	6.03	0.911	0
1.19	8.411	4.788	0.611	0
4.995	2.403	3.356	4.246	0
5.697	4.196	4.097	1.01	0
4.606	7.952	2.176	0.266	0
2.428	0.658	4.681	7.233	0
3.27	3.855	4.561	3.314	0
0.102	8.135	4.711	2.052	0
0.548	1.646	9.445	3.361	0
7.78	4.837	1.766	0.617	0
6.193	4.182	3.479	1.147	0
1.318	2.87	3.942	6.87	0
0.579	0.037	7.406	6.978	0
4.498	4.52	2.819	3.163	0
4.552	2.08	4.473	3.895	0
0.589	4.792	4.997	4.623	0
5.16	4.667	0.467	4.707	1
6.399	2.708	4.726	1.166	0
4.771	4.561	2.262	3.407	0
4.375	2.299	1.618	6.707	0
0.015	1.113	5.87	8.002	0
0.605	3.659	7.957	2.779	0
2.098	6.769	2.706	3.427	0
2.961	3.152	3.163	5.724	0

5.205	2.542	3.553	3.7	0
3.973	3.615	4.196	3.216	0
3.853	2.361	4.528	4.258	0
3.461	2.005	4.828	4.706	0
0.971	4.509	4.576	4.944	0
4.401	5.659	1.969	2.971	0
7.892	0.233	2.74	4.135	0
2.405	7.939	1.46	3.196	0
4.862	3.969	5.38	0.789	0
3.647	4.739	0.777	5.837	1
3.614	4.894	0.634	5.857	1
3.648	4.696	0.77	5.885	1
3.678	4.554	0.903	5.865	1
3.677	4.597	0.909	5.817	1
3.708	4.413	1.034	5.845	1
3.707	4.455	1.04	5.797	1
3.738	4.271	1.166	5.825	1
3.739	4.228	1.16	5.873	1
3.645	4.731	0.53	6.093	1
3.589	4.855	0.75	5.806	1
3.674	4.676	0.606	6.043	1
3.647	4.72	0.95	5.683	1
3.928	4.64	0.615	5.817	1
3.932	4.62	0.789	5.659	1
4.212	4.541	0.456	5.791	1
3.774	4.591	0.924	5.711	1
3.702	4.539	0.841	5.918	1
3.572	4.687	0.691	6.05	1
3.627	4.488	0.757	6.128	1
3.642	4.674	0.772	5.913	1
3.635	4.581	0.764	6.019	1
3.638	4.628	0.768	5.966	1
3.641	4.797	0.975	5.586	1
3.697	4.705	0.809	5.789	1
3.619	4.799	0.911	5.672	1
3.614	4.854	1.101	5.431	1
2.891	5.248	1.548	5.313	1
2.861	5.423	1.288	5.428	1
2.804	5.49	1.177	5.53	1
2.776	5.374	1.337	5.513	1
5.119	4.89	0.247	4.744	1
5.063	4.631	0.693	4.613	1
5.021	4.859	0.47	4.65	1
4.964	4.603	0.914	4.52	1

5.005	4.37	1.145	4.48	1
5.017	4.742	0.631	4.609	1
4.919	4.712	0.853	4.516	1
5.026	4.652	0.734	4.588	1
4.973	4.682	0.793	4.552	1

[a]: It represents the sum of two equal volumes.

[b]: Absence of crystal is assigned the value zero (0). Presence of crystal is assigned the value (1).

As seen on Table S3, when suggesting the new set of experiments, all methods should provide us with a list of four volume parameters corresponding to the addition of five reagents in the manner of H_2O, $HClO_4$ 1 M, $NH_2NH_2{\cdot}2HCl$ 0.25 M and $Na_2MoO_4{\cdot}2H_2O$ 1 M /$Ce(NO_3)_3{\cdot}6H_2O$ 0.1 M. Because of the 1:1 volume ratio, the latter is expressed as the sum of two equal volumes.

8. Analysis of experiments performed between methods

Throughout this section, a coloring scheme is applied for all experimental data representing the categories of their respective crystallization events as follows:

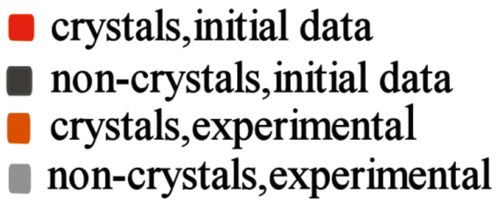

8.1 Visualization of crystallization methods

In Figure S27 we illustrate in a simplified 2D representation our qualitative observations about the strategies used by the human experimenters and the algorithm. We can observe a more polar search of the algorithm (bottom, left) and a more directional exploration from the human experimenters (bottom, right).

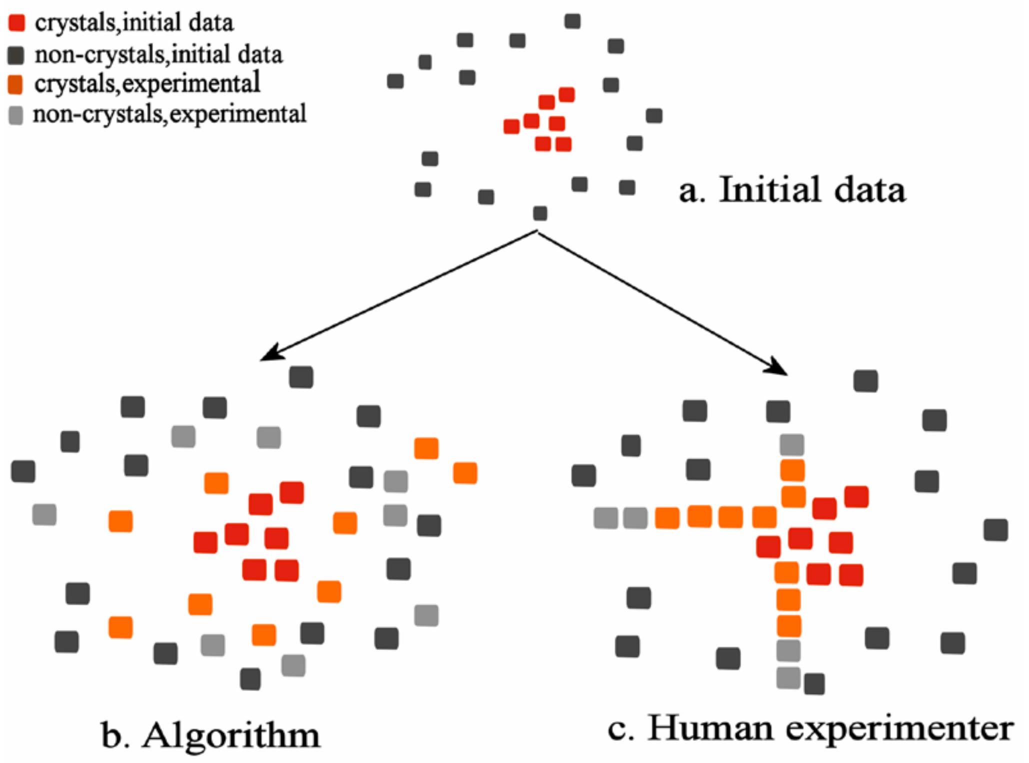

Figure S27: 2D conceptual scheme of the exploration.

In Figure S28 we can see the actual data from our experiments in a 3D representation. Figure S28 plots the experiments performed in 3D graphs having as axes: first, the volume of $HClO_4$ (in mL); second, the volume of $NH_2NH_2.2HCl$ (in mL) and third, the combined volume of $Na_2MoO_4.2H_2O$ and $Ce(NO_3)_3.6H_2O$ (in mL). We can observe the similarities with our illustration in Figure S27.

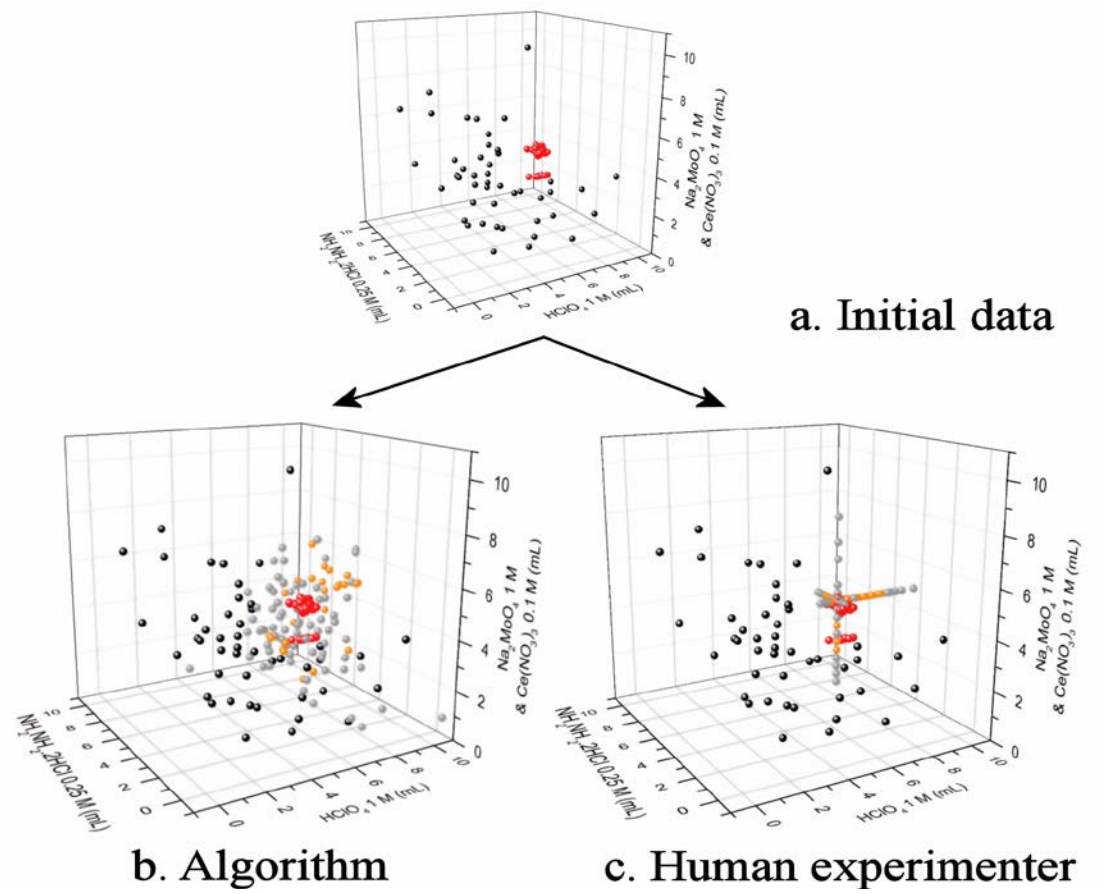

Figure S28: 3D conceptual scheme of the exploration

8.2 Experimental protocol as developed by the human experimenters

In this study, the human experimenters were aware of the chemical formula of compound (**1**), the reagents, the reaction conditions (temperature and reaction time), the platform and the initial set of data. They were not aware of the overall aim of comparing strategies among procedures. Each human experimenter was instructed to develop their own strategy given the objective to identify the range of experimental conditions where compound (**1**) can be isolated.

<u>Human experimenter 1</u>

Human experimenter 1 initially used six 2D graphs to visualize the initial set of data for both the crystallizing and the non-crystallizing mixtures for the following ratios: $Na_2MoO_4.2H_2O/Ce(NO_3)_3.6H_2O$ over $HClO_4$, $Na_2MoO_4.2H_2O/Ce(NO_3)_3.6H_2O$ over $NH_2NH_2.2HCl$ and $NH_2NH_2.2HCl$ over $HClO_4$.

a. $Na_2MoO_4.2H_2O/Ce(NO_3)_3.6H_2O$ over $HClO_4$

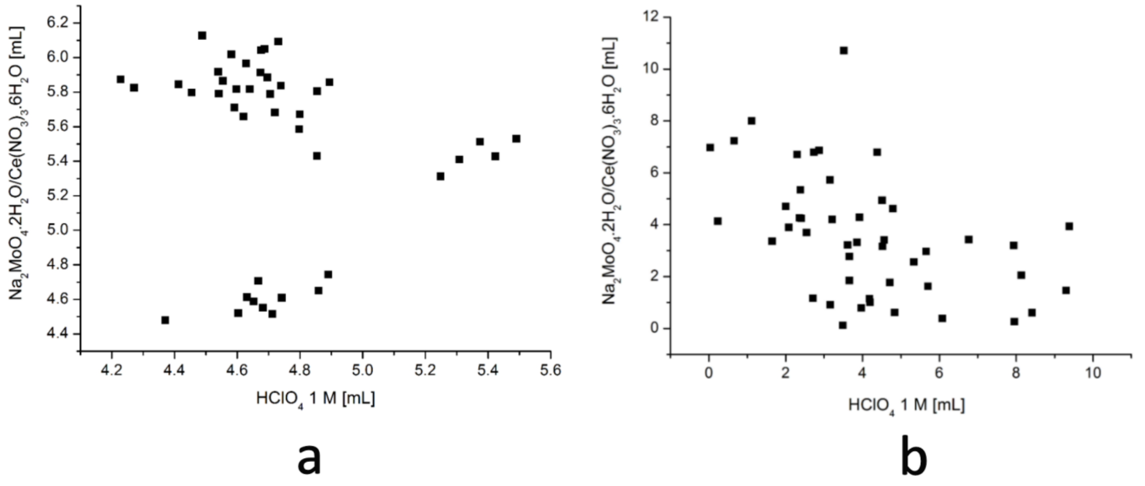

Figure S29: 2D representation of a) the crystallizing mixture and b) the non-crystallizing mixture.

b. $Na_2MoO_4.2H_2O/Ce(NO_3)_3.6H_2O$ over $NH_2NH_2.2HCl$

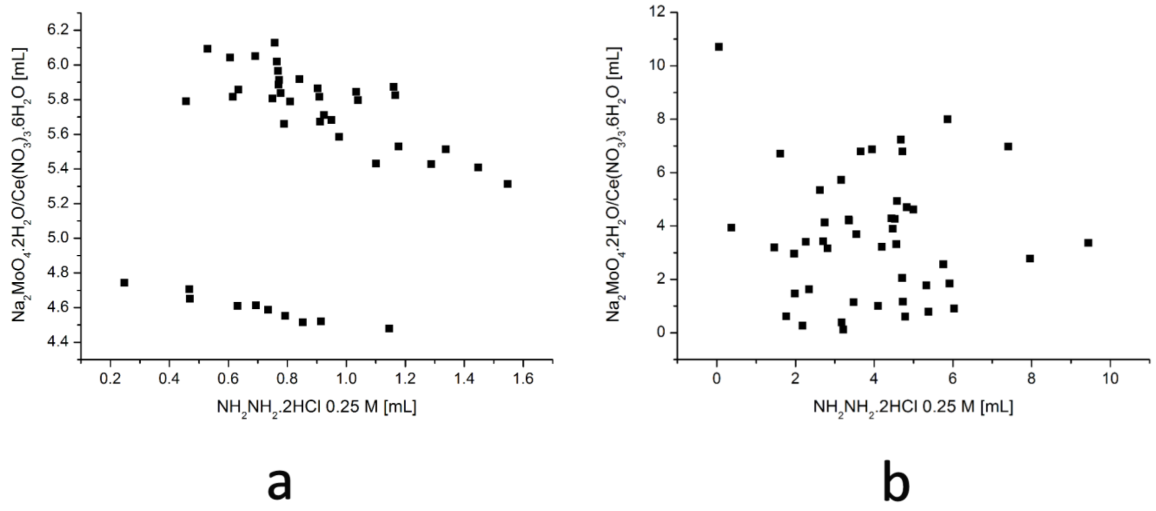

Figure S30: 2D representation of a) the crystallizing mixture and b) the non-crystallizing mixture.

c. $NH_2NH_2.2HCl$ over $HClO_4$

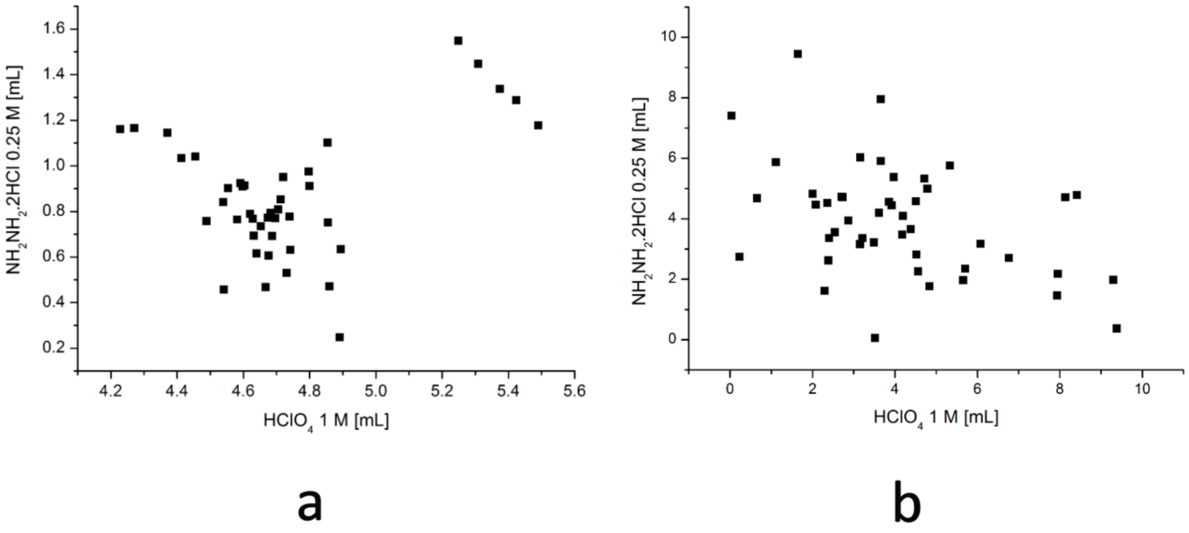

Figure S31: 2D representation of a) the crystallizing mixture and b) the non-crystallizing mixture.

Human experimenter 1 observed that the crystallizing reactions are clustered in regions of reaction space very closely (Figures S29a, S30a, S31a) whilst the non-crystallizing are broadly spread out around these regions (Figures S29b, S30b, S31b). From each graph separately, human experimenter 1 estimated the values of reagent volume roughly 10% removed outwards from the crystallizing regions in order to determine the next 10 reactions: four reactions for Figure S29, four reactions for Figure S30 and two for Figure S31 since this ratio seemed to be the least important, which was further confirmed through the experiments and it was later completely abandoned. After each series of ten reactions the additional data was added to the original plot of the initial data set.

The graphs produced for the non-crystallizing reactions were only used as a control in order to avoid replication of a previously given reaction. That is because the volume of the third reagent was plotted in these graphs, which volume was not represented on the 2D graphs.

For the volume of the third reagent an average of the successful values for each reagent from all of the crystal yielding reactions was used. The volumes of the reagents from the successful data have a relatively narrow window so this was deemed adequate by the human experimenter 1.

<u>Human experimenter 2</u>

Human experimenter 2 used Table S3 to initially determine the rough boundaries of the concentration of reagents used for the initial data. The findings are summarized below:

Reagents	Boundaries [M]

Molybdenum/reducing agent	6.86 – 38
Molybdenum	0.15 – 0.36
Acid	0.28 – 0.36
Acid/Molybdenum	1.43 – 2.08

Subsequently, human experimenter 2 generated a plan in which the three variables to be explored were the acid ($HClO_4$ 1M), the Mo and Ce content ($Na_2MoO_4.2H_2O$ 1M/$Ce(NO_3)_3.6H_2O$ 0.1M) and the reducing agent ($NH_2NH_2.2HCl$ 0.25 M). Two of these variables were kept constant at any time and the third was allowed to change. In general, the ratios for the experiments were calculated following this plan:

	1st day	2nd day	3rd day	4th day	5th day	6th day	7th day	8th day	9th day	10th day
Mo/reducing agent [M]	5.5-45	7.5-24	6.7-24.9	15	15	15	15	15	15	15
Mo [M]	0.2	0.2	0.2	0.2	0.2	0.2	0.1-0.3	0.11-0.21	0.2	0.18
Acid [M]	0.3	0.3	0.28-0.4	0.30-0.345	0.285-0.385	0.27-0.45	0.48-0.54	0.46-0.47	0.3-0.465	0.3
Acid/Mo [M]	1.5	1.5	1.4-2.0	1.5-1.725	1.425-1.925	1.95-2.25	1.11-3	1.4-2.3	1.525-2.325	0.5

8.3 Results

In this section we have the comparison between the two runs for all three methods. The results shown are after the end of the 100 experiments mark requested in the beginning of our study.

1. Human experimenters

General overview

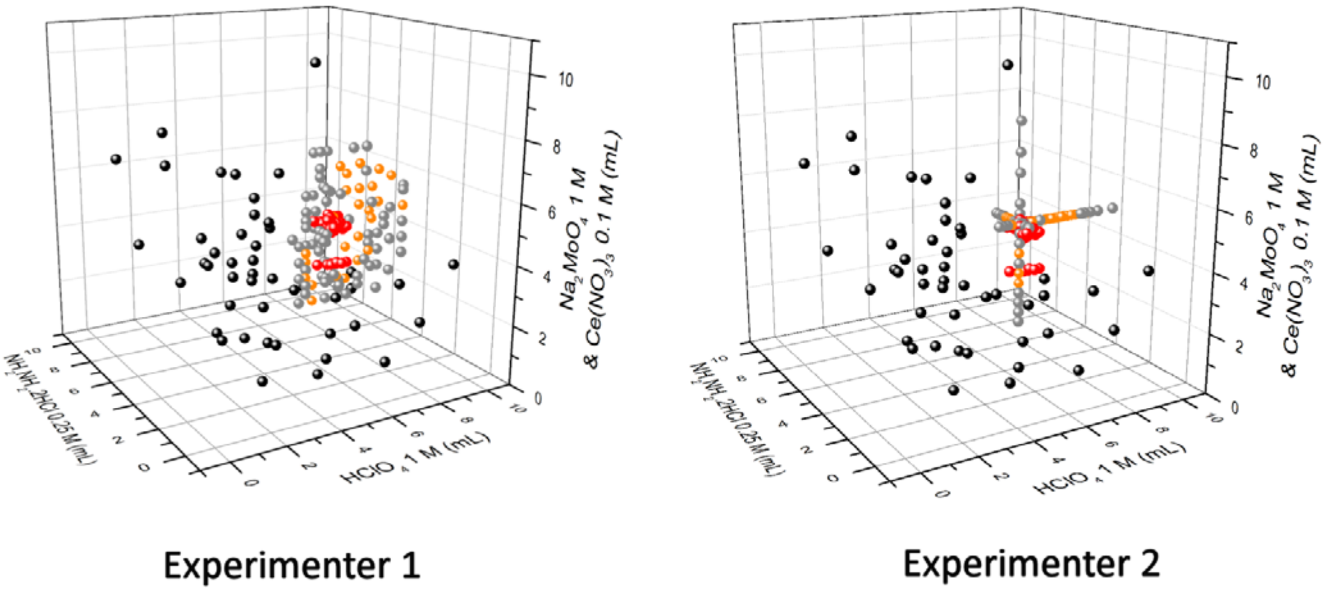

Figure S32: 3D graph of the data from experimenter 1 (left) and experimenter 2 (right).

View along the acid and reducing agent plane

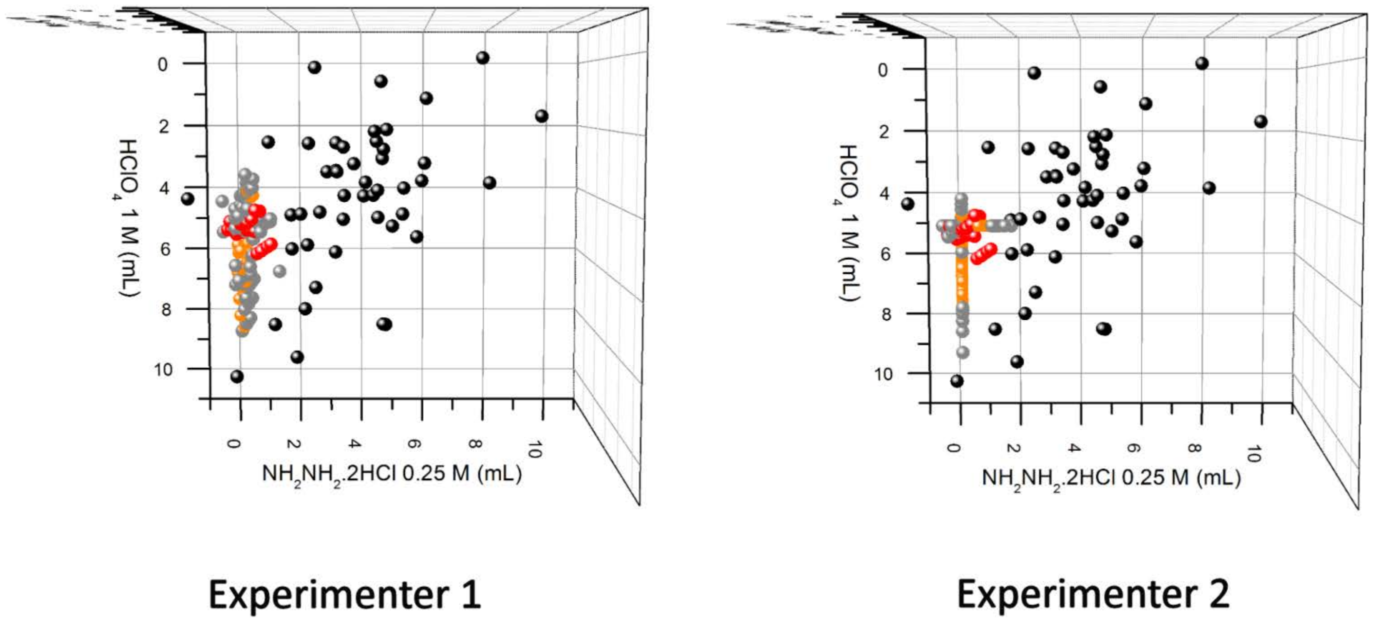

Figure S33: 3D graph of the data from experimenter 1 (left) and experimenter 2 (right).

View from the acid perspective

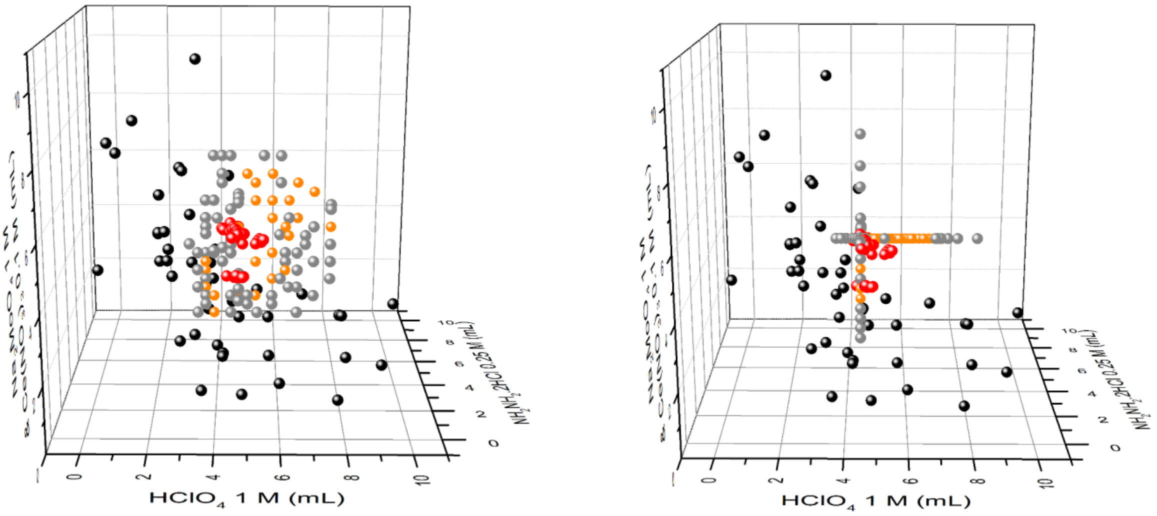

Experimenter 1 **Experimenter 2**

Figure S34: 3D graph of the data from experimenter 1 (left) and experimenter 2 (right).

View from the reducing agent perspective

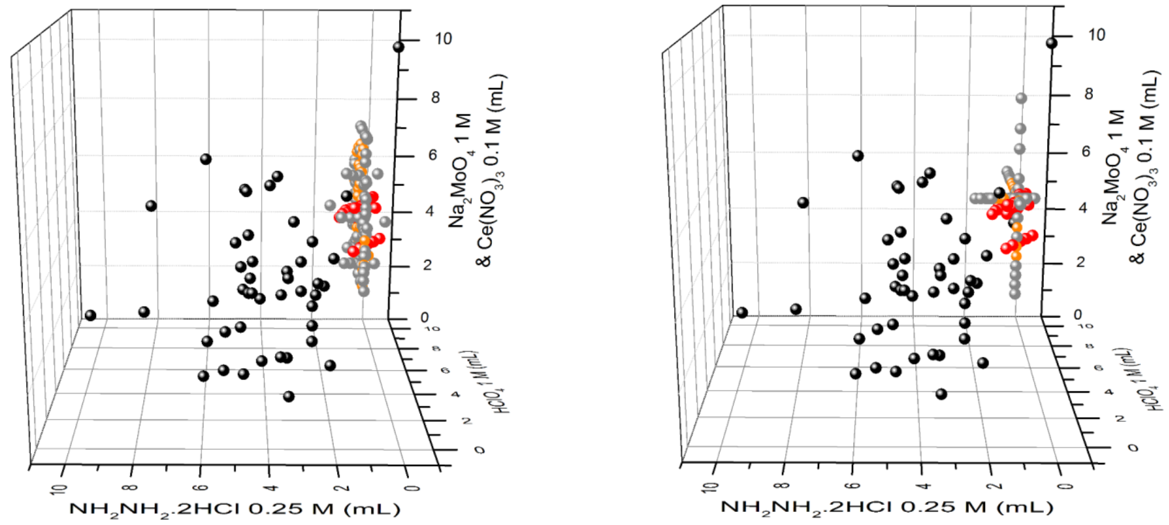

Experimenter 1 **Experimenter 2**

Figure S35: 3D graph of the data from experimenter 1 (left) and experimenter 2 (right).

2. Random search

General overview

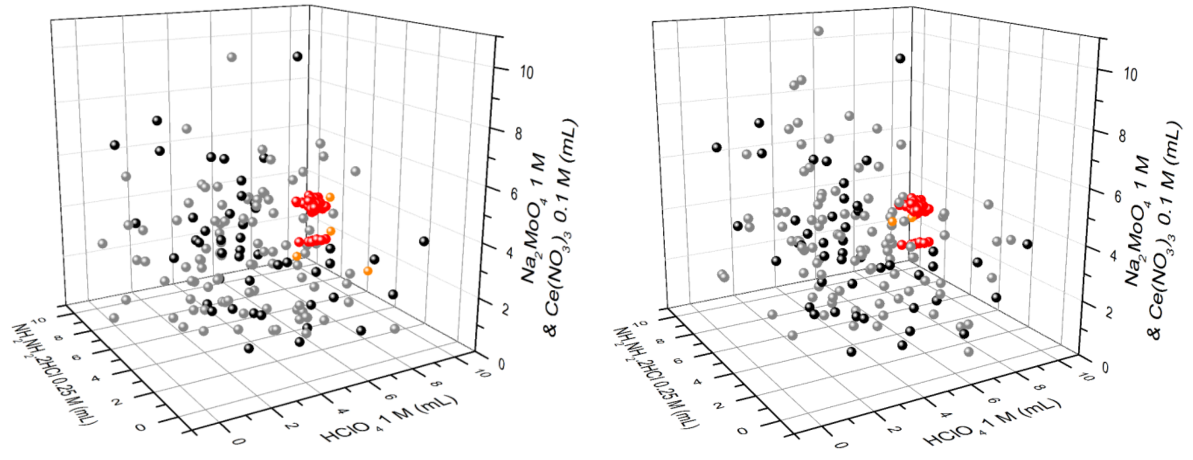

Random 1 Random 2

Figure S36: 3D graph of the data from random 1 (left) and random 2 (right).

View along the acid and reducing agent plane

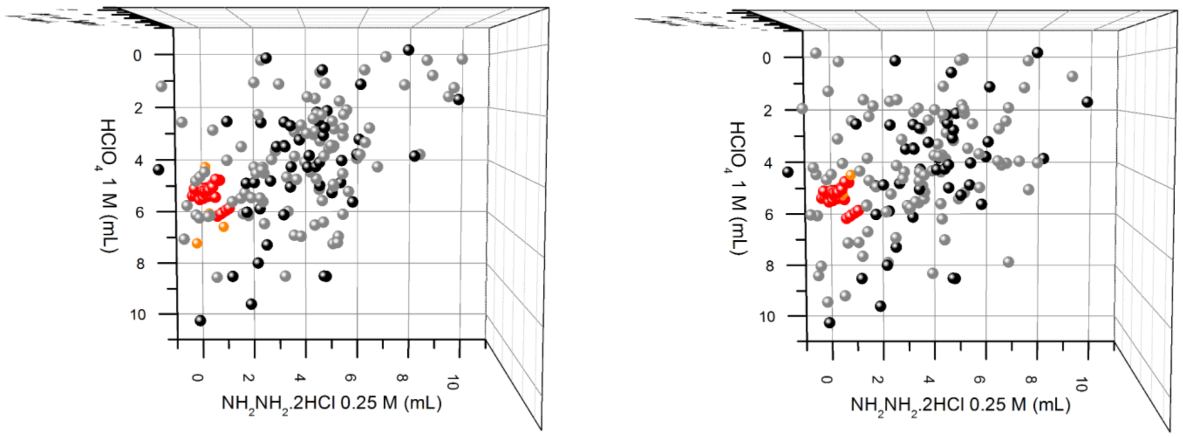

Random 1 Random 2

Figure S37: 3D graph of the data from random 1 (left) and random 2 (right).

View from the acid perspective

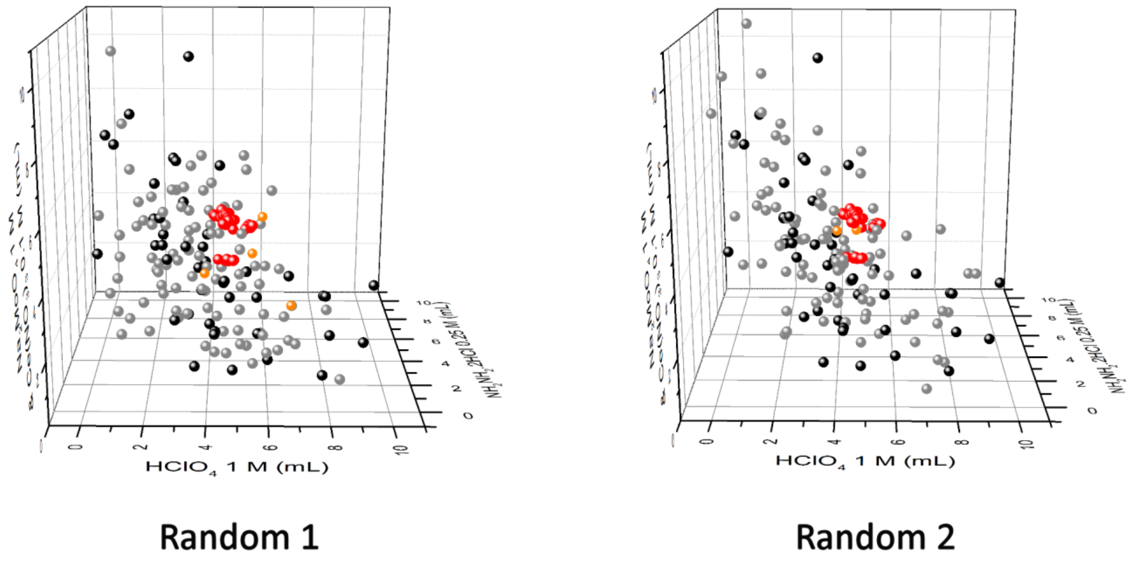

Figure S38: 3D graph of the data from random 1 (left) and random 2 (right).

View from the reducing agent perspective

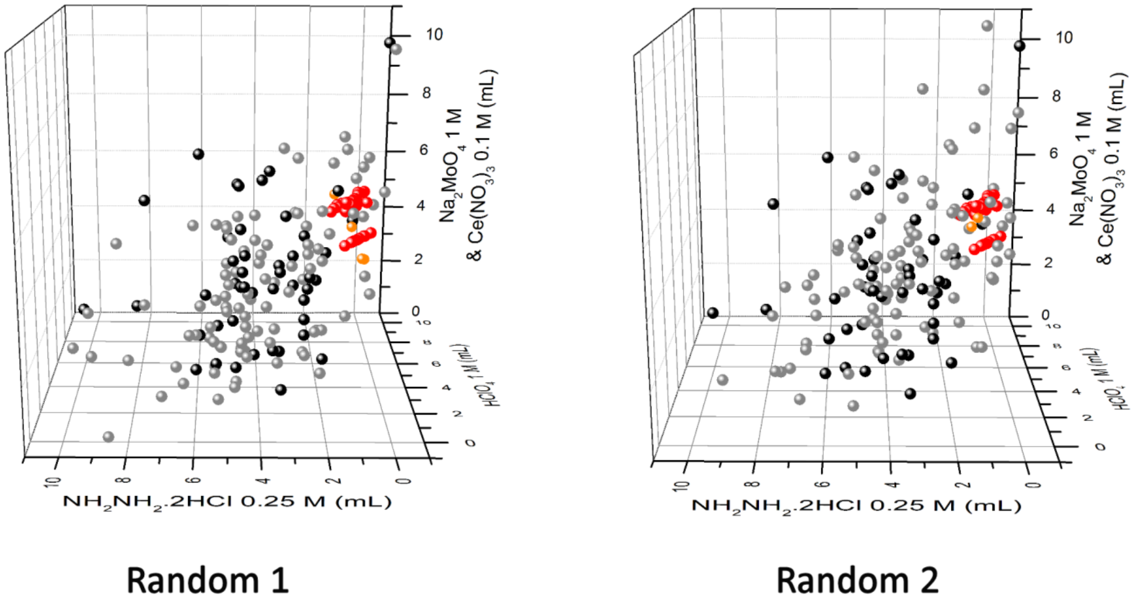

Figure S39: 3D graph of the data from random 1 (left) and random 2 (right).

3. Algorithm

General overview

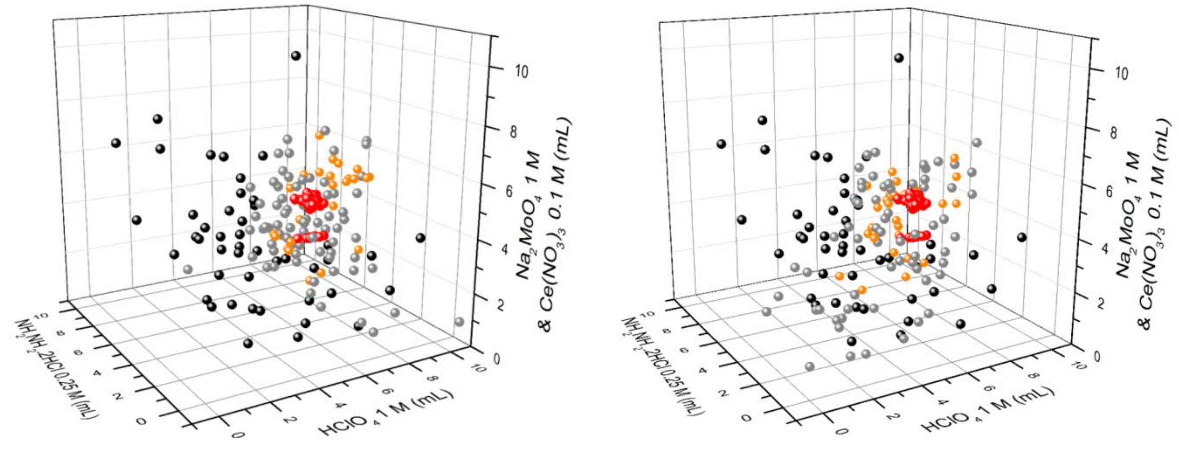

Run 1 Run 2

Figure S40: 3D graph of the data from algorithm run 1 (left) and algorithm run 2 (right).

View along the acid and reducing agent plane

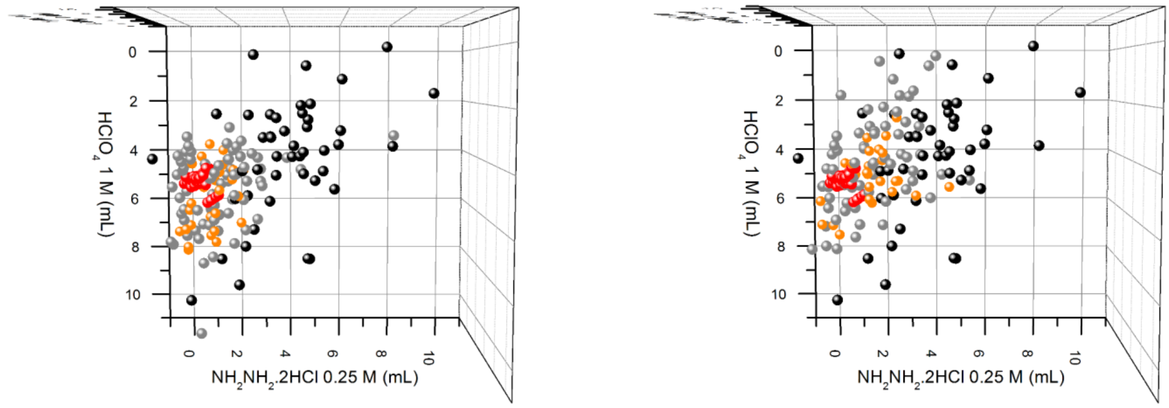

Run 1 Run 2

Figure S41: 3D graph of the data from algorithm run 1 (left) and algorithm run 2 (right).

View from the acid perspective

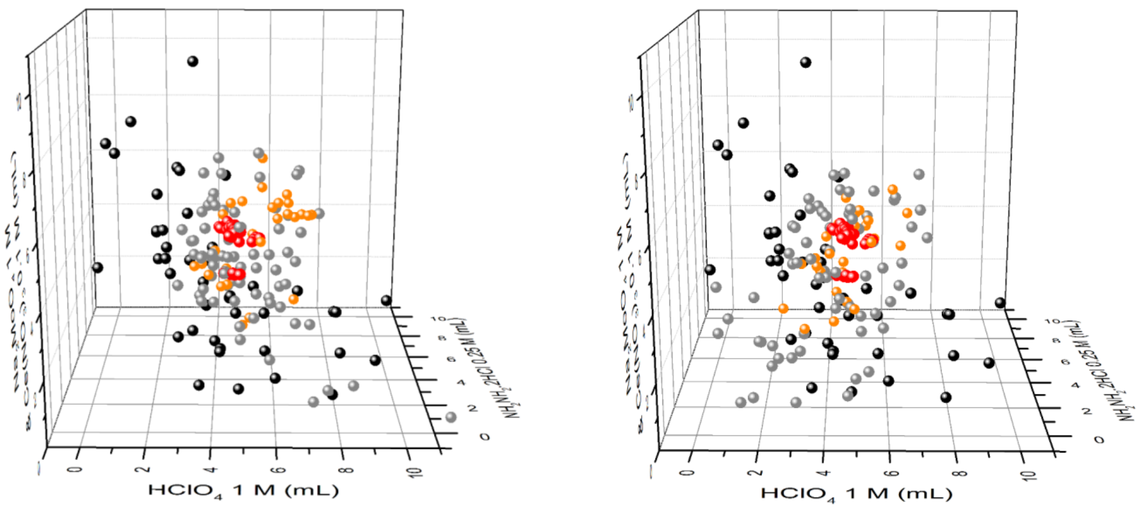

Run 1

Run 2

Figure S42: 3D graph of the data from algorithm run 1 (left) and algorithm run 2 (right).

View from the reducing agent perspective

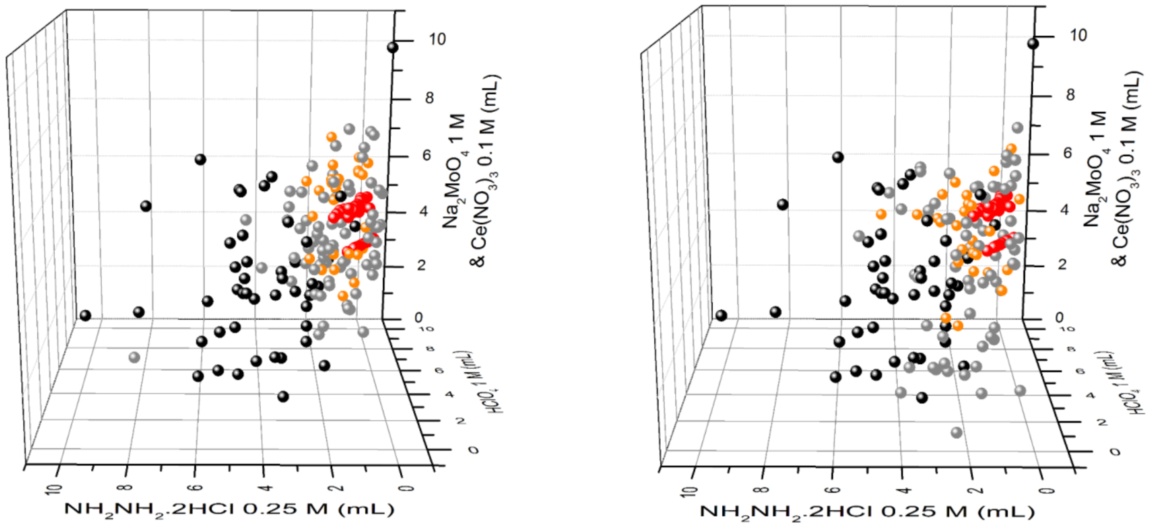

Run 1

Run 2

Figure S43: 3D graph of the data from algorithm run 1 (left) and algorithm run 2 (right).

9. Single-crystal X-ray diffraction validation of the products observed in the crystallization boundaries

In section 8, we were able to identify the crystallization boundaries of {$Mo_{120}Ce_6$} (**1**) based on an optical confirmation of the presence of crystals under strong light (white light emitting diode, 3300-3500 lux at a distance of 5 cm). As a next step, we wanted to characterize if by moving outwards to the boundaries of crystallization we keep obtaining compound (**1**) or if we observe the formation of new compounds. For this reason, we performed a series of 12 experiments as seen on Table S5 and depicted on Figure S44 with the aim of using single-crystal X-ray and ICP analysis.

Table S5: Experiments performed for the single-crystal X-ray and ICP analysis validation.

Sample	H_2O [ml]	$HClO_4$ [ml]	$NH_2NH_2.2HCl$ [ml]	$Ce(NO_3)_3.6H_2O/Na_2MoO_4.2H_2O$ [ml]
s1	3.237	4.5	1.263	6
s2	3.088	4.5	1.411	6
s3	3.175	5.025	0.8	6
s4	2.875	5.325	0.8	6
s5	2.575	5.625	0.8	6
s6	2.05	6.15	0.8	6
s7	1.6	6.6	0.8	6
s8	1.15	7.05	0.8	6
s9	1.373	5.878	1.36	6.389
s10	1.541	6.331	0.342	6.786
s11	0.221	6.099	2.423	6.256
s12	0.008	5.574	1.662	7.755

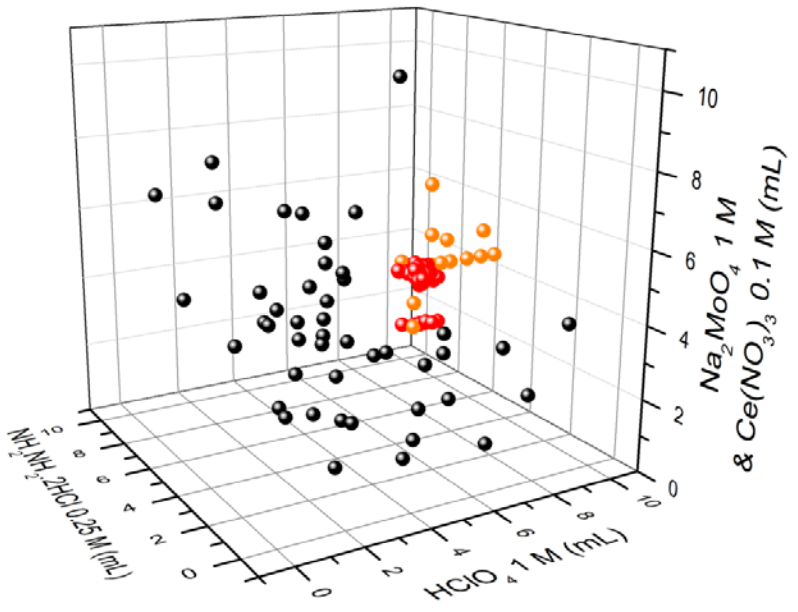

Figure S44: 3D graph of the experiments performed for the single-crystal X-ray and ICP (part 9) validation analysis. Coloring code: crystals from initial conditions, red; non-crystals from initial conditions, black; crystals for validation, orange.

Unfortunately, as seen on Table S6, the quality of the crystalline material that we obtained was low and single-crystal X-ray analysis was difficult to reliably perform.

Table S6: Single-crystal X-ray results.

Sample	Compound observed	Resolution [Å]	Number of reflections	2theta [°]
s1	{$Mo_{120}Ce_6$}	1.2	1071	34.88
s2	Weak diffraction	1.4	250	30.18
s3	Powder material	none	none	none
s4	Powder material	none	none	none
s5	Powder material	none	none	none
s6	Weak diffraction	3.5	160	11.51
s7	Unreliable unit cell measurement	2.8	353	14.88
s8	Small crystals/ Weak diffraction	none	none	none
s9	Weak diffraction	3.4	132	11.98
s10	Weak diffraction	none	none	none
s11	Powder material	none	none	none
s12	Powder material	none	none	None

Measured at 15 sec exposure time; detector distance 60 mm

For our investigation of what compounds crystallize in the boundaries we repeated the following selected reactions:

1. From the human experimenters

In this batch of experiments we observed the presence of compound (**1**) in an experiment belonging to the third area of crystallization. This area has been discovered from one of the human experimenters and from both the uncertainty algorithms.

Table S7: Single-crystal X-ray results for selected reactions from the human experimenters.

Sample	H_2O [ml]	$HClO_4$ [ml]	$NH_2NH_2.2HCl$ [ml]	$Ce(NO_3)_3.6H_2O/Na_2MoO_4.2H_2O$ [ml]	Compound observed
s1	3.891	5.75	0.859	4.5	Weak diffraction
s2	1.641	5.75	0.859	6.75	{$Mo_{120}Ce_6$}
s3	3.891	5.25	0.859	5	Small crystals/ Weak diffraction
s4	2.141	5.25	0.859	6.75	Small crystals/ Weak diffraction

s5	6.641	4	0.859	3.5	Small crystals/ Weak diffraction
s6	3.3	4.5	1.2	6	Weak diffraction
s7	1.15	7.05	0.8	6	Weak diffraction
s8	5.5	4.5	0.8	4.2	Small crystals/ Weak diffraction
s9	2.725	5.475	0.8	6	Small crystals/ Weak diffraction
s10	2.81	4.5	1.69	6	Weak diffraction

Measured at 15 sec exposure time; detector distance 60 mm

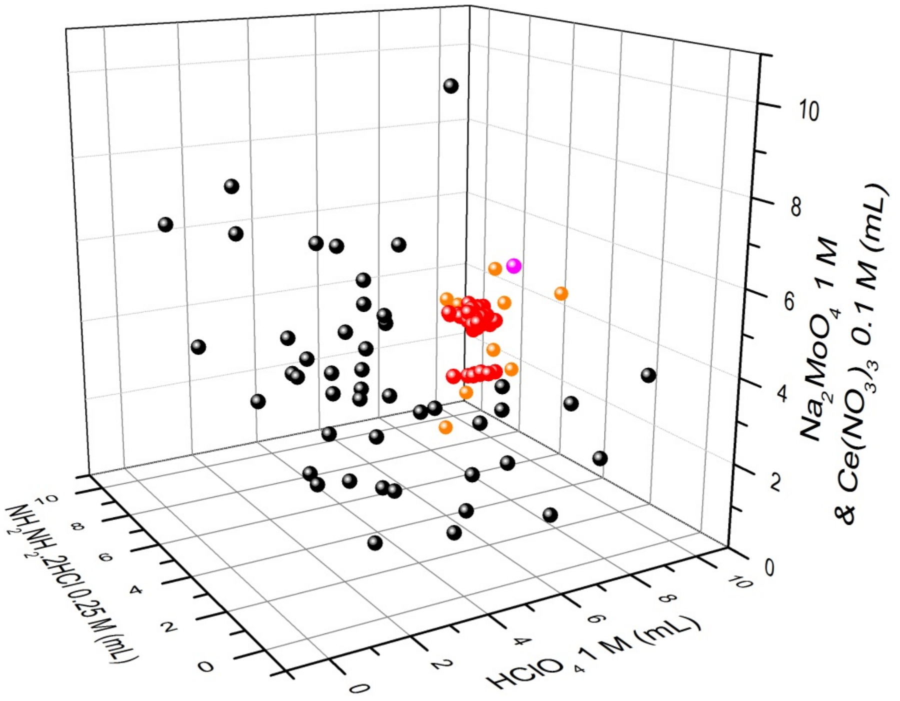

Figure S45: 3D graph of the selected experiments repeated from the human experimenters. Coloring code: crystals from initial conditions, red; non-crystals from initial conditions, black; crystals for validation, orange; crystals confirmed as {$Mo_{120}Ce_6$} by single-crystal X-ray analysis, magenta.

2. From the random search

In an experiment far away from the crystallization boundaries of compound (**1**) we observed the presence of {Mo_{154}}.

Table S8: Single-crystal X-ray results for selected reactions from the random search.

Sample	H_2O [ml]	$HClO_4$ [ml]	$NH_2NH_2.2HCl$ [ml]	$Ce(NO_3)_3.6H_2O/Na_2MoO_4.2H_2O$ [ml]	Compound observed
s1	4.706	6.733	0.213	3.348	{Mo_{154}}
s2	3.923	5.487	0.821	4.769	Microcrystalline material
s3	2.045	5.814	1.401	5.74	Small crystals/ Weak diffraction
s4	6.231	3.947	0.621	4.201	Small crystals/ Weak diffraction
s5	4.378	4.049	1.296	5.277	Powder material
s6	3.897	4.685	1.035	5.383	Powder material

Measured at 15 sec exposure time; detector distance 60 mm

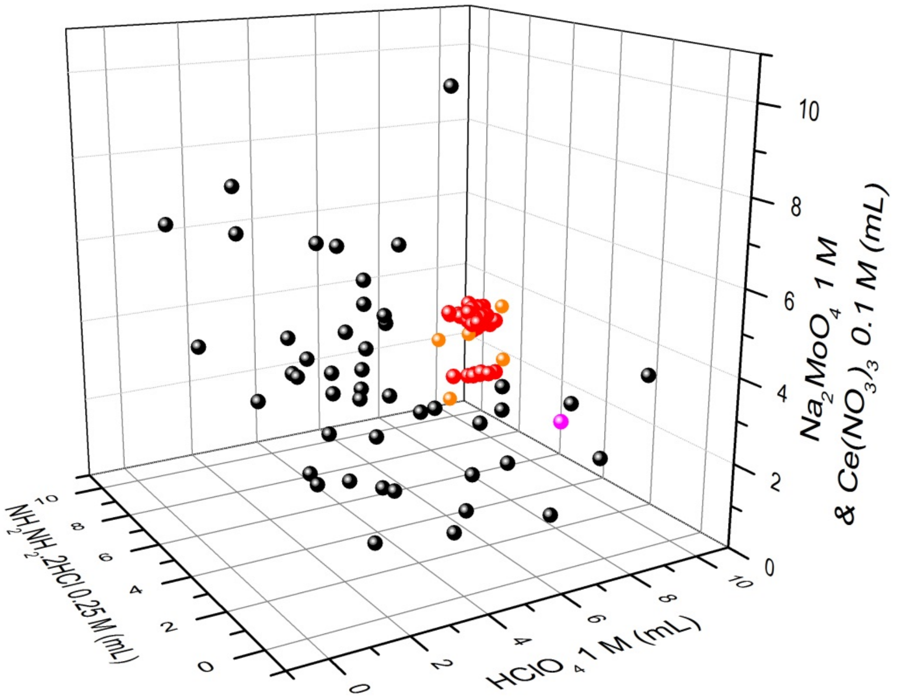

Figure S46: 3D graph of the selected experiments repeated from the random search. Coloring code: crystals from initial conditions, red; non-crystals from initial conditions, black; crystals for validation, orange; crystals confirmed as {Mo_{154}} by single-crystal X-ray analysis, magenta.

3. From the algorithm

In this batch of selected experiments we observed not only the presence of {$Mo_{120}Ce_6$} in the third region of crystallization, as mentioned before and in the main

manuscript, but also the inability of single-crystal X-ray diffraction to confirm the presence of a different product.

Table S9: Single-crystal X-ray results for selected reactions from the algorithm.

Sample	H_2O [ml]	$HClO_4$ [ml]	$NH_2NH_2.2HCl$ [ml]	$Ce(NO_3)_3.6H_2O/Na_2MoO_4.2H_2O$ [ml]	Compound observed
s1	1.373	5.878	1.36	6.389	{$Mo_{120}Ce_6$}
s2	3.954	4.438	2.369	4.239	Microcrystalline material
s3	2.905	4.345	1.649	6.101	Small crystals/ Weak diffraction
s4	0.991	7.043	0.583	6.382	Small crystals/ Weak diffraction
s5	5.211	4.279	1.572	3.937	Microcrystalline material
s6	4.582	3.672	2.021	4.724	Small crystals/ Weak diffraction
s7	5.55	4.673	1.067	3.71	Microcrystalline material
s8	1.282	6.515	0.773	6.429	Unreliable new unit cell[a]
s9	6.814	4.292	0.632	3.263	Microcrystalline material
s10	2.65	6.315	0.455	5.581	Small crystals/ Weak diffraction

Measured at 15 sec exposure time; detector distance 60 mm

[a]: Resolution at 1.8 Å; a=29.26 Å, b=50.21 Å, c=52.82 Å, α=90.06°, β=94.66°, γ=90.01°, V=77354 $Å^3$

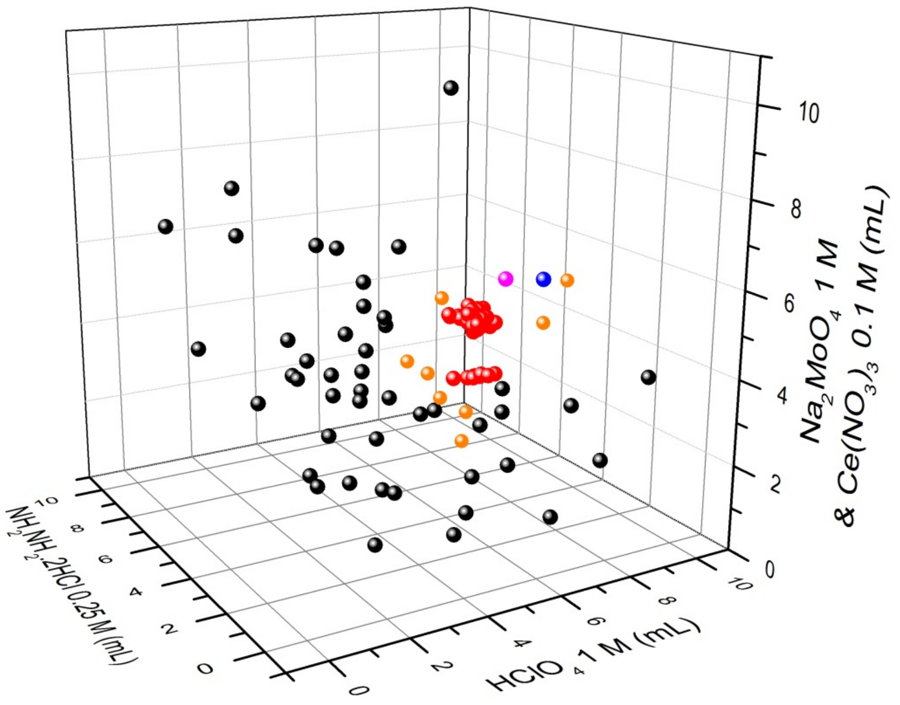

Figure S47: 3D graph of the selected experiments repeated from the algorithm. Coloring code: crystals from initial conditions, red; non-crystals from initial conditions, black; crystals for validation, orange; crystals confirmed as {$Mo_{120}Ce_6$} by single-crystal X-ray analysis, magenta; crystals with unreliable unit cell, blue.

10. ICP validation of the products observed in the crystallization boundaries

The ICP analysis was performed on an Agilent Technologies 5100 ICP-OES for molybdenum, cerium and sodium.

As observed from part 9, when we are in the boundaries of crystallization, it is difficult to isolate and characterize products with single-crystal X-ray diffraction because of the low crystal quality of the material.

For this reason, we decided to use ICP as a qualitative and quantitative assessment tool. We performed the ICP analysis in the experiments described in Table S5 and depicted in Figure S44. Based on the formula $Na_6[Mo_{120}Ce_6O_{366}H_{12}(H_2O)_{78}]\cdot200H_2O$ (**1**) we calculate the following ratios:

Table S10: Theoretical percentages of Mo, Ce and Na based on the formula $Na_6[Mo_{120}Ce_6O_{366}H_{12}(H_2O)_{78}]\cdot200H_2O$ (**1**).

	% Mo	% Ce	% Na	% Mo/% Ce
Compound (**1**)	49.268	3.598	0.59	13.69

The results of this validation can be summarized on Table S11.

Table S11: ICP analysis validation.

Sample	% Mo	% Ce	% Na	% Mo/% Ce
s1	52.181	3.654	0.832	14.281
s2	52.874	3.849	0.646	13.735
s3	49.430	3.495	0.803	14.144
s4	51.554	3.809	0.852	13.534
s5	50.732	3.849	0.732	13.177
s6	50.08	3.545	0.658	14.127
s7	51.229	2.925	0.894	17.512
s8	52.164	2.276	0.835	22.919
s9	53.784	3.869	0.651	13.9
s10	49.977	3.689	1.025	13.548
s11	53.447	2.097	0.927	25.483
s12	51.245	3.845	0.617	13.327

We can observe that samples s7, s8 and s11 have higher values of %Mo/%Ce. In Figure S48 we can observe that these three experiments are located in the outer edges of the boundaries. Additionally, the relevant Ce is lower than both the theoretical value and the values observed in the other samples, something that can be an indication of the existence of Mo species such as {Mo_{154}} which has already been observed (see Figure S46).

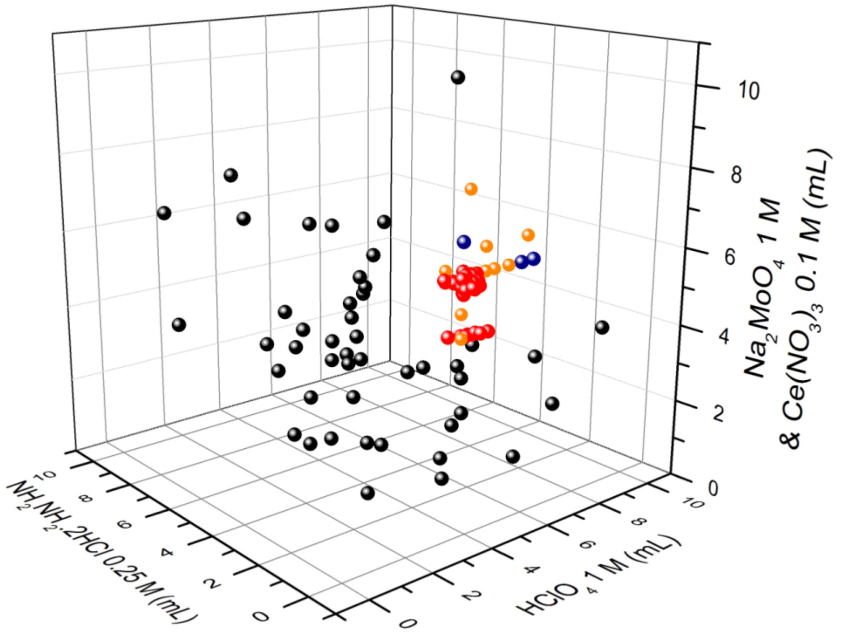

Figure S48: 3D graph of the experiments performed for ICP validation analysis. Coloring code: crystals from initial conditions, red; non-crystals from initial conditions,

black; crystals validated as {$Mo_{120}Ce_6$} by ICP, orange; crystals of samples s7, s8 and s11, blue

In order to check for the stability of our samples in time we performed two experiments; conditions of A belong to the first and bigger cluster of crystallized experiments and conditions of B belong to the second, smaller cluster of crystallized experiments. These samples were left for one day, one week and one month and subsequently ICP analysis was carried out. As we can see from Table S12, the %Mo/%Ce remains relatively constant in time. In sample A after one month, we can observe an increase in %Mo/%Ce and %Na while in the same time there is a decrease in %Ce. This can indicate that after compound (**1**) is finished precipitating, then different compounds like the {Mo_{154}} discussed before can start precipitating.

Table S12: ICP analysis results over a period of time of one day, one week and one month.

Sample	% Mo	% Ce	% Na	% Mo/% Ce	Time
A	49.986	3.818	0.482	13.09	1 day
B	49.771	3.777	0.439	13.18	
A	49.652	3.881	0.543	12.79	1 week
B	50.453	3.902	0.494	12.93	
A	50.127	3.118	0.776	16.08	1 month
B	48.956	3.596	0.555	13.61	

A: H_2O, 3.647 ml; $HClO_4$, 4.739 ml; $NH_2NH_2.2HCl$, 0.777 ml; $Ce(NO_3)_3.6H_2O$ and $Na_2MoO_4.2H_2O$ 5.837 ml & B: H_2O, 5.017 ml; $HClO_4$, 4.742 ml; $NH_2NH_2.2HCl$, 0.631 ml; $Ce(NO_3)_3.6H_2O$ and $Na_2MoO_4.2H_2O$ 4.609 ml

We also performed ICP analysis in samples from the modified synthesis from the platform in bench:

Table S13: ICP analysis results from modified bench synthesis

Modified synthesis	% Mo	% Ce	% Na	% Mo/% Ce
	47.769	3.961	0.542	12.059

11. Quantitative analysis of the strategies

11.1 Principles

In this section we go beyond the qualitative description of the strategies developed by each method and define metrics to quantify: (a) how much each method was able to explore the crystallization zone and (b) how good the data acquired were for building a model, which is to predicting crystallization of future experiments.

This analysis will be based on the data acquired by each method and how criteria of exploration and model quality evolved as more data were acquired.

- To quantify exploration we will only consider the crystals found and estimate how widespread they are distributed in the chemical space. We used two main techniques. The first one, studies the minimal convex volume encompassing the found crystals in the chemical space. The second technique attempts to estimate how similar or different the experiments leading to crystallization were, based on a distance metric in the chemical space.

- To quantify the data quality and their ability to model the crystallization domain, we measure the evolution of the prediction accuracy of a classifier trained using the data acquired by each method. An efficient method will increase its accuracy with less data. We tested different classification algorithms to ensure results are not specific to the SVM classifier used by the algorithm method.

Implementation details can be found on the online code repository: https://github.com/croningp/crystal_active_learning

11.2 Explored space

11.2.1. Number of crystals found

First, we plot how many experiments leading to crystals have been performed (Figure S49) by each method and for each run. This is not directly informative of exploration but provides some useful information when put in perspective with other metrics. We observe that Random runs 1 and 2 found about 5 crystals out of 100 experiments, suggesting that it is not easy to find experiments leading to crystals by mere chance. We also observed that the Human run 1 leaded to almost 50 crystals experiments out of 100. While this might look good, when put in perspective with our analysis of Section 6 it mostly indicates that the experiments were really conservative and close to already known crystallized experiments.

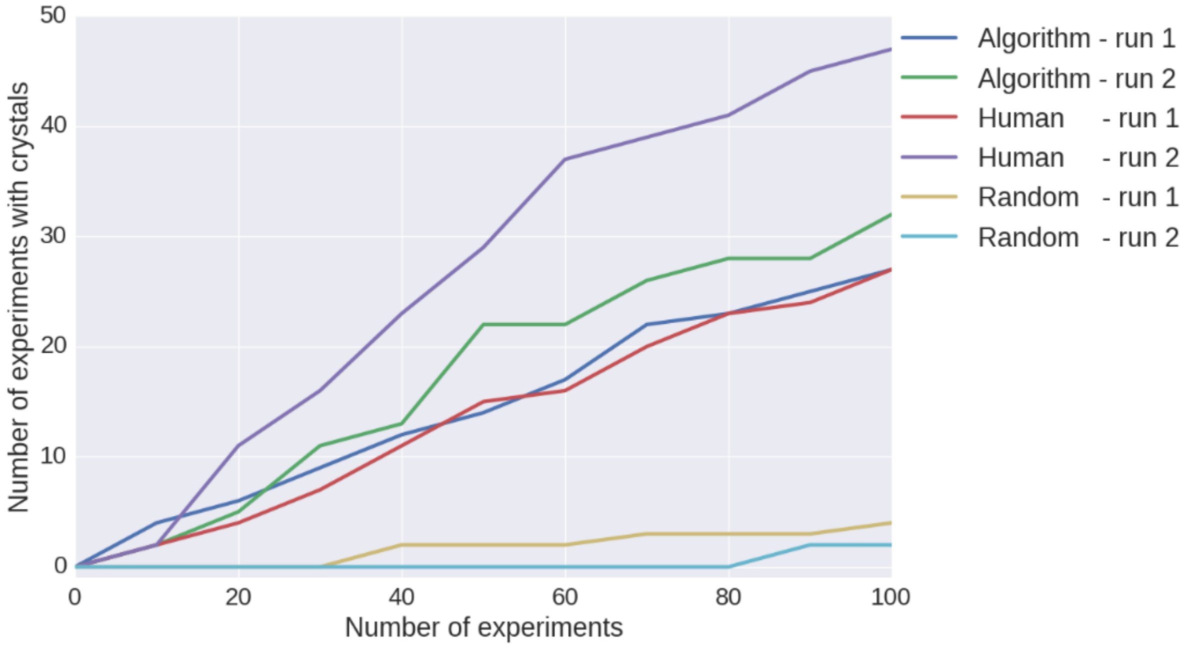

Figure S49: Number of experiments leading to new crystals.

11.2.2. Volume exploration: Convex hull method

Considering the crystallization area as a volume in the parameter space of chemical involved in the experiments, a valuable metric is to estimate how much of the crystallization volume has been explored by each method. But this true volume is unknown to us. An alternative is to compute the volume created by the experiments leading to crystals. One could argue that the bigger this volume, the better the algorithm is at exploring the boundaries between crystal and no-crystal zones.

To do so we compute the volume of the convex hull formed by the experiments leading to crystals. The convex hull is the smallest convex volume that encompasses all of the experimental points in our chemical space. In a 2D space, this process can be illustrated as in Figure 50 and in a 3D space as in Figure S51.

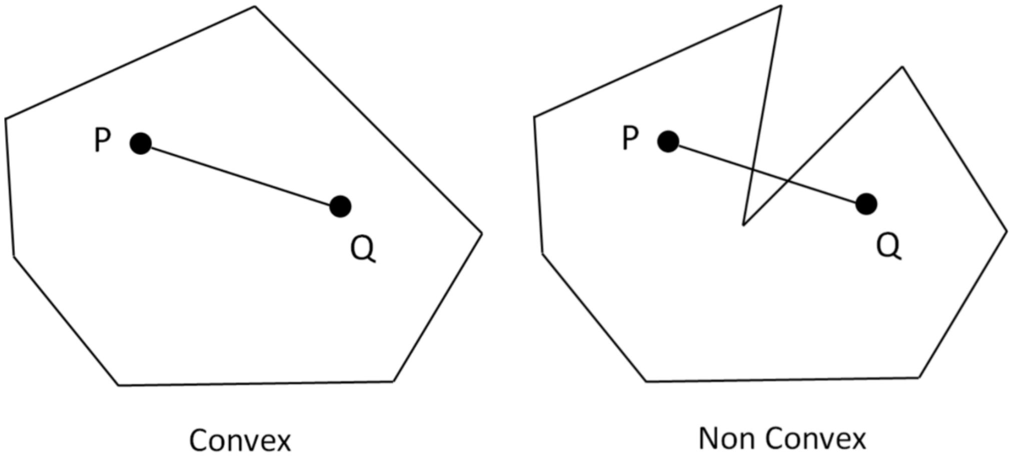

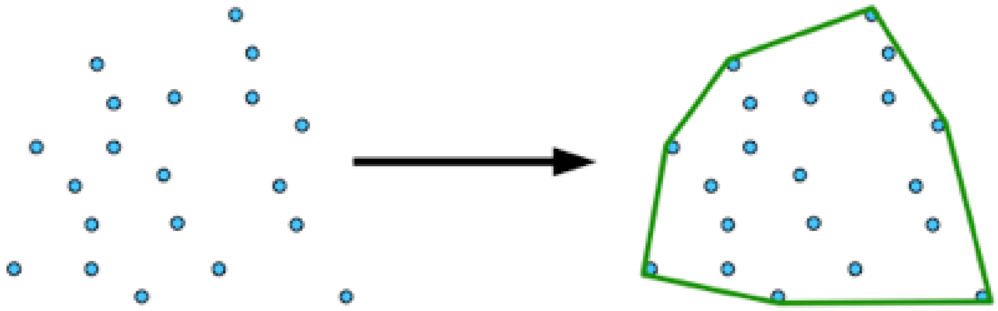

Figure S50: 2D convex hull method visualization.

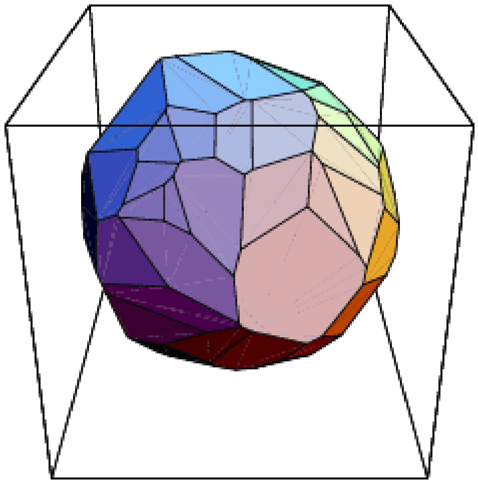

Figure S51: 3D convex hull method visualization. An *n*-dimensional polyhedron is created by the crystal points, where *n* is the number of experimental parameters taking part in the system under study. In our case, *n*=4 representing the volumes of H_2O, $HClO_4$ 1 M, $NH_2NH_2{\cdot}2HCl$ 0.25 M and $Na_2MoO_4{\cdot}2H_2O$ 1 M /$Ce(NO_3)_3{\cdot}6H_2O$ 0.1 M. In the picture *n*=3 for visualization purposes. The cube represents a chemical space formed by three chemicals, e.g. chemical A, chemical B and chemical C. These chemicals react and in certain ratios they form crystals. The coordinates of these formulations can be formulated as a vector of quantities for [A, B, C] and are strickly enclosed by the polyhedron representing the convex hull.

This method was implemented using the scipy.spatial module and its practical implementation can be found in the analysis/explored_volume/convex_hull_method.py file. The results are shown in Figure S52 where the y axis corresponds to a 4-dimensional volume of the convex hull of all crystals points in the parameter space of our 4 reagents. As each parameter is in mL units, strictly speaking the y-axis unit is mL^4, but this has no intuitive meaning and therefore results should simply be interpreted relatively to each other and not as absolute values. The Figure S52 shows that for the Algorithm method, run 1 and run 2 lead to a much wider exploration of the crystallization zone. The human method led also to a wider exploration than a simple random exploration. This is to put in perspective with Figure S49 where the Human method tends to

produce more or similar amount of crystals experiments, yet these experiments seem to be located in a narrow part of the chemical space. The Algorithm seems to be bolder, reaching and finding crystals further out in the chemical space.

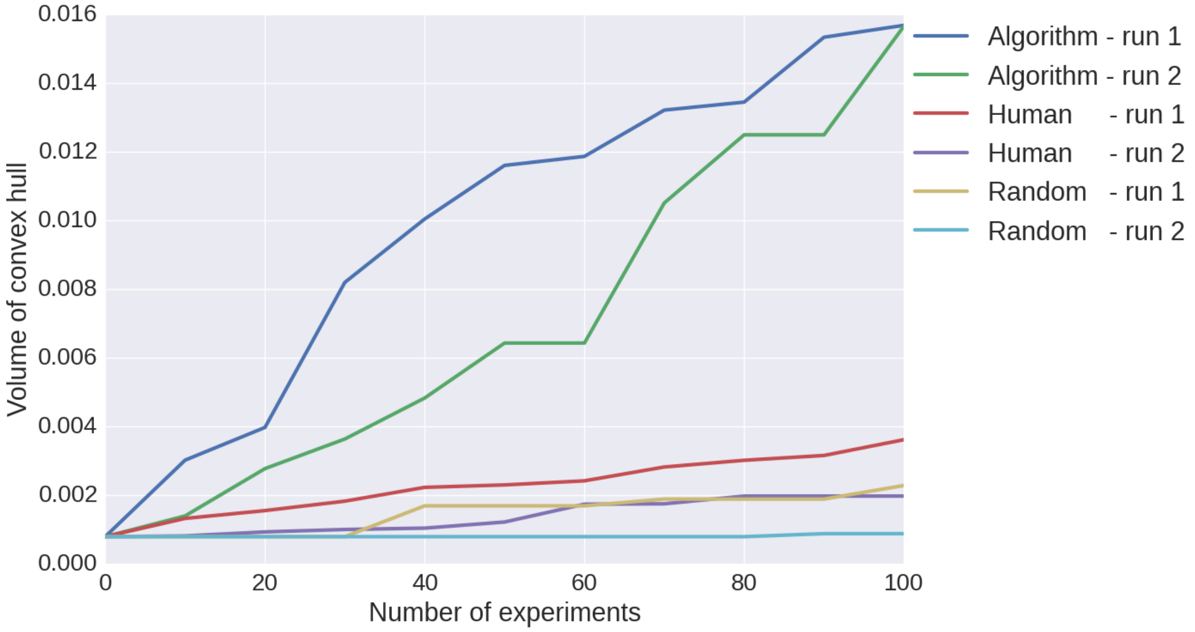

Figure S52: Evolution of the volume of the convex hull of the experiments leading to crystals for each method.

One main limitation of this method is that it considers the volume as convex with all points belonging to a single cluster. In addition, the space of chemicals being 4-dimensional magnifies any differences in terms of volumes, which might explain the substantial difference between methods. For these reasons, we tested a different approach using a similarity metric between experiments forming crystals.

11.2.3. Similarity between experiments

Ideally, we would like to estimate exactly the volume explored by each method. One way is to assign a radius of influence for each point and compute the union of all such n-dimensional spheres. This, however, is a very hard problem and computationally expensive process, especially in a 4-dimensional space as the one of our reagents space[12].

To get around this issue, we flip the problem and count the average number of points within a specific distance of all other points. That is, given an experimental point, how many other experiments lie within a specific radius in the parameters space, measured as a Euclidean distance. This distance is a similarity measure between experiments, a small value indicates similar experiments. First, we need to define a value for the radius parameter, to do so we computed the average ratio of crystals within radius of other crystals over the total number of crystals for each

method and for many radii (Figure S53) on the final dataset, i.e. once all the 100 experiments were collected. First, we observe that for a small value of radii (R), this ratio is close to 0, because two experiments are never exactly the same. Similarly for large values of R, the ratio is 1 because all points are within a contained space. The interesting radii are the ones which are able to capture a difference between each method. We finally note that this plot confirms that the Algorithm 1 and 2 and Human 1 explore more widely the crystallization space. Indeed, the points are on average further away from each other as indicated by, for a given radius, the lower average number of points within a radius of each other.

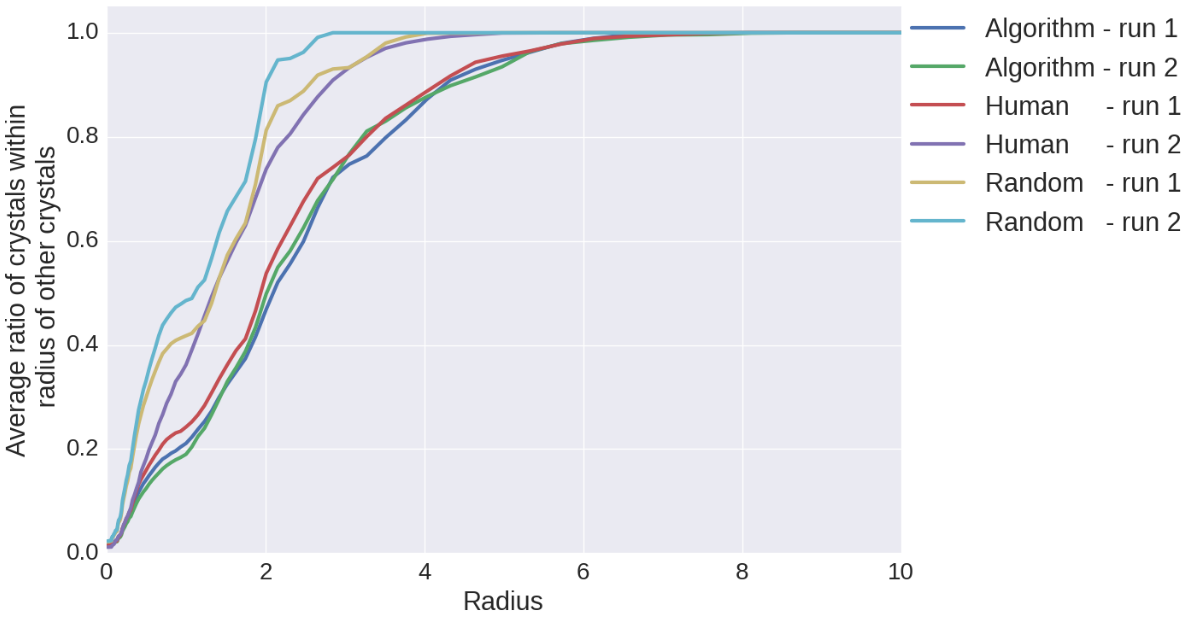

Figure S53: Comparison of numbers of crystals found within a given radius of another crystal.

To select a good value for R, we arbitrarily decided to use the standard deviation of our measure between each method as our metric. The logical is that the radius for which the standard deviation is higher indicates it can capture finer variations between methods. This is because as the standard deviation increases, the different runs of our experiment start to be separated from each other. At the end, a larger deviation from the average value means better separation. Figure S54 shows this measure and indicates that a radius of 2 is optimal given our metrics. We use R=2 in the following to study in more detail the evolution of our similarity measure as our experiments unfold.

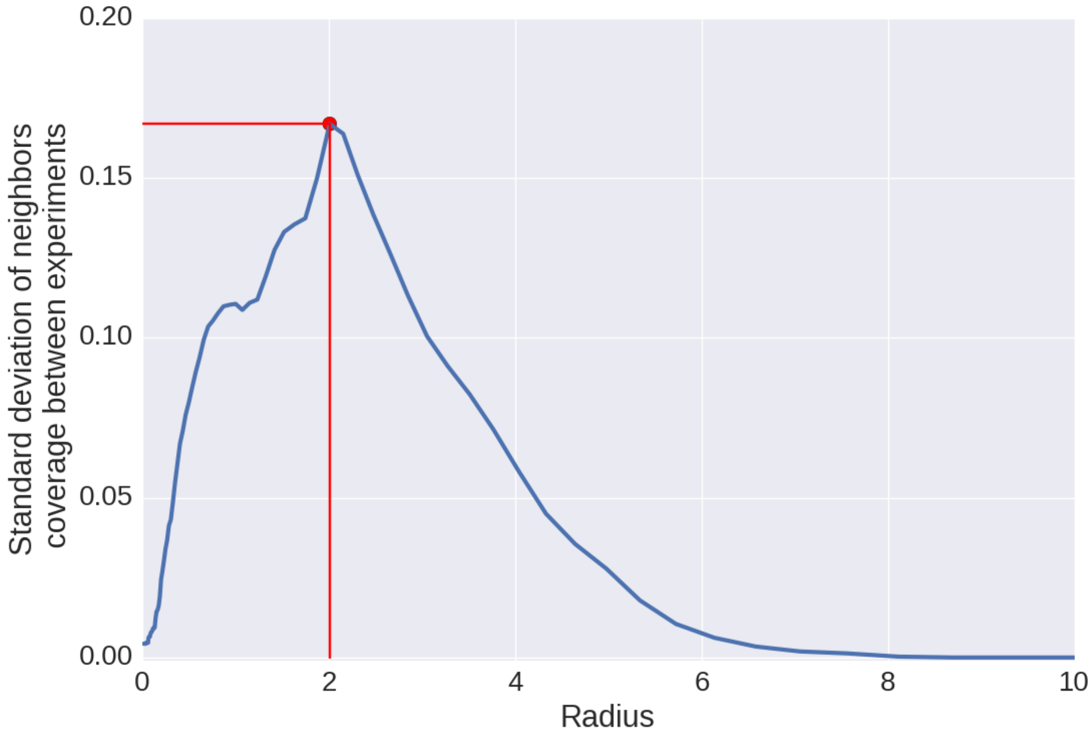

Figure S54: Correlation between radius and standard deviation.

Figure S55 shows the evolution of the average ratio of crystals found within a given distance of other crystals as more experiments are performed by each method. First, we note that in the initial set 90% of the data are within a radius of 2 in the parameter space. This is in line with the visual observation of the initial dataset of section 7. We can observe that Algorithm runs 1 and 2 reduce this ratio quicker than any other method indicating a wider exploration thus less data points in the vicinity of each other. The Human run 1 has a similar dynamic while the Human run 2 is closer to our Random method indicating a rather conservative exploration as has also been noted in section 10.

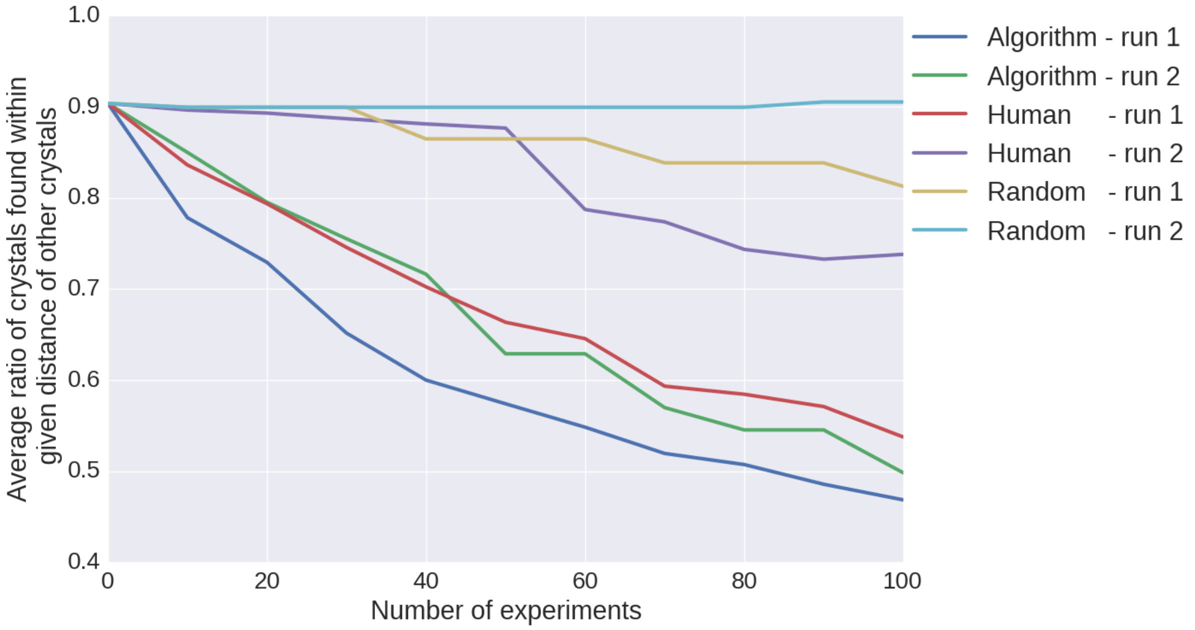

Figure S55: Comparison of numbers of crystals found within a given distance of another crystal.

A final way to represent this similarity analysis is to plot a histogram of the distance between experimental points leading to crystals. For that we compute a matrix of all distances between pairs of experiments leading to crystal in the experimental space. We then plot the distribution of such distances. Intuitively, a better exploration algorithm will have a stronger tail towards higher distances, meaning points are on average more distant from each other. Figure S56 confirms these tendencies shown in previous analysis with the Algorithm 1 and 2 and Human 1 having stronger tailed distribution compared to the other 3 runs.

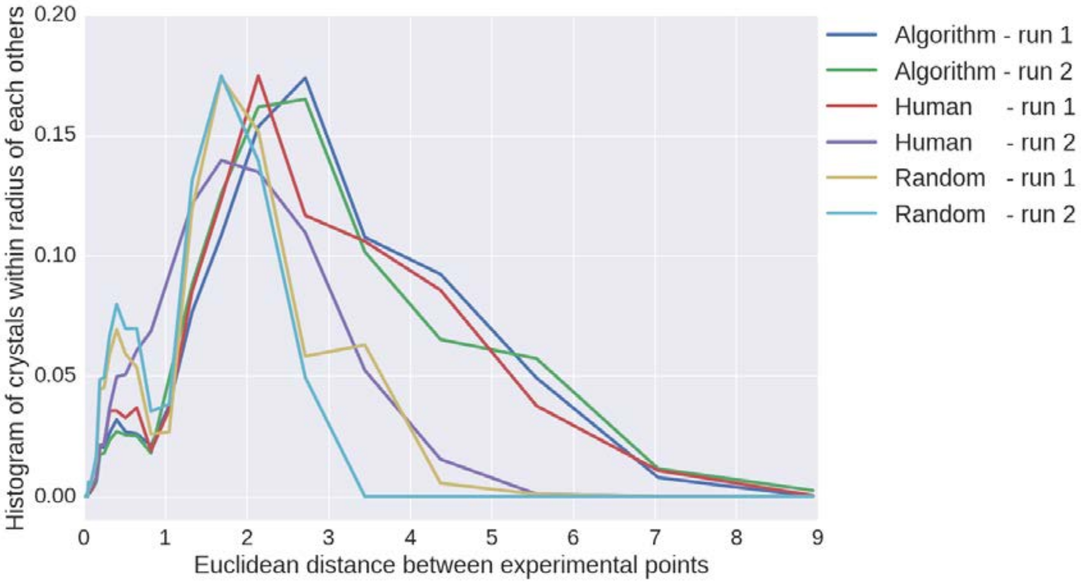

Figure S56: Comparison of the distribution of euclidean distance to neighbours among the three methods

11.3 Data and modelling quality

We define the set of initial experiments as I. A1, A2, H1, H2, R1, R2 are the set of data collected respectively under the Algorithm, the Human and the Random method for both runs 1 and 2. We further define IA1, IA2, IH1, IH2, IR1, and IR2 the respective datasets composed of the initial data plus the subsequent data gathered during an experimental run (i.e. IA1 = {I, A1}). Finally, we define as T the dataset of all experiments performed on the platform that is T = {I, A1, A2, H1, H2, R1, R2}.

11.3.1. Principles and biases

The stated goal of this research is to show how active learning algorithms inspired by research in the field of machine learning can be useful to explore a chemical space in a more cost- and time-effective manner. For completeness, we compared with human experimenters and a baseline random method. Hence, the most important metric is to measure how good the data acquired were to model the area leading to crystals or to no-crystals.

As these areas are not known theoretically our only option is to rely on the data acquired all along our experiments as a way to test the quality of a model. In essence, and with respect to Algorithm run 1, we will train a model using IA1 (all the data collected by Algorithm run 1 and the initial data) and test this model on T (the set of all data ever acquired on the platform). This allows us to test how good our model is to predict the experimental outcome of experiments it has never seen before and that it has never used to infer the model.

In practice, this is implemented by training a classifier on IA1 and testing the percentage of good predictions made on T. The higher the accuracy of the classifier on T, the better the model, hence the more representative and useful the data used to train it.

But such measurements can be biased by the ratio of points representing each class in the testing set (T). For example, in our case T included 181 experiments leading to crystals and 508 experiments that do not lead to crystals (see Table S4), that is about 74% of the test set being of class `no-crystal`. This means that a dummy classifier that predict that all experiments lead to ‘no-crystal’ will in practice have a prediction accuracy of 74% on T. The distribution of labels in T would thus be biasing our metrics towards the deceptive classifier. The solution is to compute two different prediction measurements, one of the class `crystal` and one for the class `no-crystal`, and then average the two to get a single, unbiased, estimate of the accuracy of our model/classifier. In the example above, the dummy always ‘no-crystal’ classifier would have 0% accuracy on ‘crystal’ and 100% accuracy on ‘no-crystal’ for a global accuracy of 50%, something that is no better than pure luck.

Table S4: Summary of crystallization data obtained from the experiments

	Initial data		Uncertainty algorithm		Random search		Human experimenter	
	crystals	no-crystals	crystals	no-crystals	crystals	no-crystals	crystals	no-crystals
Run 1	43	46	27	73	4	96	26	74
Run 2			32	68	2	98	47	53

In total: 689 experiments/ 181 crystals/ 508 non-crystals; for each procedure (algorithm, humans, random) and at each run, 100 experiments were performed with the platform

All results reported are corrected for this bias, hence any value above 50% indicates that the classifier/model captures at least some relevant aspects of the system. The higher the prediction accuracy is, the better the model/classifier, hence the better/more representative the data.

11.3.2. Comparing methods

We compared three different classifiers: SVM with RBF kernel[7], RandomForest[13], and Adaboost[14] on DecisionTree[15]; all implemented within the scikit-learn python library[9]. All three classifiers are able to capture non-linear decision lines between classes. It is also important to check other classifiers than the SVM because SVM was used in the active learning algorithm method described in Section 6, therefore we might have collected data solely tailored to the model built by the SVM classifier. Using two other classifiers allows us to verify that the data gathered are actually useful and meaningful for other modelling methods.

Figures S57, S58, S59 show, respectively for SVM, RandomForest and Adaboost, the evolution of the prediction accuracy of each classifier trained on the data collected by each method for each run. 10-fold cross validation on the full dataset was used to select the set of parameters for each classifier (see Table S14). The same trends appear on all three plots; the machine-learning algorithm was able to collect better quality data and improved its classification accuracy the most. In comparison, the humans showed a less significant improvement and the random did not improve in accuracy. The fact that the model computed using the human method improves less than with the algorithm indicate that the humans did not collect as useful information as the algorithm. This is even more striking with the random method that provided no additional information.

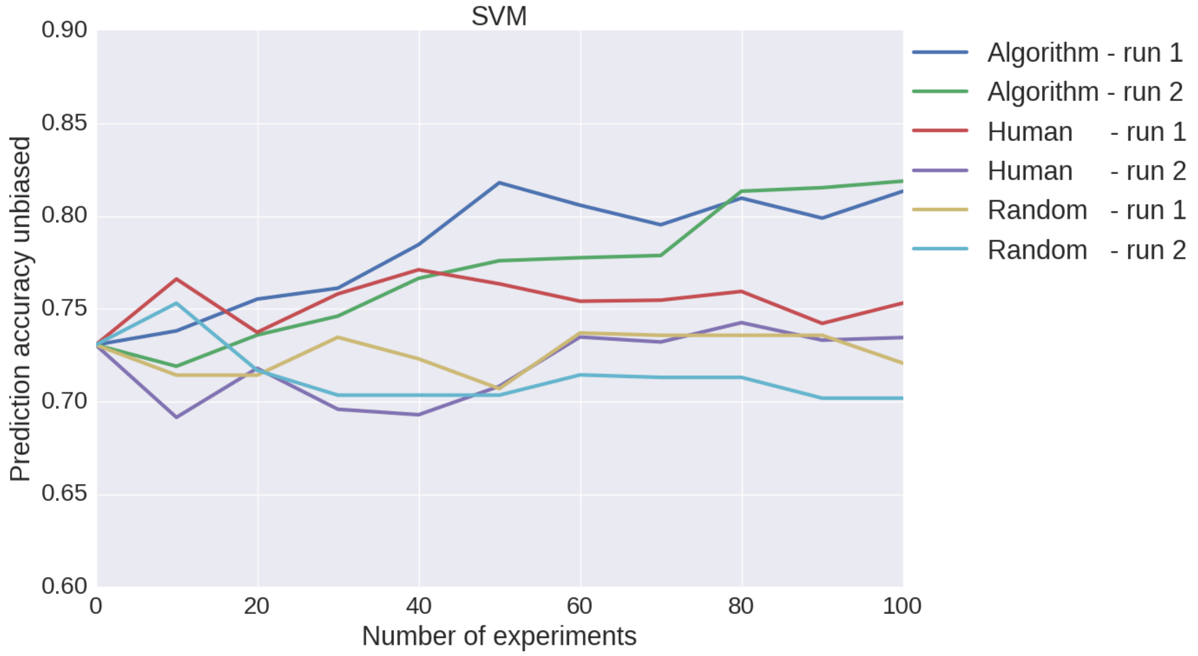

Figure S57: Average for the prediction accuracies between the classes of crystals and non-crystals for the three methods using grid searching of the best set of parameters in the full data set.

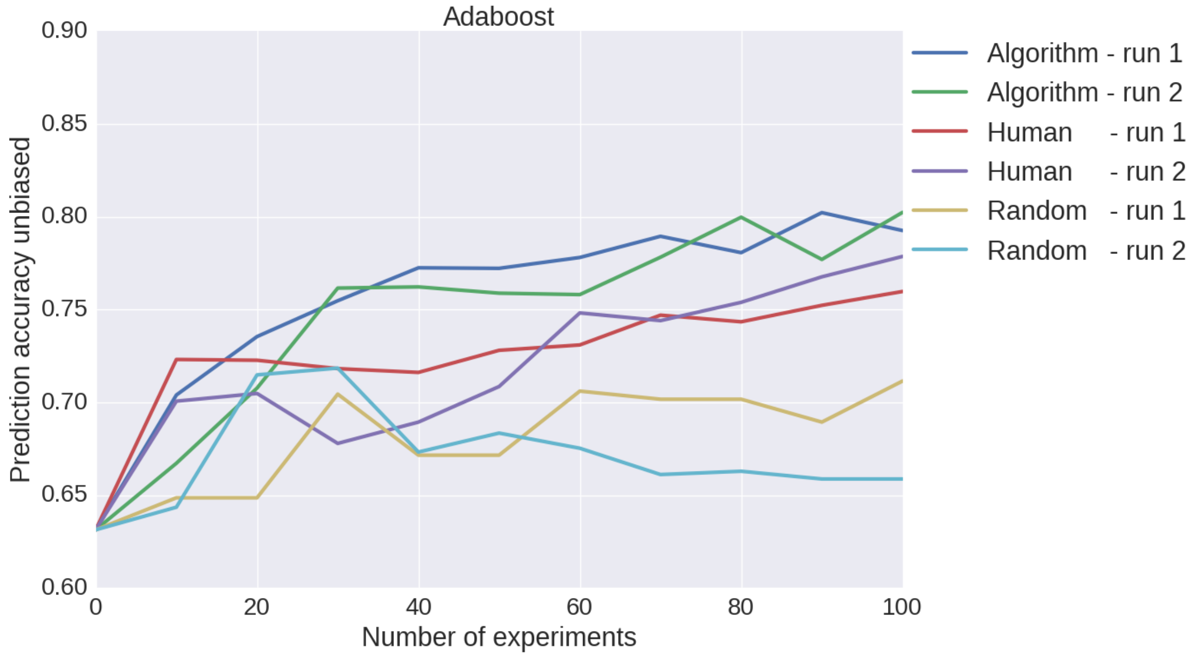

Figure S58: Average for the prediction accuracies between the classes of crystals and non-crystals for the three methods using grid searching of the best set of parameters in the full data set.

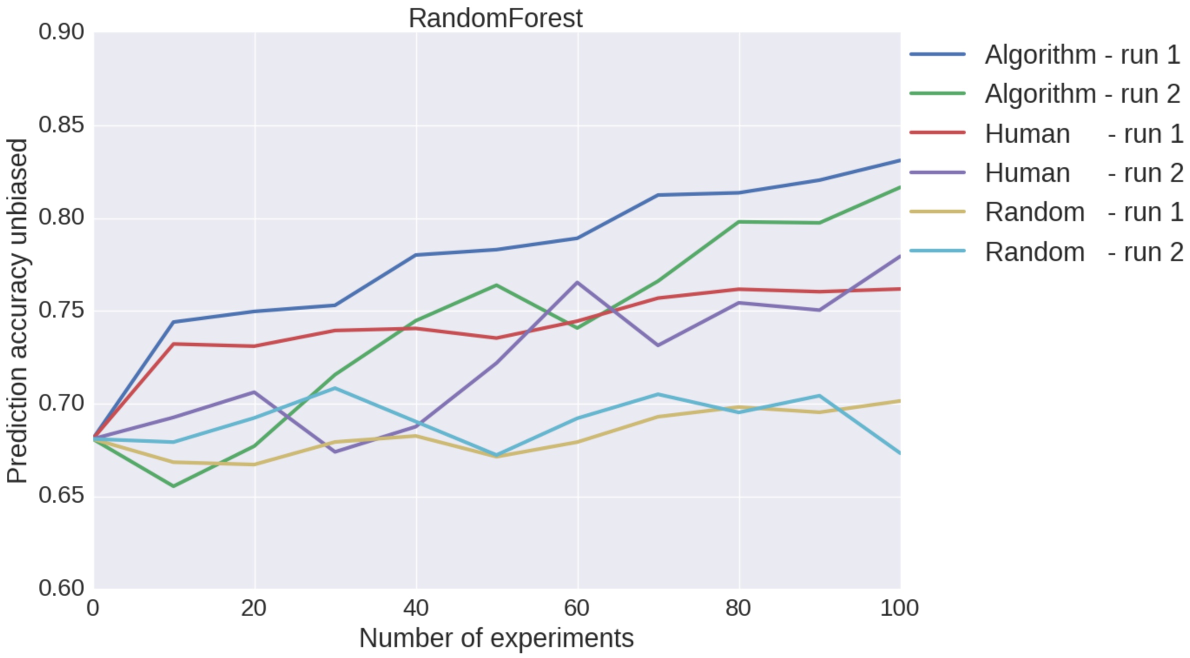

Figure S59: Average for the prediction accuracies between the classes of crystals and non-crystals for the three methods using grid searching of the best set of parameters in the full data set.

Table S14: Prediction quality at the end of 100 experiments for three methods.

	SVM[a]	Adaboost[b]	Random Forest[c]
Algorithm_0	0.813	0.793	0.831
Algorithm _1	0.819	0.802	0.817
Human_0	0.753	0.760	0.753
Human_1	0.734	0.779	0.734
Random_0	0.721	0.711	0.701
Random_1	0.702	0.659	0.673

[a] kernel: rbf; C: 100.0; gamma: $10^{-3/2}$, [b] number of estimators: 50, [c] number of estimators: 100

12. References

[1] G. M. Sheldrick, *Acta Crystallogr. Sect. A*, **1990**, *46*, 467-473

[2] G. M. Sheldrick, *Acta Crystallogr. Sect. C*, **2015**, *71*, 3-8

[3] L. J. Farrugia, *J. Appl. Cryst.*, **1999**, *32*, 837-838

[4] E. Alpaydin in *Introduction to machine learning*, MIT press, **2014**

[5] B. Settles in *Active learning literature survey*, University of Wisconsin, Madison, **2010**, *52*, pp. 55-66

[6] D. D. Lewis, W. A. Gale in *Proceedings of the 17th annual international ACM SIGIR conference on Research and development in information retrieval*, Springer-Verlag New York, Inc., **1994**, pp. 3-12

[7] N. Cristianini, J. Shawe-Taylor in *An introduction to support vector machines and other kernel-based learning methods*, Cambridge university press, **2000**

[8] J. Platt, *Advances in large margin classifiers*, **1999**, *10*(3), 61-74

[9] F. Pedregosa, G. Varoquaux, A. Gramfort, V. Michel, B. Thirion, O. Grisel, J. Vanderplas, *Journal of Machine Learning Research*, **2011**, *12*, 2825-2830

[10] C. E. Shannon, *ACM SIGMOBILE Mobile Computing and Communications Review*, **2001** ,*5*(1), 3-55

[11] S. Thrun, W. Burgard, D. Fox, in *Probabilistic robotics*, MIT press, **2005**

[12] C. Frederic, H. Kanhere, S. Loriot, *ACM Transactions on Mathematical Software (TOMS)*, **2011**, *38.1*: 3

[13] T. K. Ho in *Proceedings of the Third International Conference on Document Analysis and Recognition*, IEEE, Vol. 1, **1995**, pp. 278-282

[14] Y. Freund, R. Schapire, N. Abe, *Journal-Japanese Society For Artificial Intelligence,* **1999**, *14.771-780*: 1612

[15] S. R. Safavian, D. Landgrebe, *IEEE transactions on systems, man, and cybernetics*, **1991**, *21.3*, 660-674
